# Bioinspired nanozyme-exoskeletonized yeast-derived polysaccharides for treating comorbid anxiety and depression in inflammatory bowel disease

**DOI:** 10.1038/s41467-026-78633-3

**Published:** 2026-10-14

**Authors:** Fan Zhang, Lei Chen, Shengfu Wang, Xinyuan Chen, Kaiyi Du, Fuqiang Song, Linyuan Jiang, Yanzhen Feng, Siting Du, Tianyao Jiang, Yong Liu, Jian Xiao

**Affiliations:** 1 Department of Wound Healing, The First Affiliated Hospital of Wenzhou Medical University, Wenzhou, China; 2 Oujiang Laboratory (Zhejiang Lab for Regenerative Medicine, Vision and Brain Health), School of Pharmaceutical Sciences, Wenzhou Medical University, Wenzhou, China; 3 State Key Laboratory of Medicinal Chemical Biology, Academy for Advanced Interdisciplinary Studies, Nankai International Advanced Research Institute (Shenzhen Futian), Nankai University, Tianjin, China

**Keywords:** Biomedical materials, Inflammatory bowel disease, Nanoparticles

## Abstract

Inflammatory bowel disease (IBD) is a chronic condition frequently accompanied by psychological comorbidities, such as anxiety and depression. Although current treatments may alleviate some of these symptoms, the intricate pathogenesis of these comorbidities highlights an urgent need for therapeutic strategies with combined anti-inflammatory, barrier-reparative, and microbiota-modulating effects. Herein, we develop a series of nanozyme-exoskeletonized yeast-derived polysaccharides (NZEP) by integrating nanozymes with yeast-derived polysaccharides using an insect sclerotization-inspired strategy. Among them, NZEP^*TA-Ce*^, with the highest degree of nanozyme-exoskeletonization, is capable of disrupting the inflammatory vicious cycle by inhibiting MAPK and NF-κB-mediated inflammatory signaling pathways. Oral administration of NZEP^*TA-Ce*^ in mice enables effective retention and accumulation in inflamed colonic regions, reverses the homeostasis imbalance of intestinal inflammation and promotes intestinal barrier repair. Furthermore, NZEP^*TA-Ce*^ treatment reverses gut microbiota dysbiosis and optimizes the composition of microbial metabolites, thereby alleviating anxiety and depressive symptoms associated with IBD. This study provides a safe and promising therapeutic strategy for the management of IBD patients and their associated psychological comorbidities.

## Abstract (web summary)

The treatment of comorbid anxiety and depression in inflammatory bowel disease (IBD) is challenging. Here, the authors develop a bioinspired nanozyme-exoskeletonized yeast polysaccharide that targets intestinal inflammation, scavenges reactive oxygen species, restores immune and microbial homeostasis, repairs the intestinal barrier, and alleviates anxiety- and depression-like behaviors in IBD models.

## Introduction

Inflammatory bowel disease (IBD) is a chronic, relapsing immune-mediated disorder primarily affecting the ileum, rectum, and colon, with Crohn’s disease and ulcerative colitis being its main subtypes^1,2^. Patients are typically diagnosed in early adulthood, but chronic inflammation persists throughout life. The global burden of IBD remains a persistent health issue. The impact of IBD extends beyond the gastrointestinal tract, as patients often experience psychological comorbidities, such as anxiety and depression^3^. IBD patients are at an increased risk of developing depression and anxiety compared to the general population, with up to one-third of IBD patients experiencing anxiety symptoms and one-fourth experiencing depressive symptoms^4^. Comorbid anxiety and depression in IBD can significantly reduce patients’ quality of life, complicate clinical management, and increase the frequency of active disease^5,6^. Recent studies have found that IBD patients experience a reduction in depression and anxiety symptoms following treatment with TNF inhibitors or antidepressants^7,8^. However, it remains unclear whether this effect is directly related to the improvement in IBD activity. Given the complex pathogenesis of comorbid anxiety and depression in IBD, research and treatment in this field face numerous challenges. Therefore, there is an urgent need to develop new therapeutic and intervention strategies.

Recent research highlights that the mechanisms underlying comorbid anxiety and depression in IBD are primarily centered on the gut-brain axis, a complex network that governs bidirectional communication between the gut and the brain. One characteristic of IBD is the disruption of inflammatory homeostasis within the gut environment, often accompanied by an impaired gut vascular barrier. This impairment allows inflammatory mediators and pathogen-associated molecular patterns, such as lipopolysaccharides (LPS), to enter the systemic circulation and reach the brain, potentially leading to the closure of the choroid plexus and the manifestation of abnormal anxiety-like behaviors^9^. Furthermore, inflammatory mediators in the systemic circulation may breach the blood-brain barrier, infiltrating critical brain regions such as the hippocampus, thereby contributing to the progression of psychiatric disorders^10^. Additionally, the gut microbiota exerts a significant influence on brain physiological functions via the gut-brain axis^11,12^. Dysbiosis of the gut microbiota, a common feature in IBD patients, is characterized by alterations in microbial diversity resulting from a disrupted microbiota balance and corresponding functional changes. Numerous studies have demonstrated a strong association between gut microbiota dysbiosis and severe psychiatric disorders, including depression, anxiety, and psychosis^13^. Consequently, reversing intestinal inflammation, repairing the intestinal barrier, and restoring microbial balance are critical approaches for addressing comorbid anxiety and depression in IBD patients.

Yeast-derived polysaccharides (such as yeast glucan particles, YGPs) possess immunomodulatory functions and are widely used as therapeutic agents to activate or suppress the immune system^14,15^. β-glucan and chitin are common polysaccharides found in the fungal cell wall. Upon binding with β-glucan, immune cells exhibit enhanced activity of intracellular antioxidant enzymes, such as catalase (CAT) and superoxide dismutase (SOD), which bolsters their capacity to scavenge reactive oxygen species (ROS), thereby mitigating ROS-induced tissue damage^16,17^. Additionally, fungal mannan in the fungal cell wall can activate mannose receptors (MR/CD206) on M0 immune cells, leading to their transformation into M2 phenotype macrophages and upregulation of IL-4-related cytokines, producing an anti-inflammatory effect^18^. Moreover, YGPs, as a prebiotic, could selectively enhance the growth of beneficial gut bacteria (*Bacteroides*) and contribute to the regulation of the gut microbiome^19,20^. Based on these functions, YGPs may serve as a potential candidate for combating comorbid anxiety and depression in IBD patients. However, during active inflammation of IBD, the enhancement of antioxidant enzyme activity in immune cells by YGPs is not sufficient to reverse the homeostasis imbalance of intestinal inflammation. In recent years, the emerging field of nanozymes has shown promise in efficiently and rapidly scavenging ROS at inflammation sites by mimicking the activity of natural enzymes^21,22^. Nanozymes have already been applied in the treatment of various inflammation-related diseases^23,24^. Therefore, we hypothesized that combining nanozymes with YGPs could synergize the inherent advantages of both biomaterials, effectively reversing the inflammatory environment and microbial dysbiosis, ultimately achieving the treatment of comorbid anxiety and depression in IBD.

In this study, we propose an insect sclerotization-inspired strategy to integrate nanozymes with YGPs, constructing nanozyme-exoskeletonized yeast-derived polysaccharides (NZEP) for the treatment of comorbid anxiety and depression in IBD.

**Fig. 1:**
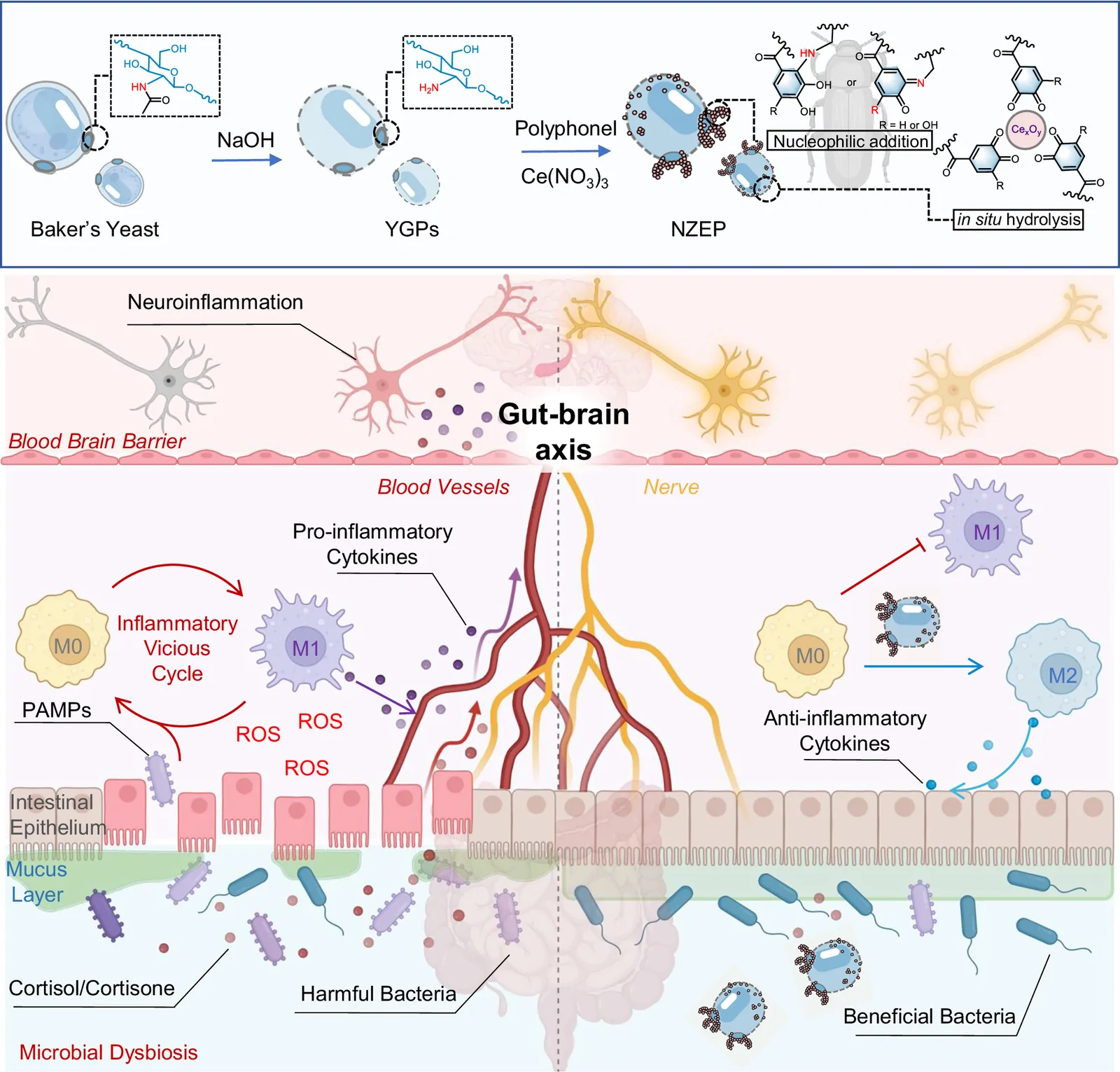
The insect sclerotization-inspired strategy to integrate nanozymes with YGPs, constructing nanozyme-exoskeletonized yeast-derived polysaccharides (NZEP) for the treatment of comorbid anxiety and depression in IBD (created in BioRender. Zhang, F. (2026) https://BioRender.com/1rcpn6n).

NZEP exhibits a superior ability to disrupt the inflammatory vicious cycle by inhibiting mitogen-activated protein kinase (MAPK) and nuclear factor-κB (NF-κB)-mediated inflammatory signaling pathways. The orally administered NZEP could effectively retain and accumulate in inflamed regions of the intestine, reversing the IBD-induced homeostasis imbalance of intestinal inflammation and promoting intestinal barrier repair. Furthermore, NZEP could regulate the abundance and diversity of gut microbiota, reverse microbial dysbiosis, and optimize the composition of microbial metabolites, thereby alleviating anxiety and depressive symptoms associated with IBD (Fig. 1). This work presents a promising therapeutic approach for modulating the microbiota-gut-brain axis in IBD and patients with related psychological comorbidities.

## Results

### Synthesis and characterization of nanozyme-exoskeletonized yeast-derived polysaccharides

**Fig. 2:**
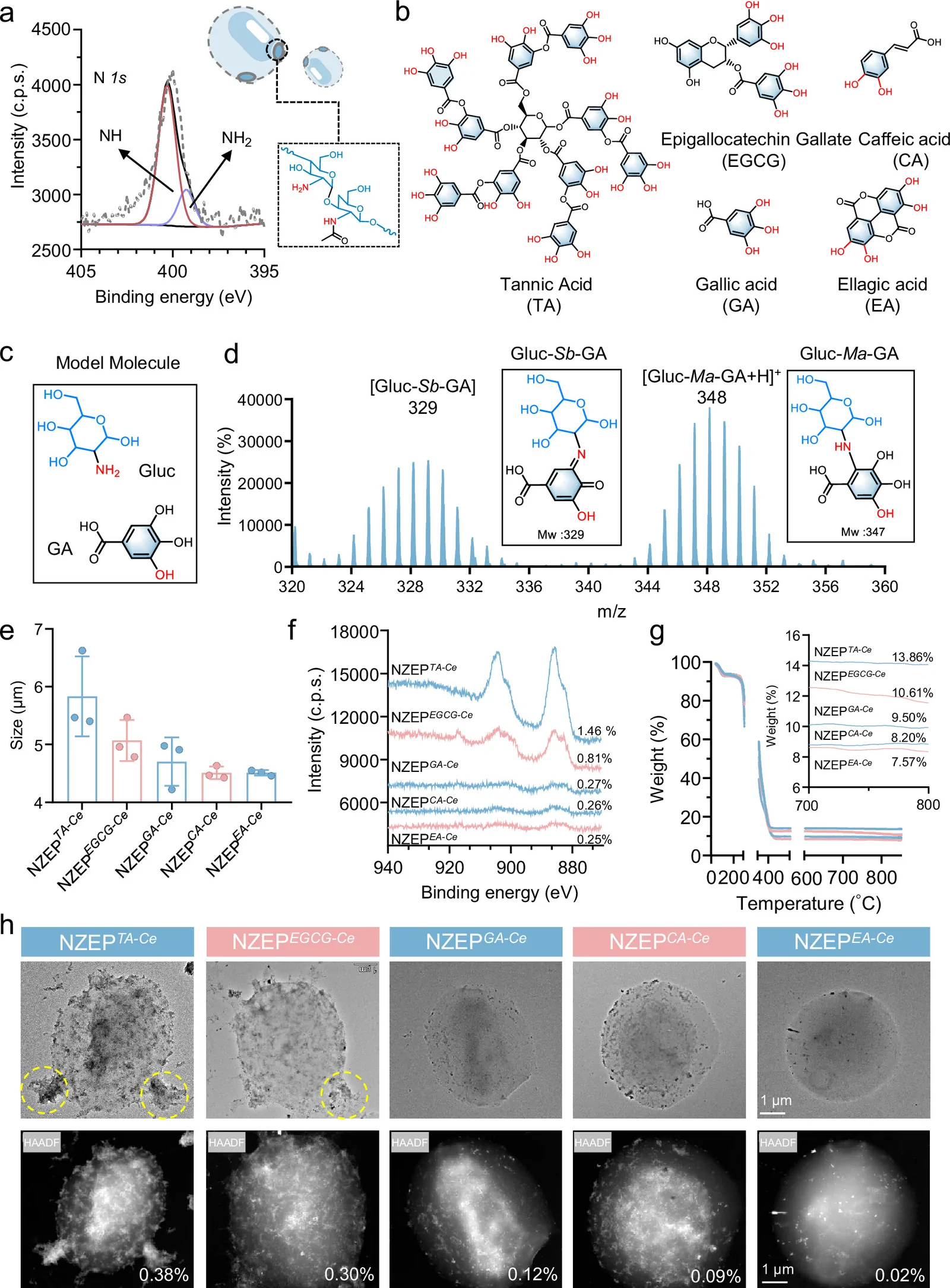
Fabrication and characterization of NZEP^*MPN-Ce*^. **a** XPS spectrum of YGPs. **b** Chemical structural formulae of five polyphenolic compounds. **c** Chemical structural formulae of the model molecule. **d** MALDI-TOF/MS spectrometry of Glu-*Sb*-GA and Glu-*Ma*-GA. **e** Size distribution of NZEP^*MPN-Ce*^ measured by DLS, expressed as hydrodynamic diameter (*n* = 3 independently prepared samples, and data are presented as mean ± SD). **f** XPS spectrum of NZEP^*MPN-Ce*^. **g** Thermogravimetric analysis test of various NZEP^*MPN-Ce*^. **h** TEM and HAADF images of the various NZEP^*MPN-Ce*^. The yellow dashed circles indicate budding sites. Scale bars, 1 μm. Three independent experiments were performed, and representative results are shown in (**h**).

Purification of YGPs with a diameter of approximately 4.060 μm is achieved from *S. cerevisiae* using acid and alkaline extraction methods to remove intracellular and cell wall polysaccharides (Supplementary Fig. 1a–c)^25^. The purified YGPs were validated through transmission electron microscopy (TEM) to confirm their hollow structure and intact rigid skeletal framework. Furthermore, energy dispersive spectroscopy (EDS) element mapping confirms that the YGPs are composed of *β*-glucan and chitin (the N element is attributed to chitin) (Supplementary Fig. 1d). During the strong alkaline treatment stage in the purification process, chitin undergoes deacetylation to transform into chitosan. Characterization of the YGPs through X-ray photoelectron spectroscopy (XPS) revealed the presence of -NH_2_ signal peaks (399.2 eV) in the high-resolution N1*s* XPS spectrum, confirming the progress of the deacetylation reaction (Fig. 2a). The presence of -NH_2_ groups on the surface of YGPs inspired us to develop a method for NZEP employing an insect sclerotization-inspired strategy (phenol oxidases-assisted phenol-polyamine chemistry). To confirm if the -NH_2_ on YGPs surfaces can crosslink with polyphenols (tannic acid (TA), epigallocatechin gallate (EGCG), gallic acid (GA), caffeic acid (CA), Ellagic acid (EA)) through a phenol-amine chemical reaction (Fig. 2b), we selected glucosamine (Gluc) and GA as model molecules for validation (Fig. 2c). Under alkaline conditions, catechol hydroxyl groups are oxidized by oxygen to form quinone structures. Subsequently, they react with amino groups through nucleophilic addition (Schiff base or Michael addition) reactions (Supplementary Fig. 2a)^26,27^. We verified the nucleophilic addition products through hydrogen nuclear magnetic resonance (^1^H NMR) spectroscopy (Supplementary Fig. 2b). Compared to the ^1^H NMR spectrum of quinone, the reaction mixture exhibits the emergence of two new sets of peaks (H1 and H2, H3 and H4) with reduced peak signals (Hb, Hc), suggesting the simultaneous occurrence of Michael addition and Schiff base reactions throughout the reaction process. Then, the chemical structures of these nucleophilic addition products were further confirmed by matrix-assisted laser desorption/ionization-time of flight (MALDI-TOF) mass spectrometry (MS), and MALDI-TOF/MS spectrometry data analysis revealed the presence of molecular ion peaks corresponding to both Schiff base (Glu-*Sb*-GA) and Michael addition (Glu-*Ma*-GA) reaction products in the resulting compounds (Fig. 2d).

Based on the mechanism described above, we developed a one-step biomimetic modification strategy for constructing NZEP. Polyphenol and Ce (NO_3_)_3_ were selected as raw materials to construct the nanozyme-exoskeleton. Firstly, the as-prepared NZEP were evaluated by dynamic light scattering (DLS). The hydrodynamic diameters of these NZEP ranges from 4.469 to 6.522 μm, with a negatively charged surface (Fig. 2e and Supplementary Fig. 3). Upon comparison, it was found that the NZEP constructed by TA (NZEP^*TA-Ce*^) exhibited the largest hydrodynamic diameter, suggesting a higher degree of nanozyme-exoskeletonization by TA. To further quantitatively characterize the degree of nanozyme-exoskeletonization on NZEP, the NZEP was characterized using XPS (Fig. 2f). By comparing the atomic percentages of cerium elements (Ce), it was observed that, apart from EA, the atomic percentage of cerium elements is positively correlated with the number of polyphenol groups. Also, the thermogravimetric analysis (TGA) of those NZEP were measured in ambient air, the residual masses of NZEP^*TA-Ce*^ and NZEP formed by EGCG (NZEP^*EGCG-Ce*^) were measured to be 13.86% and 10.61%, respectively. The residual masses of NZEP formed by GA (NZEP^*GA-Ce*^) and CA (NZEP^*CA-Ce*^) were measured to be 9.50% and 7.57%, respectively. This could be attributed to the lower number of phenolic hydroxyl groups, leading to a reduced degree of nanozyme-exoskeletonization (Fig. 2g). It is worth noting that, despite EA having a higher number of phenolic hydroxyl groups in its chemical structure, it cannot effectively achieve the nanozyme-exoskeletonization process on YGPs. This result may be attributed to its strong intramolecular hydrogen bonding and poor water solubility. Further observation of the morphology of NZEP through TEM reveals that the surface of YGPs is covered with a coating formed by MPN-Ce, with the morphology remaining essentially unchanged (Fig. 2h). Additionally, by utilizing EDS elemental mapping (Supplementary Fig. 4) and high-angle annular dark-field (HAADF) imaging to determine the cerium element, further intuitively verified the above phenomenon.

In an alkaline environment, through nucleophilic addition and in situ hydrolysis processes^28,29^ (Supplementary Fig. 5a), TA-Ce could be effectively loaded onto the surface of the YGPs. To assess the specificity of the exoskeletonization process mediated by TA and Ce^3+^, we investigated the role of different metal ions. Ce^3+^, Zn^2+^, Pd^2+^, Mn^2+^, Cu^2+^, and Fe^2+^ were individually used as metal ion sources to synthesize NZEP with different TA-metal exoskeletonized structures (NZEP^*TA-M*^) using the same preparation method. TEM and EDS mapping analyses of the metal distribution and content on the NZEP surface demonstrated that, apart from NZEP^*TA-Ce*^, other NZEP^*TA-M*^ variants exhibited minimal metal incorporation (Supplementary Fig. 5b). DLS analysis showed that, with the exception of NZEP^*TA-Ce*^, the other NZEP^*TA-M*^ formulations did not display significant differences from bare YGPs (Supplementary Fig. 5c, d). This indicated that under alkaline conditions, Zn^2+^, Pd^2+^, Mn^2+^, Cu^2+^, and Fe^2+^ failed to deposit onto the YGPs surface via in situ hydrolysis. Under alkaline conditions, the -NH₂ groups on YGPs interact with polyphenols like TA to form a stable network, while TA’s hydroxyl groups oxidize, complex with Ce^3+^, and undergo in situ hydrolysis, collectively leading to the formation of TA-Ce. Two interlinked processes together facilitated the formation of the tannic acid-cerium nanozyme exoskeleton (NZEP^*TA-Ce*^). These findings demonstrated that NZEP^*TA-Ce*^ exhibits distinct exoskeletonization characteristics. The well-defined exoskeleton cannot be easily replicated by substituting TA with other polyphenols or Ce^3+^ with different metal ions, underscoring the specificity of the TA-Ce interaction in establishing a stable nanozyme exoskeleton.

Of note, there was a higher enrichment of MPN-Ce at the budding sites, and compared to other polyphenols, NZEP constructed with TA exhibited greater enrichment of TA-Ce at the budding sites (Fig. 2h and Supplementary Fig. 6a). This is likely due to a higher chitosan content at the budding sites compared to other locations^30^. The surface chemical composition and valence states of NZEP^*TA-Ce*^ were further evaluated by XPS (Supplementary Fig. 6b, c). Among them, the proportions of C, O, N, and Ce elements in NZEP^*TA-Ce*^ were 57.56%, 39.84%, 1.15%, and 1.46%, respectively. The ratio of tri- to tetravalent cerium (Ce^3+^/Ce^4+^) was 55.6%:44.4%, indicating that the cerium oxide in the NZEP^*TA-Ce*^ could act as a superoxide dismutase to eliminate intracellular superoxide and free radicals, playing an antioxidative role^31^. To elucidate the distribution of Ce within NZEP^*TA-Ce*^, cross-sectional TEM and HAADF imaging were conducted. The analyses revealed that TA-Ce is predominantly confined to the surface of NZEP^*TA-Ce*^, with minimal presence in the interior, supporting the proposed exoskeletal structural model (Supplementary Fig. 6d). TEM images showed the TA-Ce had a nanoparticle morphology, with C, O, and Ce elements evenly distributed. It tends to aggregate into irregular amorphous structures, which contrasts sharply with the distinct individual particle morphology of NZEP^*TA-Ce*^ (Supplementary Fig. 7). To further quantify the nanozyme-exoskeleton in NZEP^*TA-Ce*^, TGA analysis revealed that TA-Ce constituted approximately 41.56% of NZEP^*TA-Ce*^, while the remaining 58.44% was composed of YGPs (Supplementary Fig. 8). NZEP^*TA-Ce*^ exhibited excellent stability in simulated gastrointestinal (GI) fluids, with no significant changes in particle size, nanozyme activity, or morphology, confirming its ability to maintain the integrity of the TA-Ce exoskeleton and exert ROS-scavenging effects at inflamed intestinal sites (Supplementary Fig. 9). We have found that TA-Ce was similarly stable in gastric fluid, retaining its morphology, elemental distribution, and enzymatic activity to ensure sustained ROS scavenging once it reaches the intestine. (Supplementary Fig. 10).

### Antioxidant and macrophage cellular targeting capabilities of NZEP^*TA-Ce*^

**Fig. 3:**
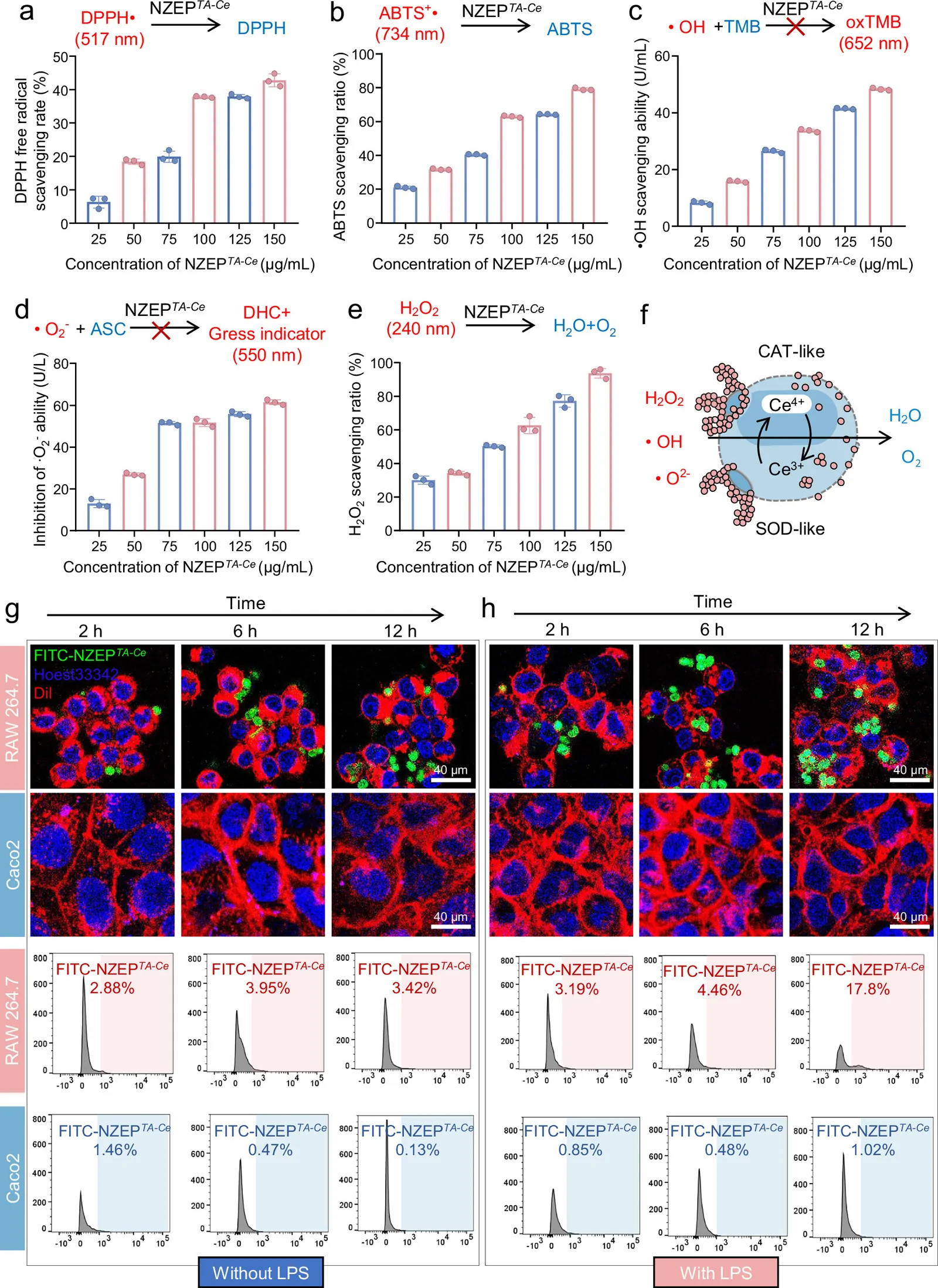
Antioxidant and macrophage cellular targeting capabilities of NZEP^*TA-Ce*^. **a** The ability of the NZEP^*TA-Ce*^ to scavenge DPPH free radical, **b** ABTS free radical, **c** hydroxyl free radical, **d** superoxide anion, **e** hydrogen peroxide. **f** Conceptual diagram of SOD-like activity and CAT-like activity of NZEP^*TA-Ce*^. Representative image and flow cytometry of NZEP^*TA-Ce*^ being taken up by RAW264.7 and Caco2 cells at different time points **g** without or **h** with LPS. In **a**–**e**, data are presented as means ± SD (*n* = 3 independent samples). In **g**, **h**, data are from three independent experiments.

The prominent characteristic of MPN-Ce as the exoskeleton of YGPs lies in its ability to confer potent capabilities to yeast-derived polysaccharides in combating oxidative stress environments. To verify the antioxidant capacity of NZEP, we selected NZEP^*TA-Ce*^ with the highest exoskeletonization degree for the experiment. It was utilized in classical 1,1-diphenyl-2-picrylhydrazyl (DPPH) and 2,2′-azino-bis(3-ethylbenzothiazoline-6-sulfonate) (ABTS) radical scavenging assays. As depicted in Fig. 3a, b, treatment with 150 μg/mL NZEP^*TA-Ce*^ resulted in the removal of approximately 40% of DPPH radicals and 80% of ABTS radicals. Furthermore, selecting three representative biologically relevant reactive oxygen species (ROS), H_2_O_2_, ·O_2_^−^, and ·OH, to investigate the ROS scavenging activity of NZEP^*TA-Ce*^. The NZEP^*TA-Ce*^ demonstrates significant ROS scavenging activity, exhibiting concentration-dependent behavior. Approximately 50% of ·OH were eliminated by 150 μg/mL of NZEP^*TA-Ce*^ (Fig. 3c). Treatment with 150 μg/mL of the NZEP^*TA-Ce*^ resulted in the decomposition of approximately 60% of ·O_2_^−^, demonstrating the SOD-like activity of NZEP^*TA-Ce*^ (Fig. 3d). Moreover, at a concentration of 150 μg/mL, close to 90% of H_2_O_2_ was effectively cleared indicating that the NZEP^*TA-Ce*^ also possesses CAT-like activity (Fig. 3e). The experimental results above confirm that the TA-Ce as the exoskeleton on the surface of NZEP^*TA-Ce*^ exhibits potent antioxidant capabilities, capable of eliminating various ROS through the change in cerium oxidation states (Fig. 3f).

It is widely recognized that the *β*-1,3-D-glucan on the surface of YGPs is classified as a pathogen-associated molecular pattern, capable of being recognized by pathogen recognition receptors on host immune cells, such as Dectin-1, complement receptor 3 (CR3), and TLR2, thereby targeting phagocytes like macrophages. We hypothesize that the NZEP^*TA-Ce*^ also possesses macrophage-targeting capabilities. The biocompatibility of the NZEP^*TA-Ce*^ with cells was first evaluated. At concentrations below 150 μg/mL, the NZEP^*TA-Ce*^ had minimal impact on cell viability. Furthermore, the NZEP^*TA-Ce*^ and its components showed no significant toxicity to Mouse mononuclear macrophage leukemia (RAW 264.7) cells and human colorectal adenocarcinoma (Caco2) cells at this concentration (Supplementary Fig. 11). To investigate the NZEP^*TA-Ce*^’s high specificity and affinity for macrophages, FITC fluorescent probes were labeled on the NZEP^*TA-Ce*^, and the NZEP^*TA-Ce*^ uptake assays were conducted in the RAW 264.7 macrophage and Caco2 cell line. NZEP^*TA-Ce*^ were internalized into the cytoplasm of RAW 264.7 cells, while almost no internalization of NZEP^*TA-Ce*^ was observed in Caco2 cells within 12 h (Fig. 3g). It is noteworthy that, to further simulate the cellular status under inflammatory conditions, the RAW 264.7 and Caco2 cells were stimulated with LPS, followed by another round of NZEP^*TA-Ce*^ cellular uptake experiments. The experimental results revealed that after LPS stimulation, RAW 264.7 cells underwent polarization, leading to a significant enhancement in the internalization efficiency of NZEP^TA-Ce^. By co-localization analysis experiments, it was further verified that NZEP^*TA-Ce*^ was effectively internalized into the cell interior rather than simply attached to the cell membrane surface (Supplementary Figs. 12 and 13). In contrast, the Caco2 cells still could not uptake or internalize the NZEP^*TA-Ce*^ (Fig. 3h). To further investigate the targeting mechanism of NZEP^*TA-Ce*^ for macrophages, we first confirmed the expression of Dectin-1, TLR2 and CR3 on the membrane of RAW264.7 cells (Supplementary Fig. 14). Receptor neutralization with specific antibodies significantly inhibited NZEP^*TA-Ce*^ uptake, reducing internalization from ~4.24% to ~0.15%, with a more pronounced effect in LPS-stimulated cells (Supplementary Fig. 15). These results demonstrate that NZEP^*TA-Ce*^ was primarily internalized by macrophages through receptor-mediated endocytosis involving Dectin-1, CR3, and TLR2. The above results demonstrate that NZEP^*TA-Ce*^’s robust antioxidant properties and its selective targeting of macrophages at the site of inflammation could potentially provide a targeted therapeutic approach for intestinal inflammation.

### Antioxidant and anti-inflammatory properties of NZEP^*TA-Ce*^ in vitro

**Fig. 4:**
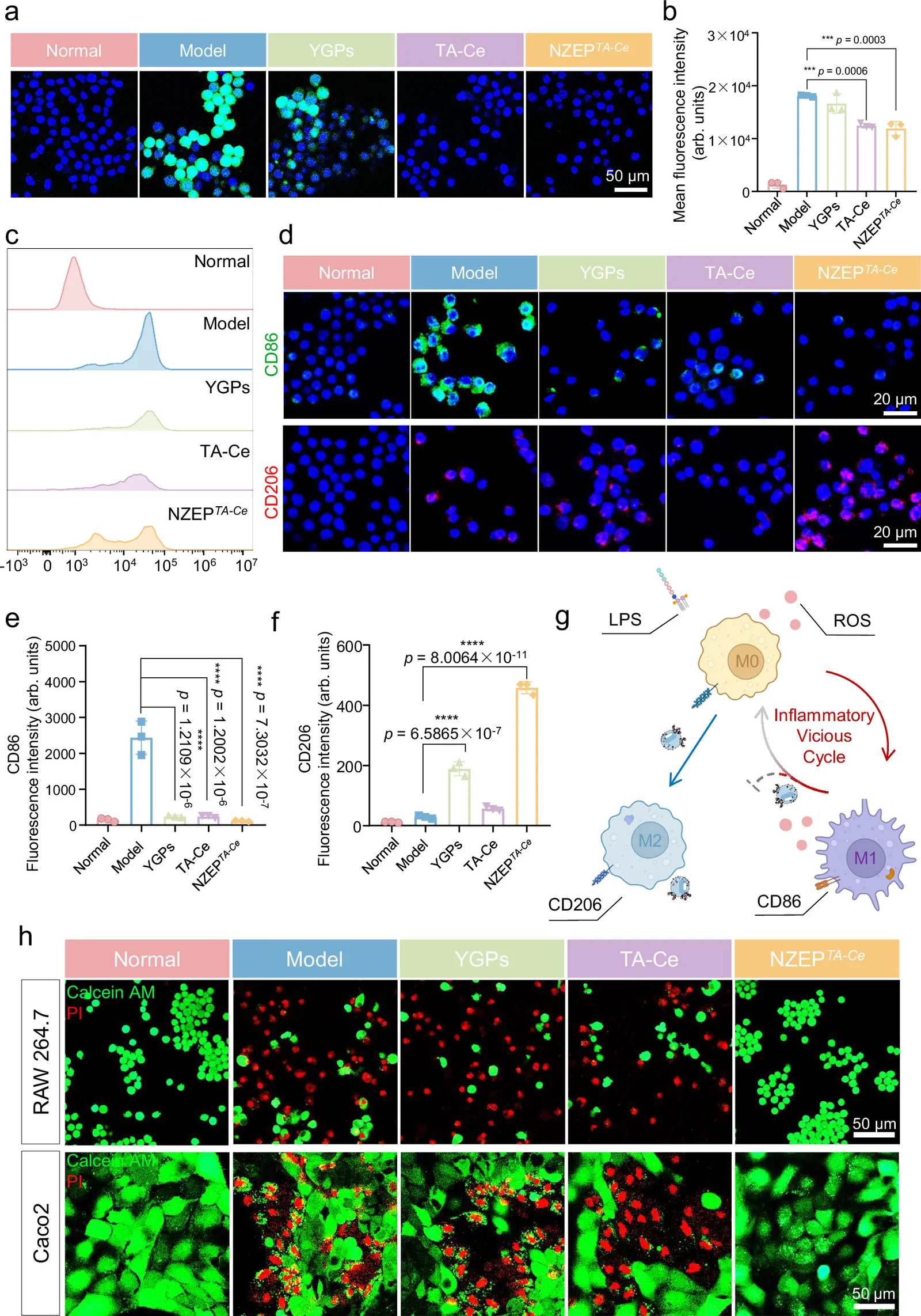
Antioxidant and anti-inflammatory properties of NZEP^*TA-Ce*^. **a** Representative image of DCFH-DA fluorescence in RAW264.7 and **b** statistical data (*n* = 3 independent samples). **c** Representative flow cytometry of DCFH-DA fluorescence in RAW264.7. **d** Representative immunofluorescence staining for CD86 and CD206 of RAW264.7, and (**e**, **f**) statistical data (*n* = 3 independent samples). **g** Schematic diagram of the mechanism of NZEP^*TA-Ce*^ to protect cells from oxidative stress and inflammatory stimulation (created in BioRender. Zhang, F. (2026) https://BioRender.com/i30sapn). **h** Representative image of live/dead cells staining in RAW264.7 and Caco2 cells. In **a**, **d**, **h**, data are from three independent experiments; In **b**, **e** and **f**, statistical significance was analyzed using one-way ANOVA followed by Tukey’s posttest. Unless otherwise stated, data are presented as means ± SD. *p < 0.05, **p < 0.01, ***p < 0.001, and ****p < 0.0001.

Excessive ROS-induced intestinal oxidative stress is considered a major factor in the onset and progression of IBD^32^. Therefore, we first investigated the antioxidant activity of NZEP^*TA-Ce*^ on immune cells under oxidative stress conditions. tert-Butyl Hydroperoxide (tBHP) is one of the most important biologically relevant ROS due to its strong membrane permeability and longer half-life compared to O_2_^−^ and ·OH^33^. Hence, the co-cultivation of tBHP with RAW 264.7 was utilized to mimic the physiological environment of IBD, while assessing the intracellular oxidative stress status through DCFH-DA (a probe that fluoresces green in the presence of ROS) analysis. RAW 264.7 treated with tBHP displayed intense green fluorescence within the cells, indicative of elevated intracellular oxidative stress levels (Fig. 4a). Subsequent treatment with YGPs resulted in a reduction of green fluorescence in certain cells. Conversely, treatment with TA-Ce and NZEP^*TA-Ce*^ led to a notable absence of green fluorescence within the cells (Fig. 4b). Further quantitative analysis of intracellular oxidative stress levels was conducted through flow cytometry, revealing the mild mitigating effect of YGPs on reducing intracellular oxidative stress levels. TA-Ce and NZEP^*TA-Ce*^ were able to significantly alleviate intracellular oxidative stress levels (Fig. 4c).

Intense inflammatory disruption is a prominent feature of IBD^34,35^. Yeast-derived polysaccharides possess immunomodulatory functions and are widely used as therapeutic agents to either activate or suppress the immune system^15^. In particular, the treatment of macrophages with LPS and IFN-γ allowed for a detailed investigation into the influence of the NZEP^*TA-Ce*^ on the polarization process of macrophages. Confocal laser scanning microscopy images and flow cytometry analysis (Fig. 4d–f and Supplementary Fig. 16a) revealed that LPS + IFN-γ-induced macrophages showed an upregulation of the M1 macrophage marker (CD86) and a downregulation of the M2 macrophage marker (CD206), with YGPs showing a slight reversal of these alterations. As TA-Ce lacks the capacity to modulate immune cell polarization, it primarily reduced the expression of M1 markers, with a less pronounced increase in M2 markers. Remarkably, post- NZEP^*TA-Ce*^ treatment, there was a significant reduction in the expression of the M1 macrophage marker (CD86) and a marked increase in the expression of the M2 macrophage marker (CD206). Additionally, Real-time reverse transcription polymerase chain reaction (RT-qPCR) analysis revealed a significant upregulation of pro-inflammatory mRNA expressions (TNF-α, IL-1β, iNOS, and CD86) after LPS + IFN-γ stimulation, while treatment with the NZEP^*TA-Ce*^ markedly downregulated their mRNA expression level (Supplementary Fig. 16b). Compared to YGPs, NZEP^*TA-Ce*^ demonstrates significantly enhanced anti-inflammatory effects, possibly attributed to the dual functionality of NZEP^*TA-Ce*^, which includes the ROS scavenging capability of the nanozyme-exoskeleton and the immunomodulatory role of YGPs. The synergistic effect of these properties in NZEP^*TA-Ce*^ allows for the disruption of the vicious cycle of inflammation, presenting potent anti-inflammatory efficacy and showing significant potential to restore the homeostatic balance of intestinal inflammation (Fig. 4g).

To further simulate the physiological environment of immune cells and Caco2 cell under conditions of disrupted intestinal inflammatory homeostasis, we established an in vitro model by simultaneously adding LPS, IFN-γ, and tBHP to the culture medium, and utilized live/dead staining to assess the NZEP^*TA-Ce*^’s synergistic protective effect. The environment of inflammation severely impairs the viability of RAW 264.7 and Caco2 cell, with a significant increase in the red fluorescence of PI compared to the normal culture environment (Fig. 4h and Supplementary Fig. 17). While the individual effects of immunomodulatory or antioxidant activity may slightly alleviate the cytotoxicity induced by the vicious cycle of inflammation, the NZEP^*TA-Ce*^ completely interrupts the process of the vicious cycle of inflammation, significantly enhancing cell viability. Cell viability is nearly 100%, with no apparent red fluorescence of PI, indicating a pronounced cell-protective effect.

### Potential anti-inflammatory mechanism via RNA-sequencing analysis

**Fig. 5:**
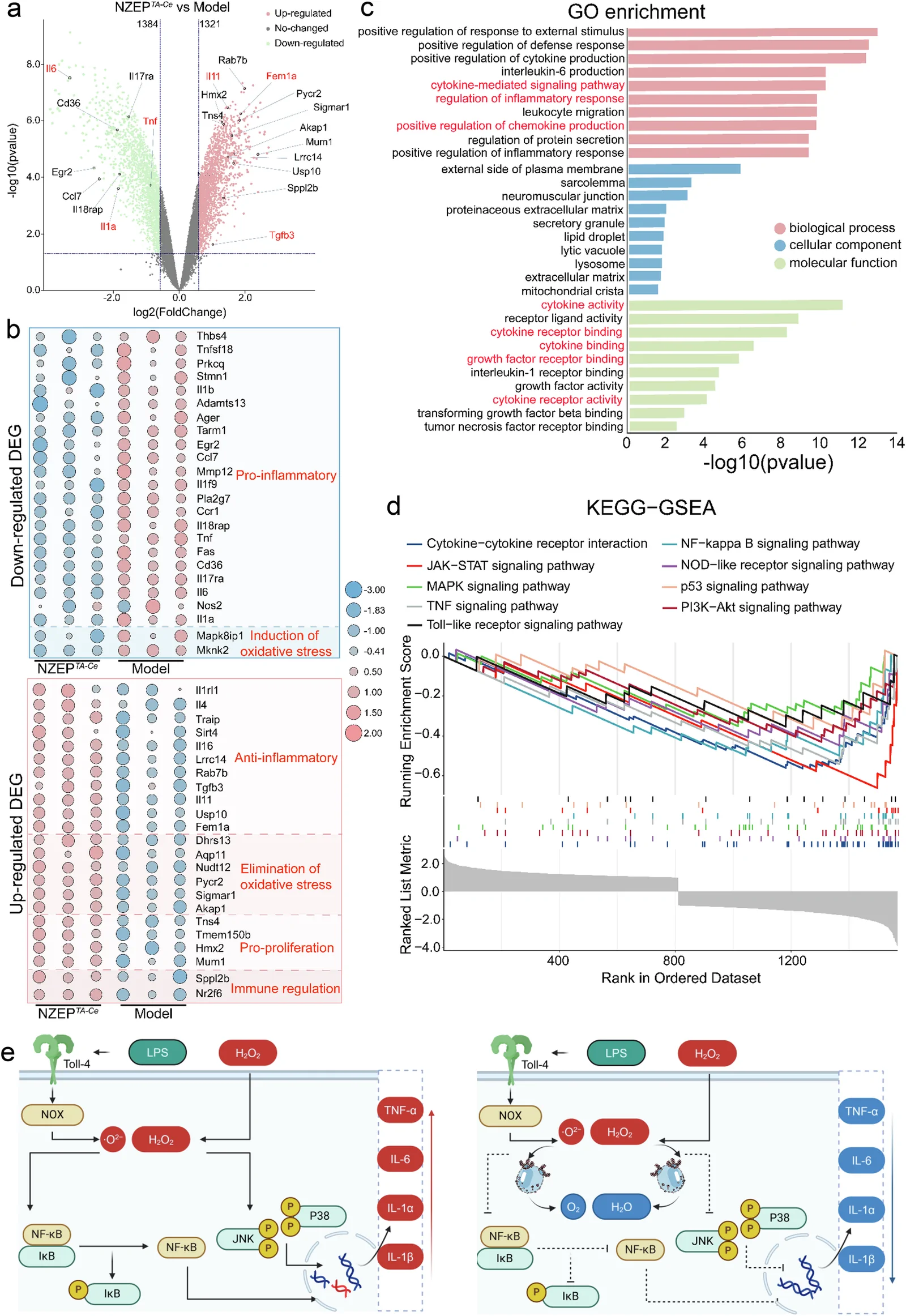
Analysis RNA sequencing results for NZEP^*TA-Ce*^ treatment. **a** Volcano plot of DEGs of the RAW264.7 cell in the NZEP^*TA-Ce*^ group versus the model group. Statistical significance was determined based on adjusted *p*-value < 0.05. **b** Heat map of DEGs in inflammatory response, oxidative stress, cell proliferation, and immune regulation between the NZEP^*TA-Ce*^ and model group. **c** GO enrichment analyses of DEGs, and the x-axis represents -log10(adjusted *p*-value). **d** Significantly enriched GSEA-KEGG pathways. Each pathway was ranked by normalized enrichment scores (NES). **e** Schematic representation of the potential anti-inflammatory mechanism of the NZEP^*TA-Ce*^ (created in BioRender. Zhang, F. (2026) https://BioRender.com/yp1ymk5). RNA-seq was performed with *n* = 3 biologically independent samples per group.

The NZEP^*TA-Ce*^’s strong antioxidant and anti-inflammatory effects in the experiment prompted us to explore its potential mechanisms further. RAW 264.7 cells were initially stimulated with LPS, IFN-γ, and tBHP (model group) and then treated with NZEP^*TA-Ce*^ (NZEP^*TA-Ce*^ group). The differentially expressed genes (DEGs) between the two groups were then identified and analyzed. RAW 264.7 cells treated with NZEP^*TA-Ce*^ exhibited a significant increase in 1321 DEGs compared to the model group, including *Il-11* and *Tgfb3*, which encode anti-inflammatory cytokines, and *Fem1a*, linked to PGE2-EP4-mediated macrophage inflammation suppression^36,37^. Moreover, 1384 DEGs were significantly downregulated, including inflammatory markers like *Il6*, *Il1a*, and *Tnf* ^38–40^ (Fig. 5a). The heat map of DEGs revealed that the upregulated DEGs were primarily associated with inhibiting inflammatory responses, reducing cellular oxidative stress, promoting cell proliferation, and immune regulation. In contrast, the downregulated DEGs were mainly involved in promoting inflammatory responses and inducing cellular oxidative stress (Fig. 5b). We conducted gene ontology (GO) enrichment analysis on DEGs using biological processes, cellular components, and molecular functions. The analysis showed that NZEP^*TA-Ce*^ significantly affected inflammation-related processes, particularly cytokine signaling pathways and chemokine production regulation, and modulated cytokine activity and their receptors, indicating a wide impact on immune functions (Fig. 5c). Grouping the differentially expressed genes for KEGG enrichment analysis revealed potential molecular mechanisms, including cytokine interactions, IBD, and the intestinal immune network for IgA production (Supplementary Fig. 18). This indicates that NZEP^*TA-Ce*^ may have therapeutic potential for IBD and related inflammatory diseases. We performed gene set enrichment analysis (GSEA) to examine the enrichment of differentially expressed genes in biological pathways. This revealed several significantly enriched pathways, mainly associated with cellular inflammation and oxidative stress, including the Toll-like receptor (TLRs, NES = − 1.95), mitogen-activated protein kinase (MAPK, NES = − 1.61), and nuclear factor-κB (NF-κB, NES = − 0.50) signaling pathways. These findings indicate that NZEP^*TA-Ce*^ plays a crucial role in inhibiting cellular inflammation and maintaining oxidative stress homeostasis (Fig. 5d). Finally, we validated the mRNA and protein expression of key differentially expressed genes (*Il-1a*, *Il-1b*, *Il-6*, *Nos2*, *Tnfa*, *Il-4*, *Il-11*, *Tgfb3*, *Fem1a*, and *Akap1*) through RT-qPCR and cellular immunofluorescence experiments (Supplementary Fig. 19). The results were consistent with the RNA-sequencing data. Based on the comprehensive results from the above experiments, the potent anti-inflammatory mechanisms of the NZEP^*TA-Ce*^ can be hypothesized as follows (Fig. 5e). The NZEP^*TA-Ce*^ scavenges both exogenous and endogenous ROS (produced via the Toll-like receptor signaling pathway), blocking the MAPK and NF-κB-mediated inflammatory signaling pathways. This disruption breaks the vicious cycle of inflammation, inhibits the polarization of M1 phenotype immune cells, reduces the secretion of pro-inflammatory cytokines, and alleviates the inflammatory response.

### Retention and targeting processes of NZEP^*TA-Ce*^ in colonic inflammation

**Fig. 6:**
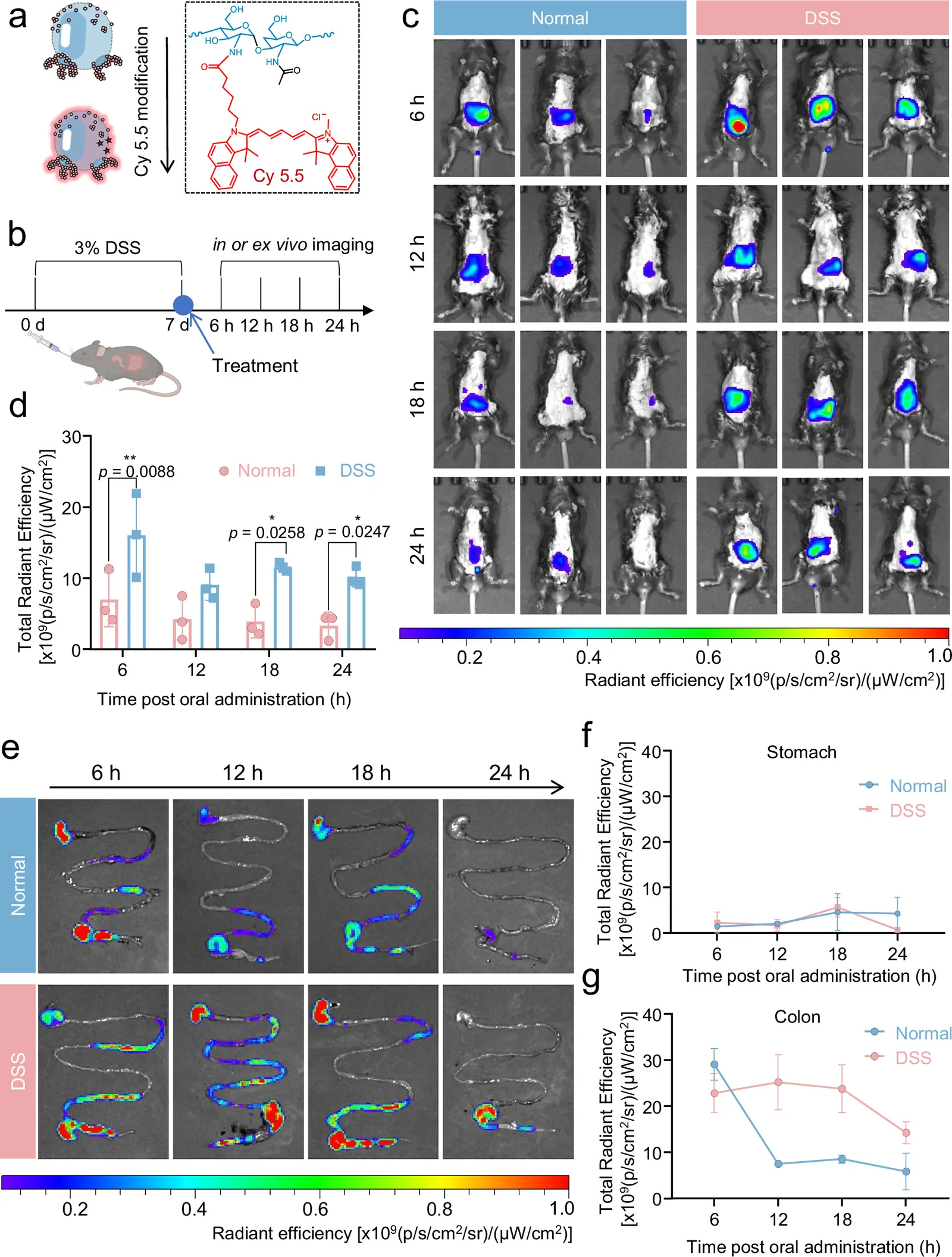
The targeting and retention abilities of NZEP^*TA-Ce*^ to the inflamed colon. **a** Schematic diagram of the preparation process of NZEP^*TA-Ce*^-Cy5.5. **b** Schematic diagram of the experimental process (created in BioRender. Zhang, F. (2026) https://BioRender.com/mn1wa3r). **c** In vivo fluorescence images of normal and IBD mice at different time points after oral administration of NZEP^*TA-Ce*^-Cy5.5 (*n* = 3 mice). **d** Total radiant efficiency of normal and IBD mice at different time points. **e** Fluorescence images of mice gastrointestinal tissues at different time points after oral administration of NZEP^*TA-Ce*^-Cy5.5 (*n* = 3 mice). **f** Total radiant efficiency of mice stomach at different time points (*n* = 3 mice). **g** Total radiant efficiency of mice colon at different time points (*n* = 3 mice). In **c**, **e**, data are from three independent experiments. In **d**, **f** and **g**, statistical significance was analyzed using two-way ANOVA followed by Tukey’s posttest. Data are presented as means ± SD. *p < 0.05, **p < 0.01, ***p < 0.001, and ****p < 0.0001.

Prolonged retention and targeting at the site of colonic inflammation are advantageous for enhancing the effectiveness of oral therapy and reducing potential toxic side effects^41^. Hence, we investigated the distribution and retention of the NZEP^*TA-Ce*^ in both DSS-induced IBD and normal C57BL/6 mice models. NZEP^*TA-Ce*^ modified with Cyanine 5.5 (Cy5.5) were orally administered in DSS- induced IBD and normal mice (Fig. 6a). The fluorescence intensity of NZEP^*TA-Ce*^ in the abdominal cavities of different mice was monitored at 6, 12, 18, and 24 h using an in vivo imaging system (Fig. 6b). The fluorescence intensity of NZEP^*TA-Ce*^ in the abdominal cavity of DSS induced IBD mice at 6, 12, 18, and 24 h after oral administration was 2.28, 2.13, 2.96, and 3.0 times higher than that of the normal group, indicating that NZEP^*TA-Ce*^ has a prolonged retention effect in the inflamed intestine (Fig. 6c, d). To further elucidate the targeted and retention effects of NZEP^*TA-Ce*^ on the inflamed colonic region, the aforementioned experimental procedure was repeated. Following oral administration of NZEP^*TA-Ce*^ at 6, 12, 18, and 24 h, the gastrointestinal tract was excised for ex vivo fluorescence imaging. Subsequently, the fluorescence distribution of collected ex vivo tissues was analyzed and statistically evaluated (Fig. 6e). During the experimental observation period, the distribution of NZEP^*TA-Ce*^ in the stomach remained consistent between the normal group and the DSS group, showing no significant differences (Fig. 6f). Following oral administration at 12 and 18 h, the fluorescence intensity in the colon of the DSS group was 3.36 and 2.78 times higher than that in the normal group, respectively. Even at 24 h, the colon of the DSS group continued to exhibit strong fluorescence emission (Fig. 6g). Fluorescence staining of colon tissue sections from the DSS group after treated with NZEP^*TA-Ce*^ demonstrated the presence of NZEP^*TA-Ce*^ accumulation on the surface of the colonic region (Supplementary Fig. 20a). These results indicate that NZEP^*TA-Ce*^ can more effectively target and accumulate in the inflamed colonic regions. In addition, no strong fluorescence accumulation was observed in the hearts, livers, spleens, lungs, and kidneys of the two groups of mice during the treatment process (Supplementary Fig. 20b).

Before assessing the therapeutic efficacy of NZEP^*TA-Ce*^ for IBD, we evaluated the in vivo biocompatibility through oral administration. No significant weight loss or organ differences were noted compared to the control group during the treatment (Supplementary Fig. 21). Key blood parameters, including white blood cells, red blood cells (RBC), monocytes (MON), platelets (PLT), and others, remained within normal ranges. Liver function indicators, including alanine aminotransferase (ALT), albumin (ALB), aspartate aminotransferase (AST), and total protein (TP), showed no significant changes (Supplementary Fig. 22). Furthermore, to investigate any potential damage to major organs during oral treatment with the NZEP^*TA-Ce*^, ex vivo organs were subjected to hematoxylin and eosin (H&E) staining at the end of the experiment. No significant pathological changes were observed in the histological analysis (Supplementary Fig. 23), and NZEP^*TA-Ce*^ exhibited negligible hemolysis of RBC up to a concentration of 1200 μg/mL (Supplementary Fig. 24). These experimental results suggest that NZEP^*TA-Ce*^ does not cause systemic toxicity when taken orally.

### Therapeutic effect of NZEP^*TA-Ce*^ in DSS-induced IBD mice model

**Fig. 7:**
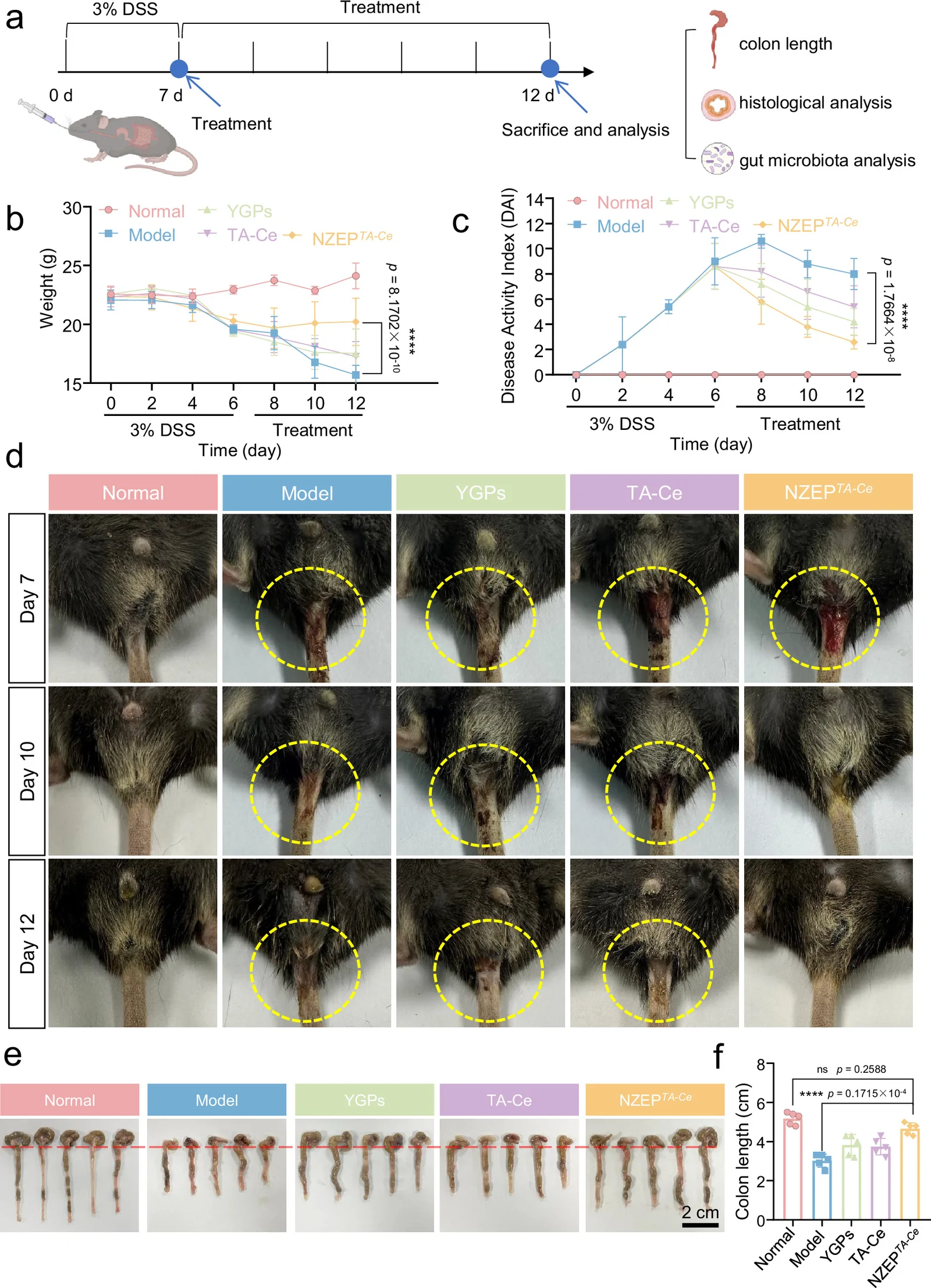
Therapeutic efficacy of NZEP^*TA-Ce*^ against DSS-induced mice IBD model. **a** Schematic of the treatment plan. The mice were administered with 3% DSS from days 0 to 7 to induce the IBD. Afterward, different formulations were given to the mice for 5 days to assess their therapeutic efficacy (created in BioRender. Zhang, F. (2026) https://BioRender.com/ihz4sd0). **b** The weight changes of IBD-bearing mice in different treatment groups (*n* = 5 mice). **c** Disease activity index (DAI) score of IBD model mice during the experiment (*n* = 5 mice). **d** Representative photographs of the anus during treatment of different groups of IBD mice (*n* = 5 mice). The yellow dashed circles indicate anal blood residue in mice. **e** Colon images and **f** quantified colon lengths in different treatment groups (*n* = 5 mice). Three independent experiments were performed. In **b**, **c**, two-way ANOVA followed by Tukey’s posttest. In **f**, one-way ANOVA followed by Tukey’s posttest. Data are presented as means ± SD. *p < 0.05, **p < 0.01, ***p < 0.001, and ****p < 0.0001.

The remarkable anti-inflammatory effects of NZEP^*TA-Ce*^ in vitro, along with its specific targeting of inflamed colonic regions, prompted us to investigate its therapeutic potential in restoring intestinal inflammatory balance in a DSS-induced IBD model in C57BL/6 mice. The mice received 3% DSS in drinking water orally for 7 days consecutively to induce IBD. Following this, they were randomly assigned to five groups: normal, model, YGPs, TA-Ce, and NZEP^*TA-Ce*^. The model group, acting as the positive control, remained untreated, while the other groups received the respective treatments via oral gavage for 5 days (Fig. 7a). Throughout the treatment, body weight monitoring showed that mice treated with YGPs or TA-Ce experienced less weight loss compared to the model group, indicating a mitigating effect of YGPs or TA-Ce on IBD. Mice treated with NZEP^*TA-Ce*^ not only showed greater body weight recovery compared to other groups but also displayed a progressive increase during treatment (Fig. 7b). Compared to the model group, NZEP^*TA-Ce*^ significantly alleviated IBD symptoms, as reflected by the disease activity index (DAI) and anal blood residue (Fig. 7c, d).

**Fig. 8:**
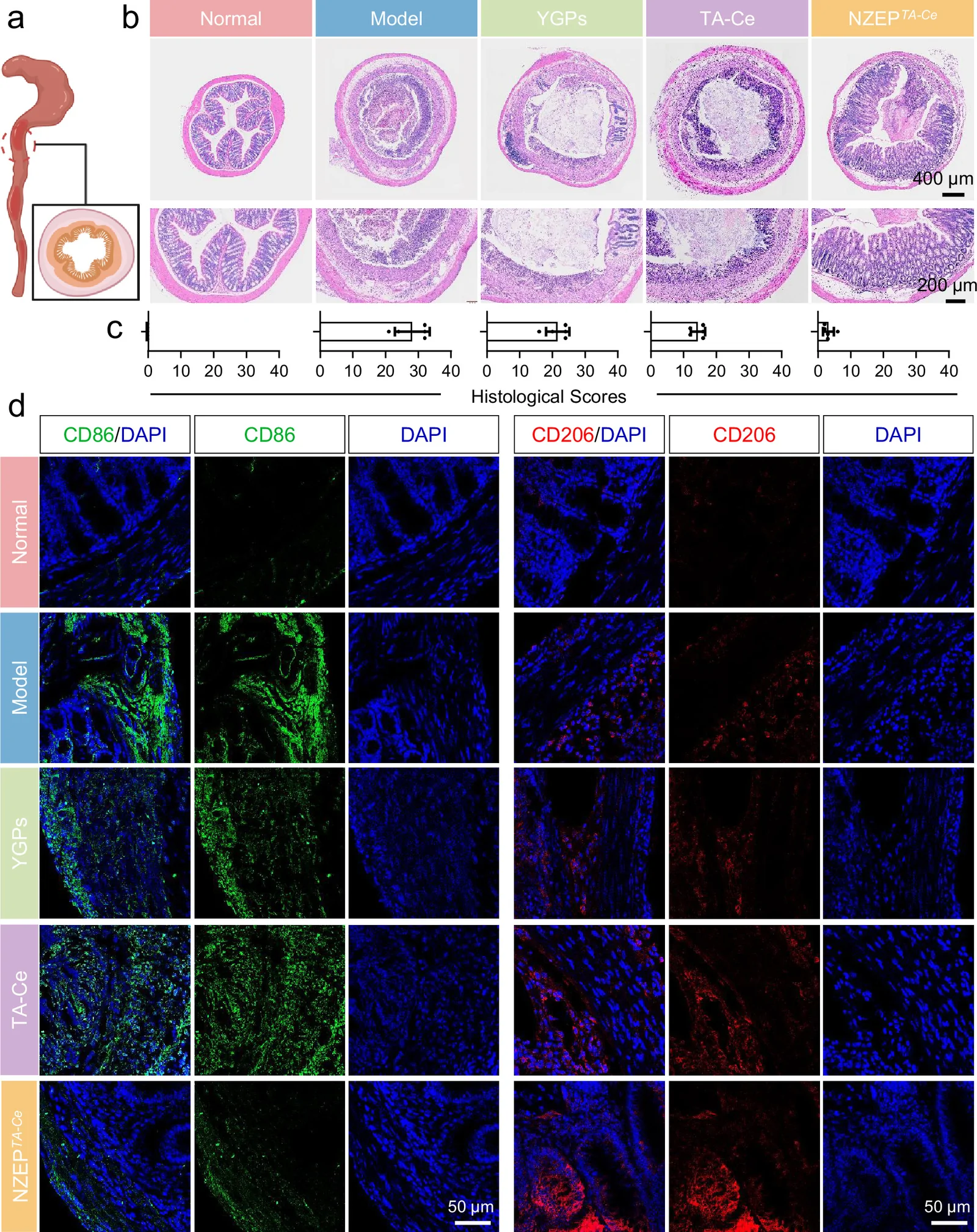
The NZEP^*TA-Ce*^ reverses the imbalance of intestinal inflammatory homeostasis. **a** Schematic illustration of the location of the mouse colon tissue sampling (created in BioRender. Zhang, F. (2026) https://BioRender.com/5in4bvj). **b** Representative H&E staining colon tissue images and **c** histopathology scores of colon tissues to evaluate colon damage (*n* = 5 mice), and data are presented as means ± SD. **d** Representative immunofluorescence staining for CD86 and CD206 of colonic tissue. In **b**, **d**, data are from three independent experiments.

In IBD, inflammation and extracellular matrix buildup are usually limited to the colonic mucosa and submucosa, causing the mucosal muscle layer to thicken, potentially shortening or stiffening the colon and impairing motility^42^. Colon length in the model group decreased by 41.6% compared to the normal group, while mice treated with NZEP^*TA-Ce*^ showed only a 10% reduction (Fig. 7e, f, and Supplementary Fig. 25). Transverse colon sections were prepared and examined for histopathological changes using H&E staining (Fig. 8a). The model group showed severe colonic crypt disruption, high immune cell infiltration, and significant epithelial damage. In contrast, NZEP^*TA-Ce*^ treated mice displayed mostly normal colon microstructure and reduced inflammatory cell infiltration (Fig. 8b). The quantitative evaluation of histopathological scores confirmed that NZEP^*TA-Ce*^ effectively reduced inflammation at colonic lesions. The NZEP^*TA-Ce*^ group had significantly lower histological scores than the model group, indicating reduced inflammation and crypt damage (Fig. 8c). To further characterize inflammatory and barrier-associated changes in colonic tissues after treatment, macrophage-associated markers, inflammatory and oxidative stress indices, and tight junction proteins were examined. Quantitative analysis showed numerically lower CD86 fluorescence intensity and higher CD206 fluorescence intensity in the NZEP^*TA-Ce*^ group than in the model group (Fig. 8d and Supplementary Fig. 26). Similarly, MPO activity and MDA content were numerically lower in the NZEP^*TA-Ce*^ group than in the model group, whereas the fluorescence intensities of ZO-1 and occludin were numerically higher. (Supplementary Figs. 27 and 28). Furthermore, no accumulation of Ce ions was detected in the blood of mice, indicating that NZEP^*TA-Ce*^ did not lead to Ce ion retention in the body at the dosage used for IBD treatment (Supplementary Table 5).

### Effects of NZEP^*TA-Ce*^ on gut microbiota and microbial metabolites

**Fig. 9:**
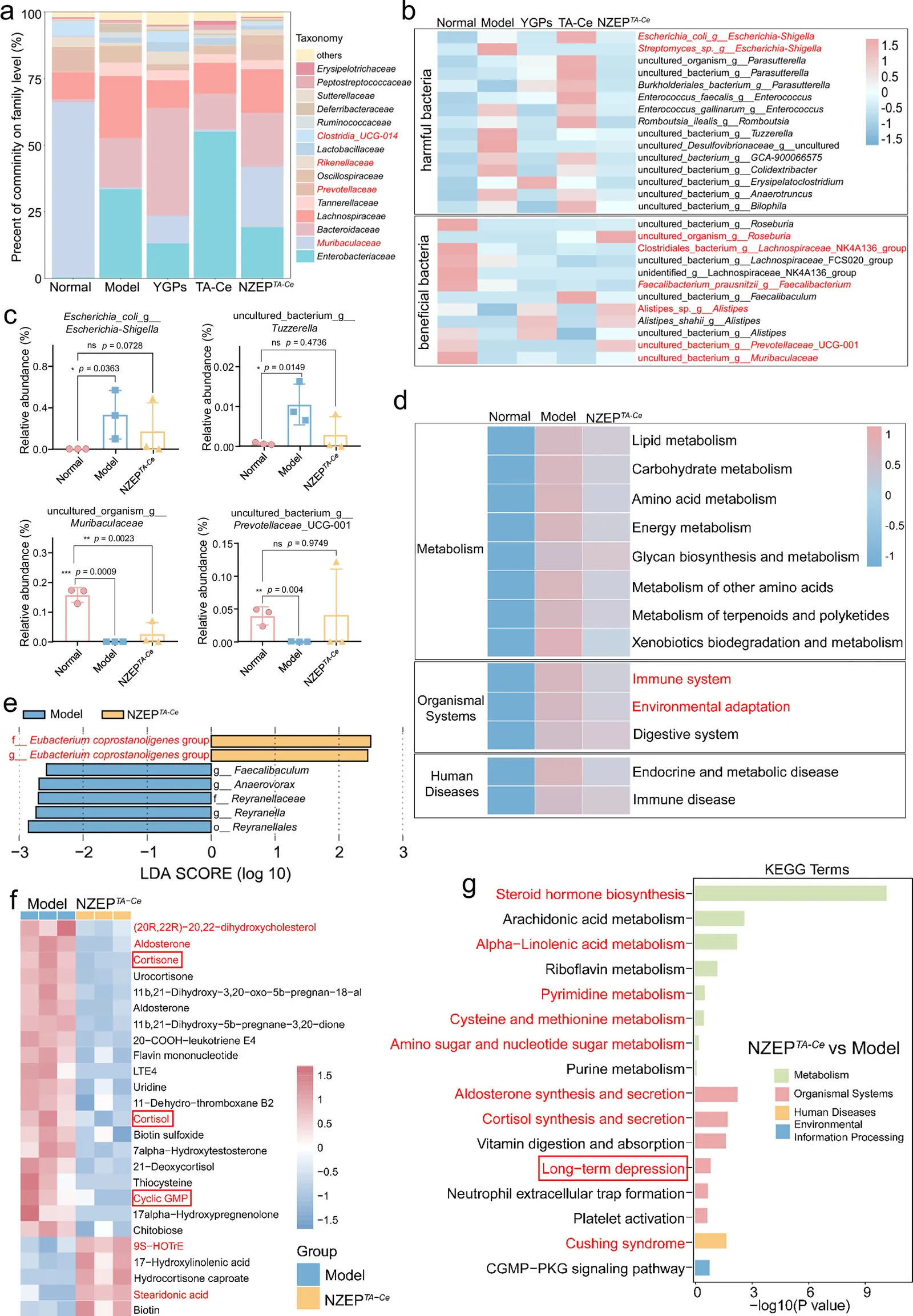
Effects of NZEP^*TA-Ce*^ on the various intestinal flora and metabolites in DSS-induced mouse IBD model. **a** Histogram of the relative abundances of different species on family level. **b** Heatmap of the relative abundances of the various microbial components analyzed at the species level. Each column represents one group, *n* = 3 mice per group. **c** Relative abundances of the microbiota with significant alterations, *n* = 3 mice per group, and data are presented as means ± SD. **d** Heat map of the functional abundance clustering in the different mouse groups. Each column represents one group. **e** Linear discriminant analysis (LDA) identified microbiota that were significantly different in different mouse groups. **f** Heat map of the total differential metabolite clustering in the model versus NZEP^*TA-Ce*^ group. Each column represents one mouse; *n* = 3 mice per group. The red rectangles highlight depression-related metabolites (cortisone, cortisol, and GMP). **g** KEGG enrichment plot of the differential metabolites in the model and NZEP^*TA-Ce*^ group. The red rectangle highlights the long-term depression pathway. In **c**, one-way ANOVA followed by Tukey’s posttest. Unless otherwise stated, data are presented as means ± SD. *p < 0.05, **p < 0.01, ***p < 0.001, and ****p < 0.0001.

Gut microbiome imbalance is key in IBD development. Recent studies reveal that gut microbiota influences the immune, metabolic, and nervous systems via the “gut-brain axis.”^11,43^ To study NZEP^*TA-Ce*^’s effects on gut microbiota and metabolism in IBD-treated mice, we collected fecal samples from healthy and IBD mice treated with YGPs, TA-Ce, or NZEP^*TA-Ce*^ for 16S ribosomal RNA (rRNA) gene sequencing. The α-diversity analysis showed a significant reduction in microbiome diversity and abundance in the feces of DSS-induced IBD mice (Supplementary Fig. 29a, b). In contrast, the NZEP^*TA-Ce*^ group exhibited gut microbiota abundance and diversity comparable to the normal group, suggesting that the treatment may have restored these metrics in the mice. β-diversity shows species quantity and distribution differences within communities. The unweighted UniFrac distance matrix heatmap revealed significant differences between the NZEP^*TA-Ce*^ group, healthy mice, and the model group (Supplementary Fig. 29c). Family-level analysis showed that the NZEP^*TA-Ce*^ group had more beneficial bacteria like *Muribaculaceae*, *Prevotellaceae*, *Rikenellaceae*, and *Clostridia-*UCG-014. Clinical data indicate that Muribaculaceae exhibits a higher relative abundance in healthy individuals compared to those with IBD^44^. Furthermore, Prevotellaceae is recognized for its production of short-chain fatty acids (SCFAs)^45^. In contrast, the NZEP^*TA-Ce*^ group demonstrated a significant reduction in the abundance of pathogenic bacteria, such as *Enterobacteriaceae* and *Tannerellaceae*, which are associated with the exacerbation of IBD (Fig. 9a). Cluster analysis conducted at the species level indicated that the NZEP^*TA-Ce*^ treatment significantly increased the abundance of anti-inflammatory bacteria, including *Faecalibacterium prausnitzii*_(g_*Faecalibacterium*)  and *Alistipes*_*sp*._(g_*Alistipes*)^46,47^. The treatment increased the abundance of SCFA-synthesizing bacteria, including *uncultured bacterium g*_*Muribaculaceae*, *Clostridiales bacterium g*_*Lachnospiraceae* NK4A136 group, uncultured *bacterium g*_*Prevotellaceae* UCG-001, and uncultured bacterium *g*_*Roseburia*, while significantly reducing harmful bacteria like *Escherichia coli* (*g*_*Escherichia-Shigella*) and *Streptomyces* sp. (*g_Escherichia-Shigella*) (Fig. 9b, c). NZEP^*TA-Ce*^ efficiently eliminates ROS from IBD lesions, while mannose encourages the growth of probiotics, potentially explaining the increased relative abundance of probiotics. The KEGG Orthology (KO) heatmap indicates that the NZEP^*TA-Ce*^ group resembled the healthy group more than the model group, particularly in metabolic functions, organismal systems, and human diseases. At the second KEGG level, NZEP^*TA-Ce*^ improved immune functions and environmental adaptability, as well as enhanced lipid, carbohydrate, and amino acid metabolism in IBD mice (Fig. 9d). Experimental results show that NZEP^*TA-Ce*^ treatment reverses gut microbiota dysbiosis, aiding gut immunity regulation and intestinal tissue repair.

The Linear discriminant analysis Effect Size (LEfSe) analysis showed a significant increase in *Eubacterium* abundance in the NZEP^*TA-Ce*^ group compared to the model group. This bacterium’s depletion is linked to major depressive disorder (MDD)^48,49^. Changes in gut microbiota composition affect the microbial metabolome, significantly influencing the development of MDD^13^. We analyzed metabolite changes in different mouse groups using untargeted metabolomics. Principal component analysis (PCA) revealed significant differences in metabolite distributions among the normal, model, and NZEP^*TA-Ce*^ groups (Supplementary Fig. 30a, b). We analyzed metabolite differences among the three groups. The model group showed 1718 upregulated and 1472 downregulated metabolites compared to the normal group. In contrast, the NZEP^*TA-Ce*^ group had 672 upregulated and 679 downregulated metabolites, suggesting its metabolite profile was more similar to healthy mice (Supplementary Fig. 30c). Additionally, the NZEP^*TA-Ce*^ group had 1367 upregulated and 1024 downregulated metabolites compared to the model group (Supplementary Fig. 30d).

We discovered that IBD mice experienced changes in some endogenous metabolite levels after YGPs intervention by comparing the differential metabolites in the YGPs and model groups (Supplementary Fig. 31a). The NZEP^*TA-Ce*^ group exhibited more significant metabolite changes than the YGPs group (Supplementary Fig. 31b). (20R,22R)-20,22-dihydroxycholesterol, a natural metabolite crucial for hormone production^50^, is elevated in DSS-induced IBD mice, aligning with our results. Its expression was reduced in both YGPs and NZEP^*TA-Ce*^-treated groups, with the NZEP^*TA-Ce*^ group showing the most significant decrease compared to the model group. Aldosterone, a pivotal corticosteroid regulating electrolyte and fluid balance, exacerbates IBD by promoting ROS generation within the intestinal microenvironment^51^. Studies have also identified aldosteronism as a characteristic feature in patients with depression^52,53^. In our analysis, the Model group exhibited the highest Aldosterone metabolite levels, whereas the NZEP^*TA-Ce*^ group showed the lowest levels, with YGPs falling in between. 9S-HOTrE, an anti-inflammatory eicosanoid, is reduced in enteritis patients compared to healthy individuals^54^ and has immunomodulating effects, as shown in mice and cell models^55,56^. Treatment in the NZEP^*TA-Ce*^ group significantly increased the levels of 9S-HOTrE in metabolites and played a more pronounced role in increasing them than YGPs. Stearidonic acid, an Omega-3 PUFA, reduces cytokines, adhesion molecules, and reactive oxygen species to help prevent inflammatory disorders like IBD^57,58^. Our data indicate that NZEP^*TA-Ce*^ significantly boosts stearidonic acid levels in IBD mice more effectively than YGPs. Additionally, stearidonic acid protects against neurotoxicity in rat hippocampal cells due to its antiapoptotic, antioxidant, and anti-inflammatory effects^59^. We speculated that the intervention of NZEP^*TA-Ce*^ not only reduces the level of intestinal inflammation but also protects against neuronal damage caused by IBD.

We speculated that YGPs, as prebiotics, may influence endogenous metabolite synthesis and breakdown by stimulating the intestinal immune system. They also enhance metabolite production by inhibiting harmful microorganisms, promoting a healthy gut microbiota. In inflammatory conditions, NZEP^*TA-Ce*^ exhibits anti-inflammatory and ROS-scavenging properties, supporting YGPs’ functions. Consequently, NZEP^*TA-Ce*^ amplifies the above effects.

The clustering heatmap of differential metabolites (Fig. 9f) indicated that cortisone levels were lower in the NZEP^*TA-Ce*^ group than in the model group. Cortisone, a crucial glucocorticoid in rodents that regulates stress, is often used in depression models. The model group exhibited elevated cortisol levels, which are typically high in major depressive disorder (MDD)^60–62^. Furthermore, cyclic GMP, linked to the nitric oxide/cyclic GMP (NO/cGMP) pathway, was significantly downregulated following NZEP^*TA-Ce*^ treatment, highlighting its studied role in MDD^63,64^. The KEGG enrichment maps show that both YGPs and NZEP^*TA-Ce*^ altered metabolite composition and metabolic structure by affecting steroid hormone synthesis and the metabolism of arachidonic and linoleic acids in DSS mice (Fig. 9g and Supplementary Fig. 31c). Furthermore, NZEP^*TA-Ce*^ also impacted metabolites linked to long-term depressive activity in mice. These findings indicate that NZEP^*TA-Ce*^ treatment reduces cortisone, cortisol, and GMP levels in mice, suggesting its potential to alleviate anxiety and depression in IBD.

### Therapeutic effect of NZEP^*TA-Ce*^ in CD4^+^CD45RB^hi^ T cell transfer model

The CD4^+^CD45RB^hi^ T cell transfer model, a well-established chronic IBD model, induces colitis by injecting naïve CD4^+^ T cells into lymphocyte-deficient hosts, closely mimicking T cell-mediated immunopathology and the characteristics of idiopathic IBD in humans. To further evaluate the adaptive immune regulatory effects of NZEP^*TA-Ce*^, we investigated its therapeutic efficacy and impact on gut immune cell populations (Supplementary Fig. 32a). NZEP^*TA-Ce*^ treatment significantly alleviated IBD-associated clinical symptoms, including reduced perianal inflammation, body weight loss, and DAI scores compared to the model group (Supplementary Fig. 32b–d). Additionally, colon length measurements and histopathological analysis revealed attenuated colon shortening and preserved intestinal integrity, as confirmed by H&E staining (Supplementary Fig. 32e–g). A reduced spleen index further suggested potential mitigation of systemic inflammation (Supplementary Fig. 32h–j).

Flow cytometric analysis (Supplementary Figs. 33 and 34a–d) demonstrated that NZEP^*TA-Ce*^ decreased pro-inflammatory Th1/Th17 cells while increasing anti-inflammatory Treg cells in the colonic lamina propria, suggesting enhanced immune tolerance. Furthermore, IL-7 immunofluorescence staining and Elisa revealed a downregulation of pro-inflammatory cytokines (IFN-γ, IL-17, and IL-21) and upregulation of anti-inflammatory cytokines (IL-10, TGF-β, and IL-4) (Supplementary Fig. 34e, f), indicating that NZEP^*TA-Ce*^ restores the balance between pro- and anti-inflammatory immune responses.

All of the above results indicated that NZEP^*TA-Ce*^ effectively ameliorated IBD progression by mitigating clinical symptoms, preserving intestinal integrity, suppressing inflammatory immune responses, and promoting immune homeostasis, underscoring its therapeutic potential for IBD treatment and clinical translation.

### Modulating effects of NZEP^*TA-Ce*^ on depression and anxiety in IBD mice

**Fig. 10:**
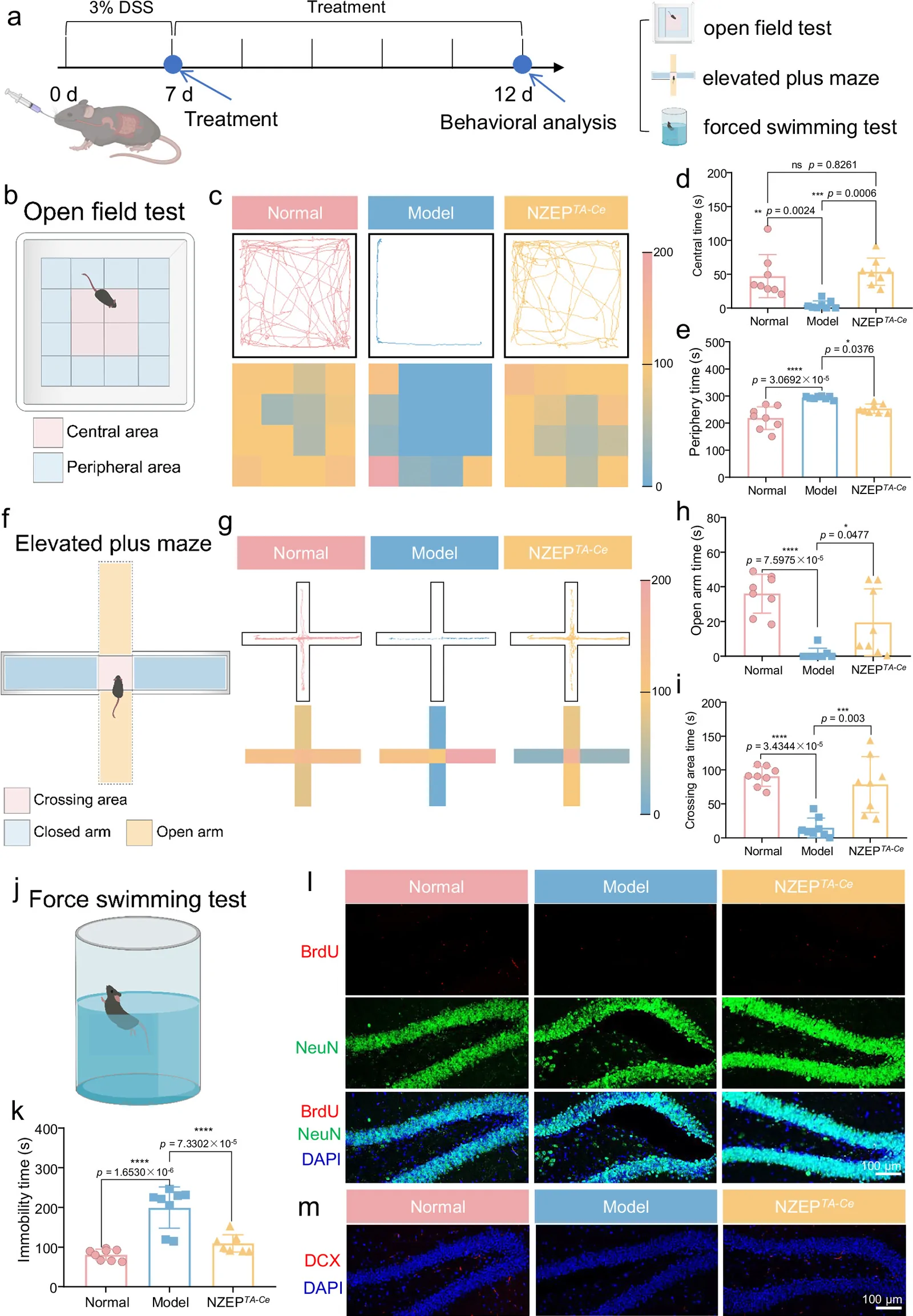
NZEP^*TA-Ce*^ alleviates IBD-related diseases. **a** Schematic illustration of model building and treatment. **b** Schematic diagram of the open field test (OFT). **c** Movement tracking images and heatmaps of mice in the OFT. **d** Time spent in the central area in the OFT (*n* = 8 mice). **e** Time spent in the peripheral area in the OFT (*n* = 8 mice). **f** Schematic diagram of the EPM. **g** Movement tracking images and heatmaps of mice in the EPM test. **h** Time spent in the open arm in the EPM test (*n* = 8 mice). **i** Time spent in the crossing area in the EPM test (*n* = 8 mice). **j** Schematic diagram of the forced swim test. **k** Immobility time in the force swimming test (*n* = 8 mice). **l** Representative immunofluorescence staining for BrdU and NeuN double staining of the DG region of the hippocampus. **m** Representative immunofluorescence staining for DCX staining of the DG region of the hippocampus. In **d**, **e**, **h**, **i**, **k**, one-way ANOVA followed by Tukey’s posttest. Unless otherwise stated, data are presented as means ± SD. *p < 0.05, **p < 0.01, ***p < 0.001, and ****p < 0.0001.

The NZEP^*TA-Ce*^ has demonstrated potent potential in effectively reversing the inflammatory environment and microbial dysbiosis in the treatment of IBD. Additionally, 16S rRNA sequencing and metabolomics analyses suggest a potential link between NZEP^*TA-Ce*^ treatment and the alleviation of depressive and anxiety-like behaviors. To further evaluate its effectiveness in alleviating comorbid anxiety and depression in IBD, we used DSS-induced IBD mice. After 5 consecutive days of NZEP^*TA-Ce*^ treatment, we assessed the differences in anxiety and depressive-like behaviors between the NZEP^*TA-Ce*^ group and the model group (Fig. 10a). In the open field test (OFT), significant changes were observed in the total distance traveled by mice in the NZEP^*TA-Ce*^ group after treatment (Fig. 10b, c, and Supplementary Fig. 35a). Typically, longer time spent in the central area is interpreted as lower anxiety levels, while more time spent in the peripheral area indicates higher anxiety levels^65^. The average total time spent in the central area by the NZEP^*TA-Ce*^-treated mice was about 53.53 s, showing no significant difference compared to the normal group. In contrast, mice in the model group hardly spent any time in the central area (Fig. 10d). Conversely, mice in the model group spent up to 293.41 s in the peripheral area, whereas after NZEP^*TA-Ce*^ treatment, this time was significantly reduced to 252.90 s (*p* < 0.05), approaching the normal group’s time of 217.98 s (Fig. 10e). In the elevated plus maze (EPM) test (Fig. 10f), mice in the model group showed reduced exploration of the open arms, indicating decreased risk-taking behavior, and also spent significantly less time in the center area. However, the NZEP^*TA-Ce*^ group showed a significant increase in the time spent in the open arms and the number of crossings (Fig. 10g–i and Supplementary Figs. 35b, 36). In the Forced Swim Test (FST), the NZEP^*TA-Ce*^ group exhibited a notable reduction in immobility time compared to the model group^66^ (Fig. 10j, k). To further elucidate the anxiolytic and antidepressant mechanisms of NZEP^*TA-Ce*^, we evaluated *Il-1β*, *Il-6*, *Il-4*, and *Il-11* mRNA expression in the colon, blood, non-hippocampal brain regions, and hippocampus of DSS-induced IBD mice (Supplementary Fig. 37). We hypothesized that DSS-induced IBD would upregulate Il-1β and Il-6, correlating with increased neuroinflammation and synaptic dysfunction, which are linked to anxiety and depression behaviors. Elevated Il-1β disrupts neurogenesis and cognitive functions, while IL-6 dysregulation impairs serotonergic pathways and stress responses. Notably, NZEP^*TA-Ce*^ treatment significantly reduced Il-1β and Il-6 while upregulating Il-4 and Il-11, which promote protective effects on neurons. This shift towards an anti-inflammatory state counteracted neuroinflammatory damage.

To further evaluate the anxiolytic and antidepressant effects of NZEP^*TA-Ce*^ in mice, we examined the density of neurons in the hippocampus using immunofluorescence staining. Specifically, neuronal nuclei (NeuN), a marker for mature neurons, were combined with bromodeoxyuridine (BrdU) staining to identify newly formed neurons. Doublecortin (DCX), which encodes a microtubule-associated protein, is typically used as a marker for immature neurons. DSS administration significantly reduced the density of BrdU+/NeuN+ newly formed neurons and DCX+ immature neurons in the dentate gyrus (DG) region of the hippocampus. In contrast, NZEP^*TA-Ce*^ treatment significantly increased the number of BrdU+/NeuN+ and DCX+ cells in the hippocampus (Fig. 10l, m, and Supplementary Figs. 38–43). Collectively, those results indicate that DSS-induced IBD indeed leads to anxiety and depressive symptoms in mice, and NZEP^*TA-Ce*^ treatment effectively alleviated these anxiety and depressive-like behaviors.

## Discussion

In summary, we successfully constructed a series of NZEPs through an insect sclerotization-inspired strategy. Among them, NZEP^*TA-Ce*^ had the highest degree of nanozyme-exoskeletonization, which endows it with potent antioxidant and immunomodulatory properties. The ROS scavenging effect mediated by NZEP^*TA-Ce*^ enzyme-like activity (SOD-, CAT-) inhibited macrophage polarization to the M1 phenotype, while the immunomodulated properties of NZEP^*TA-Ce*^ components promoted macrophage polarization to the M2 phenotype. These properties allowed NZEP^*TA-Ce*^ to effectively disrupt the inflammatory vicious cycle, demonstrating potent anti-inflammatory efficacy. RNA sequencing analysis revealed that the potential mechanism by which NZEP^*TA-Ce*^ disrupted the vicious cycle of inflammation was the inhibition of NF-κB and MAPK-related inflammatory pathways. Thanks to the targeting of the NZEP^*TA-Ce*^ to macrophages at the site of inflammation, the NZEP^*TA-Ce*^ could be effectively retained and accumulate in the inflamed colonic regions after oral administration. These features of the NZEP^*TA-Ce*^ could synergistically reverse the homeostasis imbalance of intestinal inflammation and promote the repair of the intestinal barrier. Furthermore, 16S rRNA gene sequencing and untargeted metabolomics analysis confirmed that NZEP^*TA-Ce*^ treatment could achieve optimizing the diversity and composition of gut microbiota and improving metabolic functions related to neuroprotection, thereby alleviating anxiety and depressive symptoms associated with IBD. This study not only suggests an effective strategy to integrate nanozymes and yeast-derived polysaccharides but also develops a safe and encouraging therapeutic strategy for the management of IBD patients and associated psychological comorbidities.

## Methods

### Preparation of NZEP

Tannic acid (TA), epigallocatechin gallate (EGCG), gallic acid (GA), caffeic acid (CA), and ellagic acid (EA), zinc sulfate, palladium chloride, manganese sulfate, copper chloride, and ferrous sulfate, respectively, were used in the preparation of NZEP. As an example, the method of preparation of NZEP^*TA-Ce*^ was as follows: TA solution (100 μL, 24 mM), Ce (NO_3_)_3_∙6H_2_O solution (600 μL, 24 mM), and 40 mg YGPs were added to 50 mL deionized water with stirring for 3 h. Then the pH of the solution was adjusted to 8.0 by adding NaOH solution (2 mol/L). Then, the reaction was allowed to proceed for 12 h. Finally, the as synthesized NZEP^*TA-Ce*^ were collected by centrifugation and washed with deionized water three times. The other four NZEP were prepared in the same way.

### Evaluation of the regulatory effect of NZEP^*TA-Ce*^ on the cellular inflammatory state

RAW264.7 were seeded into 6- and 12-well plates severally at the density of 5 × 10^5^ cells/well and 2 × 10^5^ cells/well. After 24 h incubation, the cells were treated with LPS (200 ng/mL) and IFN-γ (20 ng/mL) for 12 h. Subsequently, the cells were incubated for another 12 h with the following groups:

(1) Model group (serum-free medium), (2) YGPs group (YGPs dispersed in serum-free medium at a concentration of 87 μg/mL), (3) TA-Ce group (TA-Ce dispersed in serum-free medium at a concentration of 63 μg/mL), (4) NZEP^*TA-Ce*^ group (NZEP^*TA-Ce*^ dispersed in serum-free medium at a concentration of 150 μg/mL). The cells without any treatment throughout were used as the normal group.

The cells seeded in 12-well plates were then used to perform cellular immunofluorescence staining experiments to detect protein expression of CD86 (M1 marker) and CD206 (M2 marker). In this experiment, cells were first fixed with 4% paraformaldehyde. After washing with PBST (PBS with 0.05% Tween 20) for three times, cell round coverslip were permeabilized and blocked with 5% BSA in PBS for 1 h at 37 °C. For immunofluorescence staining, tissue sections were incubated with primary antibody at 4 °C overnight. The cell round coverslip were incubated with the secondary antibody for 1 h at 37 °C and finally blocked with an anti-fluorescence quencher containing DAPI (Yeasen, China). Images were acquired with a CLSM (Cai Zeiss, Germany). The following primary antibodies were used: CD86 (Abclonal, 1:100), CD206 (Proteintech, 1:500). The cells in 6-well plates were de-labeled with CD86 and CD206 antibodies and then subjected to flow cytometry analysis to quantify cell polarization levels.

### Therapeutic efficacy of NZEP^*TA-Ce*^ against DSS-induced IBD

#### Establishment and treatment of DSS-induced IBD model

First, IBD was induced by the C57BL/6J mice (male, 8–10 weeks), and the mice were given different group samples to evaluate the therapeutic efficacy of NZEP^*TA-Ce*^ against IBD. Briefly, the mice were given drinking water containing 3% DSS for 7 days to induce IBD. Then the mice were randomly assigned to five groups (normal, model, YGPs, TA-Ce, and NZEP^*TA-Ce*^; *n* = 5 mice per group). Subsequently, the mice were fed normal drinking water and divided into the following groups for oral administration for 5 days:

(1) Model group (PBS), (2) YGPs group (YGPs dispersed in PBS at a concentration of 87 μg/mL), (3) TA-Ce group (TA-Ce dispersed in PBS at a concentration of 63 μg/mL), (4) NZEP^*TA-Ce*^ group (NZEP^*TA-Ce*^ dispersed in PBS at a concentration of 150 μg/mL).

The administration volume for all mice was 0.1 mL/10 g body weight. Meanwhile, mice without any treatment throughout were used as the normal group.

Then the mice were euthanized, and the length of the colon was measured, and the weight of the spleen was measured. Colon tissues were harvested and separated into several sections for further analysis. Moreover, the body weights of all the mice were recorded every 2 days.

### Evaluation the potential neuroprotective effects of NZEP^*TA-Ce*^ in DSS-induced IBD model

#### Establishment and treatment of DSS-induced IBD

The C57BL/6J mice (male, 8–10 weeks) were given drinking water containing 3% DSS for 7 days to induce IBD. After the model was successfully established, the mice were randomly divided into the following groups for oral administration 5 days:

(1) Model group (PBS), (2) NZEP^*TA-Ce*^ group (NZEP^*TA-Ce*^ dispersed in PBS at a concentration of 150 μg/mL). Meanwhile, mice without any treatment throughout were used as the normal group. Afterwards, anxiety and depressive behavioral changes were conducted.

### Reporting summary

Further information on research design is available in the Nature Portfolio Reporting Summary linked to this article.

## Supplementary information

Supplementary Information

Reporting Summary

Transparent Peer Review File

## Data Availability

All data generated or analysed during this study are included in this published article and its Supplementary Information files. Source data underlying Figs. 1–10 and Supplementary Figs. 1–43 are available in Figshare (https://doi.org/10.6084/m9.figshare.28674176). RNA-seq data from RAW264.7 cells and mouse fecal 16S rRNA gene sequencing data have been deposited in the NCBI BioProject database under accession codes PRJNA1442071 and PRJNA1442011, respectively, with raw sequencing data available in the Sequence Read Archive (SRA). Metabolomics data have been deposited in the MetaboLights repository under accession code MTBLS14127. All data underlying this study are available from the corresponding author upon request.
