# Supplementary material for: Bioinspired nanozyme-exoskeletonized yeast-derived polysaccharides for treating comorbid anxiety and depression in inflammatory bowel disease
Source: Nat Commun. 2026 Oct 14;17:11594. doi: 10.1038/s41467-026-78633-3 (PMC13752268; doi:10.1038/s41467-026-78633-3)
Supplement: Supplementary file 1 [file 41467_2026_78633_MOESM1_ESM.pdf]

Supplementary Information

Bioinspired Nanozyme-Exoskeletonized Yeast-derived Polysaccharides for Treating Comorbid Anxiety and Depression in Inflammatory Bowel Disease

Fan Zhang[1,2]†, Lei Chen[1,2]†[*], Shengfu Wang[2], Xinyuan Chen[2], Kaiyi Du[2], Fuqiang Song[2], Linyuan Jiang[2], Yanzhen Feng[2], Siting Du[2], Tianyao Jiang[2], Yong Liu[3], Jian Xiao[1,2*]*

[1]Department of Wound healing, The First Affiliated Hospital of Wenzhou Medical University, Wenzhou, Zhejiang 325035, China.

[2]Oujiang Laboratory (Zhejiang Lab for Regenerative Medicine, Vision and Brain Health), School of Pharmaceutical Sciences, Wenzhou Medical University, Wenzhou, Zhejiang, 325035, China.

[3]State Key Laboratory of Medicinal Chemical Biology, Academy for Advanced Interdisciplinary Studies, Nankai International Advanced Research Institute (Shenzhen Futian), Nankai University, Tianjin 300071, China

[*]Corresponding author. Email: xfxj2000@126.com (J. X.), leichen_1994@wmu.edu.cn (L. C.), y.liu@nankai.edu.cn (Y. L.).

†These authors contributed equally to this work.

This PDF file includes:

Supplementary Methods

Supplementary Figures 1-43

Supplementary Tables 1–6

Supplementary Methods

Animals

All animal experiments were conducted in compliance with the Chinese National Standard and were approved by the Laboratory Animal Ethics Committee of Oujiang Laboratory (No. OJLAB24031201 and OJLAB24082701). C57BL/6J mice (male, 6–8 weeks, 20–22 g) were sourced from Zhejiang Vital River Laboratory Animal Technology Co., Ltd. The $CD4^{+}CD45RB^{hi}$ T cell transfer-induced IBD model was established by Biocytogen Pharmaceuticals (Beijing) Co., Ltd. All animals were housed under specific pathogen-free (SPF) conditions with a 12 h light/12 h dark cycle, controlled ambient temperature (22 ± 2 °C), and relative humidity of 50 ± 10%, with free access to food and water. Sex was reported for each animal model; however, the study was not designed to compare sex-dependent differences, as different models employed sex-specific cohorts according to predefined experimental conditions.

Materials

Tannic acid (T292288, USP grade), Epigallocatechin gallate (E107403, analytical standard), gallic acid (G131992, ≥ 99%), caffeic acid (C108307, ≥ 99%) and ellagic acid (E102710, ≥ 96%), $Ce(NO_3)_3·6H_2O$ (C105378, ≥ 99.95%), Methanol (M116115, ≥ 99.5%) and Ethanol (A171299, ≥ 75%) were purchased from Aladdin Biochemical Technology Co., Ltd. (Shanghai, China). Luria-Bertani (LB) broth (L8291), PBS (p1020), Modified Hematoxylin-Eosin Stain Kit (G1121-4) was purchased from Solarbio Science & Technology Co., Ltd. (Beijing, China). Lipopolysaccharide (LPS, L2880) were purchased from Sigma-Aldrich (MO, USA). Interferon-γ (IFN-γ, 50709-MNAH, > 85%) was purchased from Sino Biological Co., Ltd. (Beijing, China). Calcein/PI Cell Viability/Cytotoxicity Assay Kit, cell counting kit-8 (CCK-8) and Dil cell membrane fluorescent probe were purchased from Beyotime Biotechnology Co., Ltd. (Shanghai, China).

Cell

Mouse monocyte macrophage leukemia (RAW264.7) cells (Cat. No. CL-0190) and human colorectal adenocarcinoma (Caco2) cells (Cat. No. CL-0050) at were purchased

from Procell Life Science & Technology Co., Ltd. RAW264.7 cells were cultured in DMEM supplemented with 1% streptomycin/penicillin and 10% fetal bovine serum (FBS, Gibco) in a cell incubator (37 °C, humidified atmosphere of 5% CO_2). And Caco2 cells were cultured in DMEM/F12 supplemented with 1% streptomycin/penicillin and 10% fetal bovine serum (FBS, Gibco) in a cell incubator (37 °C, humidified atmosphere of 5% CO_2). All materials used in the cell experiments were sterilized by UV irradiation sterilization before use.

Characterizations

Particle size and zeta potential were measured on a Zetasizer Ultra (Malvern, UK). The morphologies of NZEP were observed on Talos F200S (Thermo Scientific, US) transmission electron microscope. The Ce content of NZEP was determined by thermogravimetric analysis (TGA, Mettler, USA) and XPS (Thermo Scientific Escalab 250Xi). For TGA, samples were heated from room temperature to 800 °C at a heating rate of 5 °C min^{-1} under ambient air. The chemical and crystal structure of NZEP were characterized by Fourier transformation infrared spectrometer (FTIR, Nicolet Nexus is50, ThermoScientific) and X-ray diffraction analysis (XRD, D8 ADVANCE, BRUKER).

Preparation of yeast glucan particles (YGPs)

YGPs were prepared from Baker's yeast. Firstly, the yeast cells are suspended in a 1 M NaOH solution and heated for 1 h at 90 ℃ with constant stirring. Next, the cells were centrifuged at 1000 × g for 10 min, and the supernatant was discarded. The collected cell precipitate was then further treated academically with an HCl solution (pH = 4.5). The cell precipitate was incubated at 75 ℃ for 2 h. Thirdly, the samples were centrifuged again and washed thoroughly three times with deionized water, isopropanol, acetone. Finally, the collected cells were washed with isopropanol and acetone. Vacuum drying was then used to produce a white-like powder containing the YGPs.

***In vitro* antioxidant activity study of NZEP$^{TA\text{-}Ce}$**

2,2'-Azinobis-(3-ethylbenzthiazoline-6-sulphonate) (ABTS) radical scavenging activity was measured by ABTS method. Briefly, ABTS radicals were generated by

reacting 7 mM ABTS stock solution with 2.45 mM potassium persulfate in the dark for 16 h. Then, the ABTS radical solution was diluted by PBS to reach a proper absorbance at 734 nm. Different concentrations of the NZEP$^{TA\text{-}Ce}$ solution (25, 50, 75, 100, 125, 150 μg/mL, 1 mL) were mixed with 1 mL ABTS solution, and placed in the dark for 5 min at room temperature. Then, the UV-vis spectrum at 500-800 nm was detected by microplate reader to collect the optical density (OD) at 734 nm. The ABTS radical scavenging rate was calculated by this calculation formula: ABTS radical scavenging rate (%) = [$OD_{control} - OD_{sample}$] / $OD_{control}$ ×100%.

2,2-Diphenyl-1-picrylhydrazyl (DPPH) radical scavenging activity was measured by the DPPH. Firstly, the DPPH radical stock solution (0.2 mg/mL) was prepared in methanol. Then, different concentrations of the NZEP$^{TA\text{-}Ce}$ solution (25, 50, 75, 100, 125, 150 μg/mL, 1 mL) were mixed with 1 mL DPPH solution. The resulting solution was shaken vigorously and incubated in the dark for 10 min at room temperature. Afterwards, the UV-vis spectrum at 500-800 nm was detected by microplate reader to collect the $OD_{517\ nm}$ values. The DPPH radical scavenging rate was calculated by this calculation formula: DPPH radical scavenging rate (%) = [$OD_{control} - OD_{sample}$] / $OD_{control}$ ×100%.

The concentration of H_2O_2 was monitored at 240 nm by the UV-Vis spectrophotometer was used to evaluate H_2O_2 scavenging ability. Different concentrations of the NZEP$^{TA\text{-}Ce}$ solution (25, 50, 75, 100, 125, 150 μg/mL, 1 mL) were mixed with 1 mL 10 mM H_2O_2 solution. Then the resulting solution was shaken vigorously and incubated in the dark for 30 min at room temperature. Afterwards, the absorbance peak at 240 nm of the solution was monitored by using a UV-vis spectroscopy. The H_2O_2 scavenging rate was calculated by this calculation formula: H_2O_2 scavenging rate (%) = [$OD_{control} - OD_{sample}$] / $OD_{control}$ ×100%.

Hydroxyl Free Radical (·OH) assay kit (Nanjing Jiancheng Bioengineering Institute, Nanjing, China) was used to evaluate ·OH scavenging activity. Different concentrations of the NZEP$^{TA\text{-}Ce}$ solution (25, 50, 75, 100, 125, 150 μg/mL, 0.2 mL) were mixed with the working solution, and placed in the dark for 1 min at 37 °C. Then, the chromogenic

agent was added immediately to terminate the reaction. The resulting solution was incubated for 20 min at room temperature. Finally, the UV-vis spectrum at 500-800 nm was detected by microplate reader to collect the OD values at 550 nm. ·OH radical scavenging rate (%) = [$OD_{control}$ – OD_{sample}] / $OD_{control}$ ×100%.

Inhibition and produce superoxide anion ($\cdot O_2^-$) assay kit (Nanjing Jiancheng Bioengineering Institute, Nanjing, China) was used to evaluate $\cdot O_2^-$ scavenging activity. Different concentrations of the $NZEP^{TA\text{-}Ce}$ solution (25, 50, 75, 100, 125, 150 μg/mL, 0.05 mL) were mixed with the working solution, and placed in the dark for 1 min at 37 °C. Then, the chromogenic agent was added immediately to terminate the reaction. The resulting solution was incubated for 20 min at room temperature. Finally, the UV-vis spectrum at 500-800 nm was detected by microplate reader to collect the OD values at 550 nm. $\cdot O_2^-$ radical scavenging rate (%) = [$OD_{control}$ – OD_{sample}] / $OD_{control}$ ×100%.

Oral Stability

The oral stability of $NZEP^{TA\text{-}Ce}$ and TA-Ce was evaluated in simulated gastric (pH 1.2, 0.04 M HCl, 2 h), intestinal (pH 6.8, 0.1 M PBS buffer, 4 h), and colon fluids (normal: pH 7.4, 0.1 M PBS buffer; IBD: pH 3.5, 0.1 MPBS buffer adjusted with HCl, 42 h). 10 mg $NZEP^{TA\text{-}Ce}$ or 10 mg TA-Ce were dispersed in simulated gastric and intestinal fluids, respectively, in the order described above, and placed on a shaker and incubated at 37 °C for the corresponding time. The samples were subsequently collected by centrifugation and used for TEM, DLS and enzyme activity assay experiments.

Biocompatibility study of the of $NZEP^{TA\text{-}Ce}$

Cytotoxicity

Two different cell lines were cultivated and used for the cytocompatibility experiments: RAW264.7 and Caco2 (5×10^4 cells) were cultured in a 24-well plate and incubated at 37 °C for 24 h. Then, the old medium was replaced with the fresh medium (Control) or the samples (YGPs, TA-Ce or $NZEP^{TA\text{-}Ce}$), followed by continuous incubation at 37 °C for 24, 48, and 72 h, respectively. After the culture solution was replaced with the WST-8 reagent for 1 h at 37 °C, the absorbance at 450 nm was detected by microplate reader. Meanwhile, live/dead cell staining assay was also carried out to evaluate the

cytocompatibility of NZEP$^{TA\text{-}Ce}$.

Hemolysis Rate Evaluation

Fresh red blood cells (RBCs) were obtained from C57BL/6 mice, and diluted by normal saline. Then, 1 mL RBCs suspension was mixed with 1 mL normal saline (negative control), Triton X-100 (positive control) and different concentrations of sample groups (YGPs, TA-Ce or NZEP$^{TA\text{-}Ce}$), which were preheated at 37 °C for 30 min. The mixtures were further incubated for 1 h at 37 °C, then dripped onto a blood smear, and observed using an inverted microscope to assess the integrity of blood cells. The rest of mixed solutions were centrifugated (800 x g, 5 min), and the supernatants were carefully collected for $OD_{545\ nm}$ measurement. The hemolysis ratio was calculated by this calculation formula: Hemolysis (%) = $[OD_{sample}\text{-}OD_{negative\ control}]$ / $[OD_{positive\ control}\text{-}OD_{negative\ control}]$ ×100%.

Evaluation of the oral safety of NZEP$^{TA\text{-}Ce}$

Firstly, healthy C57BL/6J mice (male, 8-10 weeks) were randomly divided into two groups: control group and NZEP$^{TA\text{-}Ce}$ group (n = 5 mice per group). Control group and NZEP$^{TA\text{-}Ce}$ group were administered 200 μL of saline or NZEP$^{TA\text{-}Ce}$ (150 mg/kg body weight) orally to the mice in the respective groups. Mice were monitored for 30 days to assess *in vivo* biosafety. At specific time intervals, mice were euthanized and collected blood samples were subjected to whole blood cytometric analysis and serum biochemical assays. Major organs, including heart, liver, spleen, lungs, and kidneys, were harvested, fixed with 10% paraformaldehyde, and paraffin sectioned at a thickness of approximately 7 μm for H&E staining.

Evaluation of selective cellular uptake of NZEP$^{TA\text{-}Ce}$

First, FITC-NZEP$^{TA\text{-}Ce}$ was prepared. Briefly, 5 mg of NZEP$^{TA\text{-}Ce}$ was homogeneously dispersed in a beaker containing 50 mL of water. Subsequently, FITC-NHS (1 mg/mL) dissolved in 20 μL of DMSO was added to the beaker, stirred overnight and then centrifuged at 2300 x g with water and washed three times.

For qualitative analysis of cellular uptake, RAW264.7 cells and Caco2 cells were seeded in confocal dishes at a density of 2×10^5 cells/well and then incubated with FITC-

NZEP^{TA-Ce} (150 μg/mL) in FBS-free culture medium for 0.5, 1, and 2 h at 37 °C. After being rinsed gently 3 times with PBS, the cells were detected by confocal laser scanning microscope (CLSM). In addition, RAW264.7 cells and Caco2 cells were incubated with 200 ng/mL LPS and 20 ng/mL IFN-γ for 12 h and then incubated with FITC-NZEP^{TA-Ce} for different times, and then FITC-NZEP^{TA-Ce} uptake by activated macrophages was observed under laser confocal microscope.

To quantify the efficiency of cellular uptake, RAW264.7 cells and Caco2 cells were inoculated into 6-well plates at a density of 2×10^5 cells/well, respectively, and then incubated with FITC-NZEP^{TA-Ce} for different times at 37 °C, respectively, and then rinsed three times with PBS to prepare single-cell suspensions, which were finally analyzed by flow cytometry.

To investigate whether the cellular uptake of NZEP^{TA-Ce} is dependent on glucan receptors, RAW 264.7 cells were pretreated with 1×10^{-5} M of anti-dectin-1, anti-TLR2, anti-CD11b and anti-CD18 blocking antibody for 1 h, followed by incubation with 200 ng/mL LPS and 20 ng/mL IFN-γ for 12 h and then incubated with FITC-NZEP^{TA-Ce} for 12 h, which were used for confocal imaging and flow cytometry.

Evaluation of the regulation of cellular oxidative stress and inflammation by NZEP^{TA-Ce}

Evaluation of the regulatory effect of NZEP^{TA-Ce} on cellular oxidative stress

RAW 264.7 were seeded into 6-, 12-, and 24-well plates severally at the density of 5×10^5 cells/well, 2×10^5 cells/well, and 1×10^5 cells/well. After 24 h incubation, the cells were treated with 100 μM tert-Butyl Hydroperoxide (tBHP) at 37 °C for 4 h. The cells were incubated for another 12 h with the following groups:

(1) Model group (Serum-free medium), (2) YGPs group (87 μg/mL of YGPs dispersed in serum-free medium), (3) TA-Ce group (63 μg/mL of TA-Ce dispersed in serum-free medium), (4) NZEP^{TA-Ce} group (150 μg/mL of NZEP^{TA-Ce} dispersed in serum-free medium). In addition, cells without any treatment throughout were used as the Normal group.

Then, the cells seeded in 24-well plates were incubated with cell counting kit-8 to detect the cell viability. The cells seeded in 12-well plates were gently rinsed thrice with

serum-free medium, followed by addition of 10 μM DCFH-DA solution for 30 min's incubation at 37 °C in the dark. Afterwards, the cells were washed with serum-free medium thrice to remove unloaded DCFH-DA probe, and then imaged by laser confocal microscope. Besides, the cells seeded in 6-well plates were incubated with the DCFH-DA probe in the same way above, and then performed by flow cytometry analysis to quantify intracellular ROS levels separately.

Transcriptome sequencing and data analysis

RAW264.7 were seeded into 6-well plates severally at the density of 5×10^5 cells/well. After 24 h incubation, the cells were treated with LPS (200 ng/mL) and IFN-γ (20 ng/mL) for 12 h. Then, the cells were treated with 100 μM tBHP at 37 °C for another 4 h. Finally, the Model group (Serum-free medium) and NZEP$^{TA\text{-}Ce}$ group (150 μg/mL of NZEP$^{TA\text{-}Ce}$ dispersed in serum-free medium) were added into the wells and incubated for another 12 h. Finally, the cells were gently washed three times with PBS and RNA was extracted with an RNA extraction kit, and the extracted RNA samples were stored at -80 °C for subsequent transcriptome analysis. The differential expression analysis of two conditions groups (three biological replicates per condition) was performed using the DESeq2 R package (1.20.0). Gene Ontology (GO) enrichment analysis of differentially expressed genes was implemented by the clusterProfiler R package (3.8.1), in which gene length bias was corrected. GO terms with corrected P value less than 0. 05 were considered significantly enriched by differential expressed genes. And we used clusterProfiler R package (3.8.1) to test the statistical enrichment of differential expression genes in Kyoto Encyclopedia of Genes and Genomes (KEGG) pathways. In addition to this, the validation experiments for the DEGs (*Il-1a*, *Il-1b*, *Il-6*, *Nos2*, *Tnfa*, *Il-4*, *Il-11*, *Tgfb3*, *Fem1a*, and *Akap1*) used cellular immunofluorescence assay and RT-PCR. The mRNA primer sequences used are listed in Supplementary Table 1.

Retention and targeting experiments of NZEP$^{TA\text{-}Ce}$ in IBD.

First, NZEP$^{TA\text{-}Ce}$ labeled with Cy5.5-NHS was prepared. Briefly, 5 mg of NZEP$^{TA\text{-}Ce}$ was homogeneously dispersed in a beaker containing 50 mL of water. Subsequently, Cy5.5-NHS (1 mg/mL) dissolved in 20 μL of DMSO was added to the beaker, stirred

overnight and then centrifuged at 2300 x g with water and washed three times.

C57BL/6J male mice were randomly divided into Normal group and DSS group. DSS group's mice were induced to IBD by feeding them 3% (w/v) DSS dissolved in drinking water for 5 consecutive days. Both groups of mice were administered with Cy5.5 labeled NZEP$^{TA\text{-}Ce}$ (150 μg/mL, 0.1 mL/10g body weight) via gavage. Fluorescence of mice was detected by IVIS after 6, 12, 18, and 24 hours of administration of NZEP$^{TA\text{-}Ce}$ labeled with Cy5.5. In addition, all mice were euthanized and tissues such as colon, heart, liver, spleen, lungs, and kidneys were collected for further in vitro imaging.

Therapeutic efficacy of NZEP$^{TA\text{-}Ce}$ against DSS-induced IBD

Inductively coupled plasma atomic emission spectroscopy

At the end of the study, the biodistribution of cerium was studied using blood of mice. The iron and cerium ions contents were determined using ICP-OES.

Histopathology study

The histopathology analysis for evaluating colon damage was performed according to standard procedures for paraffin embedding and H&E staining. Briefly, the colonic tissues were fixed in 4% paraformaldehyde solution, embedded in paraffin, sectioned (7 μm), and stained with H&E. The resulting slides were scanned using a virtual slide microscope (Olympus VS200). Each section was scored blindly by the experimenter for histological evidence of colon damage by DSS with a scoring system described in Supplementary Table 2 including the severity of inflammation, depth of injury, the extent of crypt damage, and percentage of colon tissue involvement.

Evaluation of MPO activity in colon tissue

MPO was measured to evaluate the level of inflammation in colon tissues. The evaluation of MPO activity was based on the Myeloperoxidase (MPO) Activity Assay kit (Solarbio, Beijing). Briefly, the colon tissues were isolated from mice and were weighed and washed thoroughly with PBS to clean the fecal matter. Next, the samples were homogenized in sample preparation solution, and sonicated for 10 s to give a homogeneous tissue suspension. Afterward, the clear supernatant was collected by

centrifugation at a speed of 12000 g at 4 °C for 10 min. Subsequently, mixing 20 μL of sample with the working solution as directed by the kit manufacturer's instructions and the plate was incubated for 10 min at 60 °C. Then, OD 460 values were measured to represent the MPO expression levels.

Evaluation of MDA activity in colon tissue

The evaluation of MDA activity was based on the Malondialdehyde (MDA) Content Assay Kit (Solarbio, Beijing). Tissue homogenates of colon tissue were prepared by the same procedure as in the MPO experiment. As directed by the kit manufacturer's instructions, the MDA working solution was prepared, followed by mixing 100 μL of the processed sample with 300 μL of working solution and placing the reaction at 100 °C for 1 h. Subsequently, the sample was cooled to room temperature and centrifugation at a speed of 10,000 g. The supernatant was taken in a 96-well plate, and OD 532 values and OD 600 values were measured to calculate the MDA content.

Gut microbiome analysis

Feces samples of the five group mice were collected for 16S rDNA sequencing analysis. The library sequencing and data processing were conducted by OE biotech Co., Ltd. (Shanghai, China). Raw sequencing data were in FASTQ format. Paired-end reads were then preprocessed using Cutadapt software to detect and cut off the adapter. After trimming, paired-end reads were filtering low quality sequences, denoised, merged and detect and cut off the chimera reads using DADA2 with the default parameters of QIIME2 (2020.11). At last, the software output the representative reads and the ASV abundance table. The representative read of each ASV was selected using QIIME2 package. All representative reads were annotated and blasted against Silva database (Version 138) using q2-feature-classifier with the default parameters. QIIME2 software was used for alpha and beta diversity analysis. The microbial diversity in samples was estimated using the alpha diversity that include Chao1 index and Shannon index. The unweighted Unifrac distance matrix performed by R package was used for unweighted Unifrac Principal coordinates analysis (PCoA) to estimate the beta diversity. Then the R package was used to analyze the significant differences between different groups

using ANOVA/Kruskal Wallis/T test/Wilcoxon statistical test. The linear discriminant analysis effect size (LEfSe) method was used to compare the taxonomy abundance spectrum.

Untargeted metabolomics sequencing and analysis

Metabolome sequencing and analysis were performed at OE biotech Co., Ltd. (Shanghai, China). Metabolites were extracted from the feces of each group of mice and then detected using LC-MS/MS. The raw files obtained by mass spectrometry were first imported into Compound Discoverer 3.1 software for spectrogram processing and database searching to obtain qualitative and quantitative results of metabolites, and then the data were subjected to quality control to ensure the accuracy and reliability of the data results. Inter-sample correlation analysis, differential gene clustering and KEGG enrichment analysis were then performed.

Immunofluorescence staining

The distal colon segments, fixed in 4% paraformaldehyde, were sliced and trimmed into serial sections. The slides were deparaffinized in xylene and rehydrated through ascending ethanol series. Subsequently, the sections were subjected to antigen retrieval using citrate buffer (0.01 M, pH = 6.0) in a steamer at 95 °C for 10 min. Then, the sections were treated with 3% H_2O_2 for 30 min to quench endogenous peroxidase. After washing with PBST (PBS with 0.05% Tween 20) for three times, slides were permeabilized and blocked with 5% BSA in PBS for 1 h at 37 ℃. For immunofluorescence staining, tissue sections were incubated with primary antibody at 4 °C overnight. The slides were incubated with the secondary antibody for 1 h at 37 °C and finally blocked with an anti-fluorescence quencher containing DAPI (Yeasen, China). Images were acquired with a CLSM (Cai Zeiss, Gremany). The following primary antibodies were used: CD86 (Abclonal, 1:100), CD206 (Proteintech, 1:500), zonula occludens-1 (Proteintech, 1:1000) and Occludin (Proteintech, 1:1000).

Quantitative reverse transcription polymerase chain reaction (qRT-PCR)

To further elucidate the anxiolytic and antidepressant mechanisms of NZEP$^{TA\text{-}Ce}$, we assessed IL-1β, IL-6, IL-4, and IL-11 mRNA expression in the colon, blood, non-

hippocampal brain regions, and hippocampus of DSS-induced IBD mice. colon, blood, non-hippocampal brain regions, and hippocampus were harvested and treated with RNA Extraction Kit (Solarbio) for RNA extraction. HiScript III RT SuperMix for qPCR (+gDNA wiper) (Vazyme) was used to generate cDNA. qRT-PCR assay was performed with Taq Pro Universal SYBR qPCR Master Mix (Vazyme).

Evaluation of the therapeutic effect of NZEP$^{TA\text{-}Ce}$ in the CD4^{+}CD45RB^{hi} T Cell Transfer Model

To further investigate the therapeutic effect of NZEP$^{TA\text{-}Ce}$, we employed the CD4^{+}CD45RB^{hi} T cell transfer-induced IBD model. The model was established by Biocytogen Pharmaceuticals (Beijing) Co., Ltd. The day of T cell isolation and transfer was designated as Day 0. A total of six female BALB/c mice (8-9 weeks old, 14-22 g) were used as donor mice. After euthanasia, spleens were harvested and processed into single-cell suspensions.

CD4^{+} T cells were first enriched using the CD4^{+} T Cell Isolation Kit (Miltenyi Biotec, Cat# 130-104-454), followed by depletion of CD25^{+} cells using the CD25 MicroBead Kit (Miltenyi Biotec, Cat# 130-091-072). Cell separation was carried out using Miltenyi LS Columns (Miltenyi Biotec, Cat# 130-042-401) following the manufacturer’s protocol. Subsequently, CD4^{+}CD25^{-} cells were stained with anti-CD4 antibody (Invitrogen, Cat# 47-0042-82, APC conjugated) and anti-CD45RB antibody (Invitrogen, Cat# 11-0455-82, FITC conjugated), and sorted by flow cytometry to isolate CD4^{+}CD45RB^{hi} T cells. Flow cytometric sorting was performed on an Attune® NxT Flow Cytometer (Invitrogen) to confirm cell purity. Thirty female severe combined immune deficiency (SCID) mice (8-9 weeks old, 14-22 g) were used as recipients. Among them, twenty mice received an intravenous injection of 5×10^{5} purified CD4^{+}CD45RB^{hi} T cells per mouse. The remaining ten SCID mice were used as the Normal group without T cell transfer. After cell transfer, mice were monitored for 6 weeks (Day 0 to Day 40). Clinical symptoms including body weight, stool consistency, and fecal blood were recorded every 10 days. The development of colitis was defined by the appearance of significant weight loss, diarrhea, and/or hematochezia.

After the model has been formed, the mice were randomly assigned to Three groups (Normal, Model and NZEP$^{TA\text{-}Ce}$; n = 10 mice per group). Subsequently, the mice were divided into the following groups for oral administration for 5 days: (1) Model group (PBS), (2) NZEP$^{TA\text{-}Ce}$ group (NZEP$^{TA\text{-}Ce}$ dispersed in PBS at a concentration of 150 μg/mL). Then the mice were euthanized and the length of the colon was measured and the weight of the spleen was measured. Colon tissues were harvested and separated into several sections for further analysis. Moreover, the body weights of all the mice were recorded every two days.

Flow cytometry was performed to analyze immune cell subsets in the lamina propria, including Th17 ($CD3^+$, $CD45^+$ $CD4^+$, $FoxP3^-$, IL-$17A^+$), Th1 ($CD3^+$, $CD45^+$ $CD4^+$, $FoxP3^-$, IFN-γ^+), and Treg cells ($CD3^+$, $CD45^+$ $CD4^+$,$FoxP3^+$). Briefly, the mouse colon was excised, cleaned of mesenteric fat, and opened longitudinally. To remove epithelial cells, the tissue was incubated in HBSS containing 5 mM EDTA and 1 mM dithiothreitol (DTT) at 37 °C with shaking. The remaining lamina propria (LP) tissue was digested using a solution containing collagenase IV (0.05 g /100 mL), DNase I (0.05 g /100 mL), and dispase, followed by vigorous vortexing and filtration through a 70 μm cell strainer. The resulting cell suspension was subjected to 40% and 80% Percoll gradient centrifugation for purification. The isolated LP mononuclear cells were then resuspended in FACS buffer (3% FBS in PBS) for flow cytometry. The following fluorochrome-conjugated monoclonal antibodies were used for staining: anti-CD3e, anti-CD45, anti-CD4, anti-Foxp3, anti-IFN-γ, anti-IL-17A. Fixation and permeabilization for Foxp3 and cytokine detection were carried out using anti-Foxp3 antibody and Intracellular Fixation/Permeabilization Buffer Kit (Elabscience), according to the manufacturer's instructions. Information on the antibodies used in this experiment was shown in Supplementary Table 3, and information on the remaining reagents was shown in Supplementary Table 4.

Immunofluorescence staining was conducted to detect IL-7 expression in colonic tissues. The relative expression levels of IFN-γ, IL-17, IL-21, IL-10, TGF-β, and IL-4 were determined using ELISA kits, following the manufacturers' protocols.

Evaluation the potential neuroprotective effects of NZEP$^{TA\text{-}Ce}$ in DSS-induced IBD model

Open field test

The open field test was employed to quantify anxiety-like behaviors. Each mouse was positioned at the center of a box measuring 50 cm in diameter and 40 cm in height. Their movements were documented for 5 min within a dimly illuminated environment, utilizing a camera system. The ensuing parameters were assessed: the distance covered and the duration spent in each distinct zone (corner, periphery, and center).

The elevated plus maze

The anxiety-like behaviors of mice were evaluated using the elevated plus maze, which comprised two open arms and two closed arms arranged perpendicularly in a cross configuration. The maze itself was elevated 40 cm above the floor. Mice were positioned in the central square, facing the open arms for 5 minutes. Throughout this period, the movements of the mice were recorded utilizing video tracking software. The amount of time spent in both the open and closed arms within the 5-minute timeframe was used to gauge anxiety levels.

Forced swimming test

To assess depression-like behaviors, the forced swimming test was implemented. Mice were situated within a cylindrical apparatus boasting a diameter of 10 cm, wherein the water temperature was maintained at 25 °C and the depth reached 30 cm. The immobility time, during which the mice exhibited a floating posture without any signs of struggle, with only their heads remaining above the water surface, was recorded by the camera system for 6 minutes.

Immunofluorescence staining

The mice were anesthetized using intraperitoneal injection of pentobarbital sodium (60 mg/kg, Sigma), followed by cardiac perfusion with pre-cooled 0.9% NaCl and 4% paraformaldehyde (PFA). The brain tissues were fixed in 4% PFA, dehydrated in graded ethanol, cleared in xylene, and paraffin-embedded. The paraffin blocks were sliced into serial sections (10 μm) and mounted onto glass slides. The slides were deparaffinized

in xylene and rehydrated through descending ethanol series. Antigen retrieval was performed using citrate buffer (0.01 M, pH = 6.0) in a steamer at 95 °C for 10 min. For BrdU staining, sections were incubated with 2 N HCl for 30 min at room temperature and neutralized with 0.1 M borate buffer (pH = 8.5). Sections were then treated with 3% H_2O_2 for 30 min to quench endogenous peroxidase. After washing with PBST (PBS with 0.05% Tween-20) three times, the sections were permeabilized and blocked with 5% BSA in PBS for 1 h at 37 °C. Tissue sections were incubated with primary antibodies either for BrdU and NeuN (double staining) or for DCX (single staining) on separate sections at 4 °C overnight. The slides were then incubated with the corresponding fluorophore-conjugated secondary antibodies for 1 h at 37 °C and counterstained with an anti-fluorescence quencher containing DAPI (Yeasen, China).

Images were acquired using a Zeiss Axioscan 7 automatic digital slide scanner equipped with a Plan-Apochromat 10× objective (NA 0.45) at a scanning resolution of 0.345 μm/pixel. BrdU/NeuN double staining was imaged in DAPI, FITC, and Cy3 channels, while DCX staining was imaged in DAPI and Cy3 channels. The panel shown in Figure 10m was generated by cropping a 1000 × 500 px ROI. The ROI Center offset was (0 μm, 0 μm). The corresponding physical size of the cropped ROI is 691.21 μm × 354.60 μm. Images were exported as TIFF with LZW (lossless) compression. To improve visibility, images were adjusted linearly and uniformly in ImageJ 1.48 using "Brightness/Contrast" with Min = 0, Max = 200, and Gamma = 1.0 (unchanged). The same Min/Max settings were applied identically to all comparable images (the Figure 10m). We did not click "Apply", so these adjustments affected display only and did not overwrite pixel values. Export format remained TIFF.

Supplementary Figures and Tables

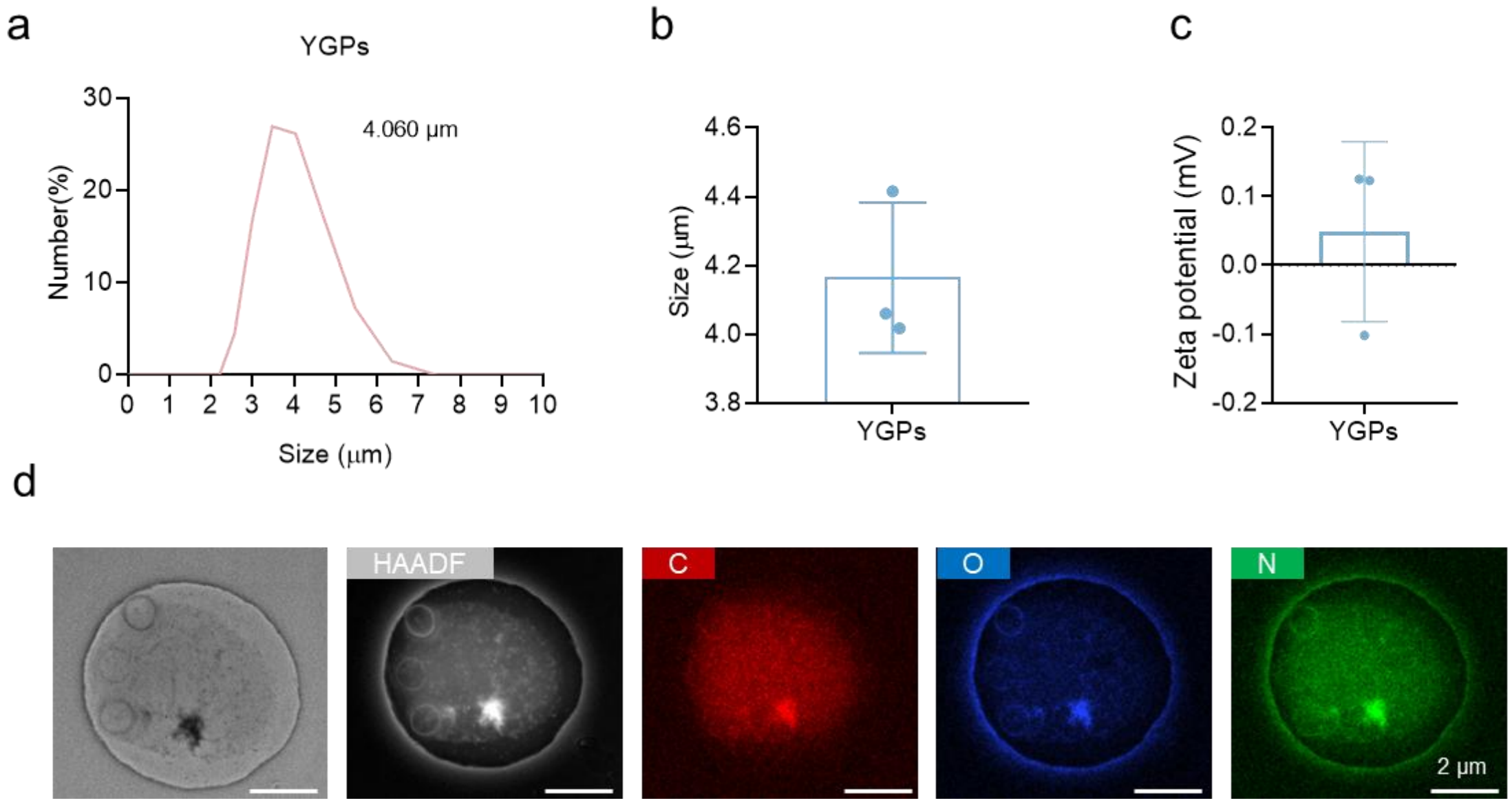

Supplementary Figure 1. Physicochemical characterization of YGPs. (**a**) Size distribution of YGPs measured by DLS. (**b**) Size of YGPs measured by DLS (n = 3 independent samples). (**c**) Zeta potential of YGPs measured by DLS (n = 3 independent samples). (**d**) TEM and EDS elemental mapping images of YGPs. In (d), data are from three independent experiments.

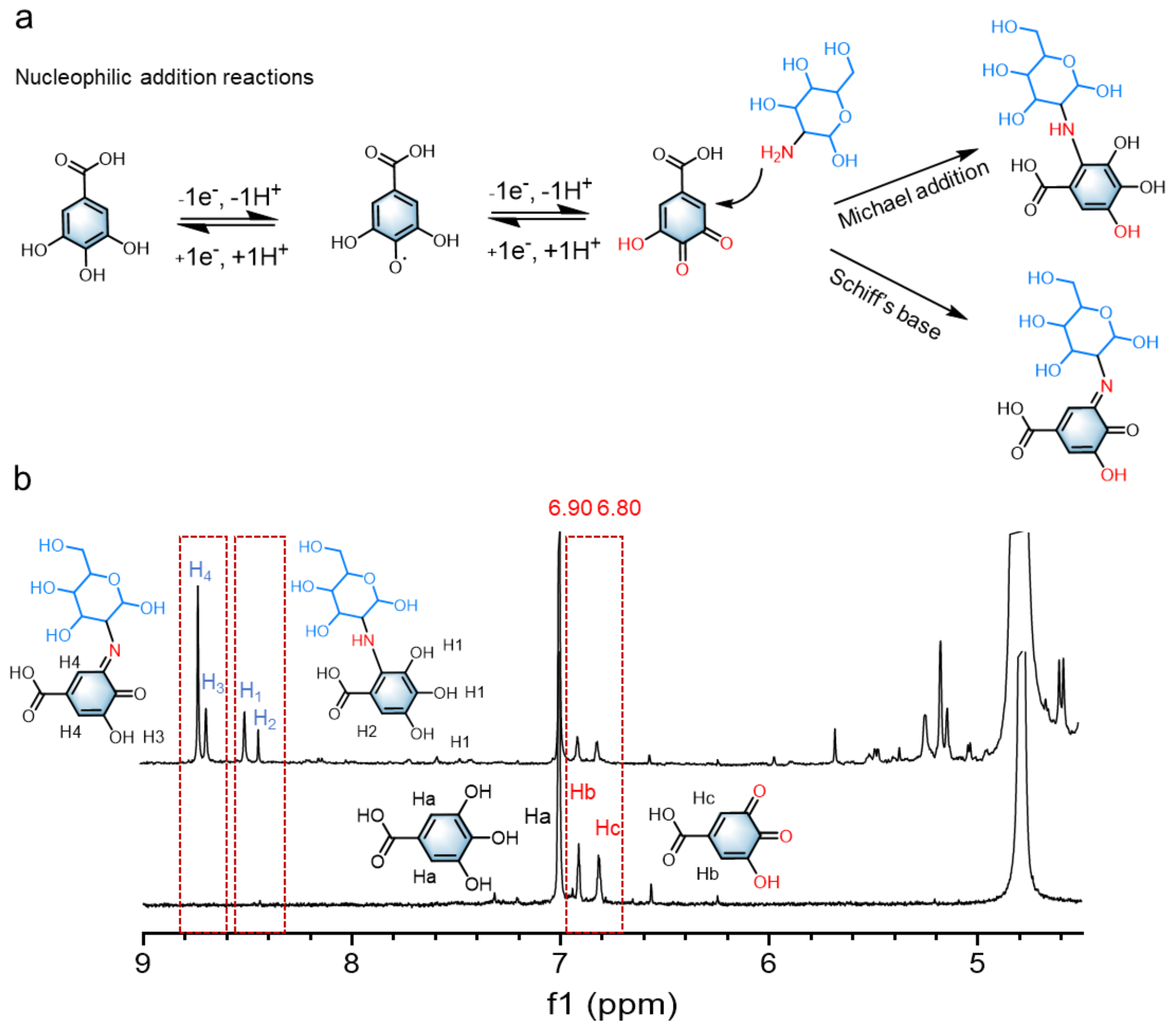

Supplementary Figure 2. Schematic illustration and ¹H NMR verification of the polyphenol-YGPs reaction. (**a**) Schematic diagram of the reaction process between polyphenols and YGPs. (**b**) ¹H NMR spectroscopy of model reactants measured in D_2O. The red dashed rectangles highlight the characteristic proton signals

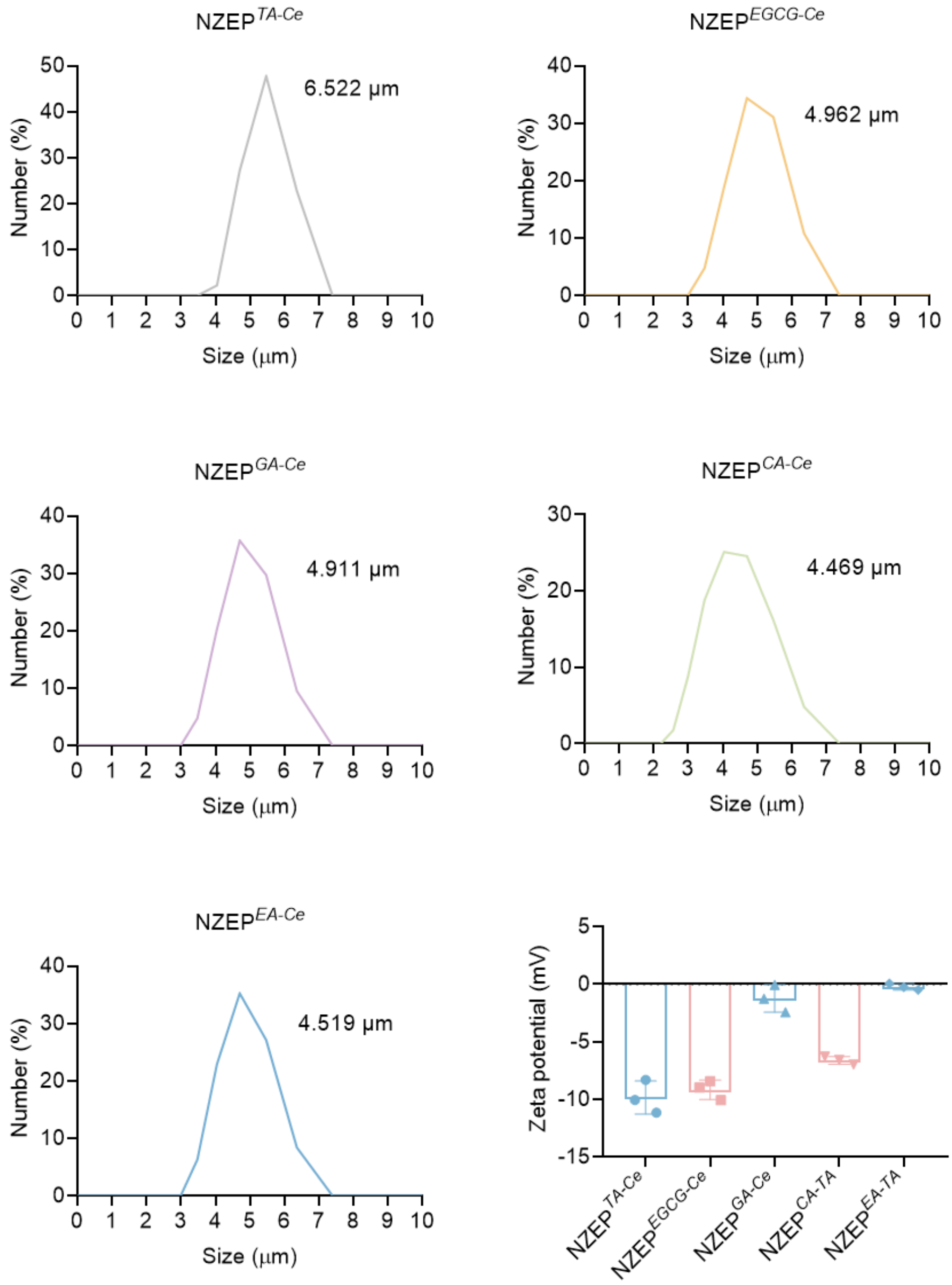

Supplementary Figure 3. Size distribution, expressed as hydrodynamic diameter (n = 3 independent samples) and Zeta potential of NZEP^{MPN-Ce} measured by DLS.

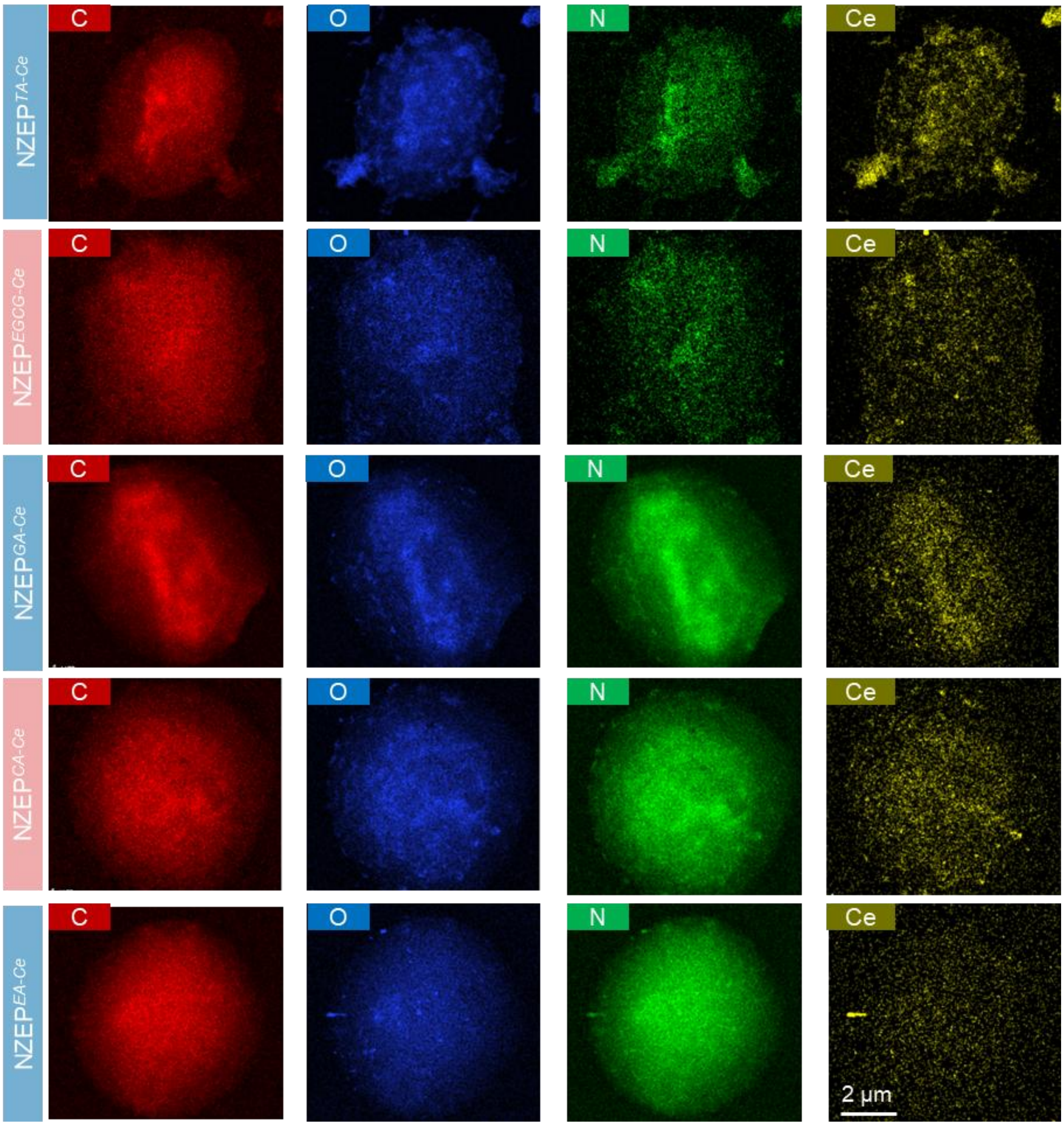

Supplementary Figure 4. EDS elemental mapping images of NZEP$^{MPN\text{-}Ce}$. Data are from three independent experiments.

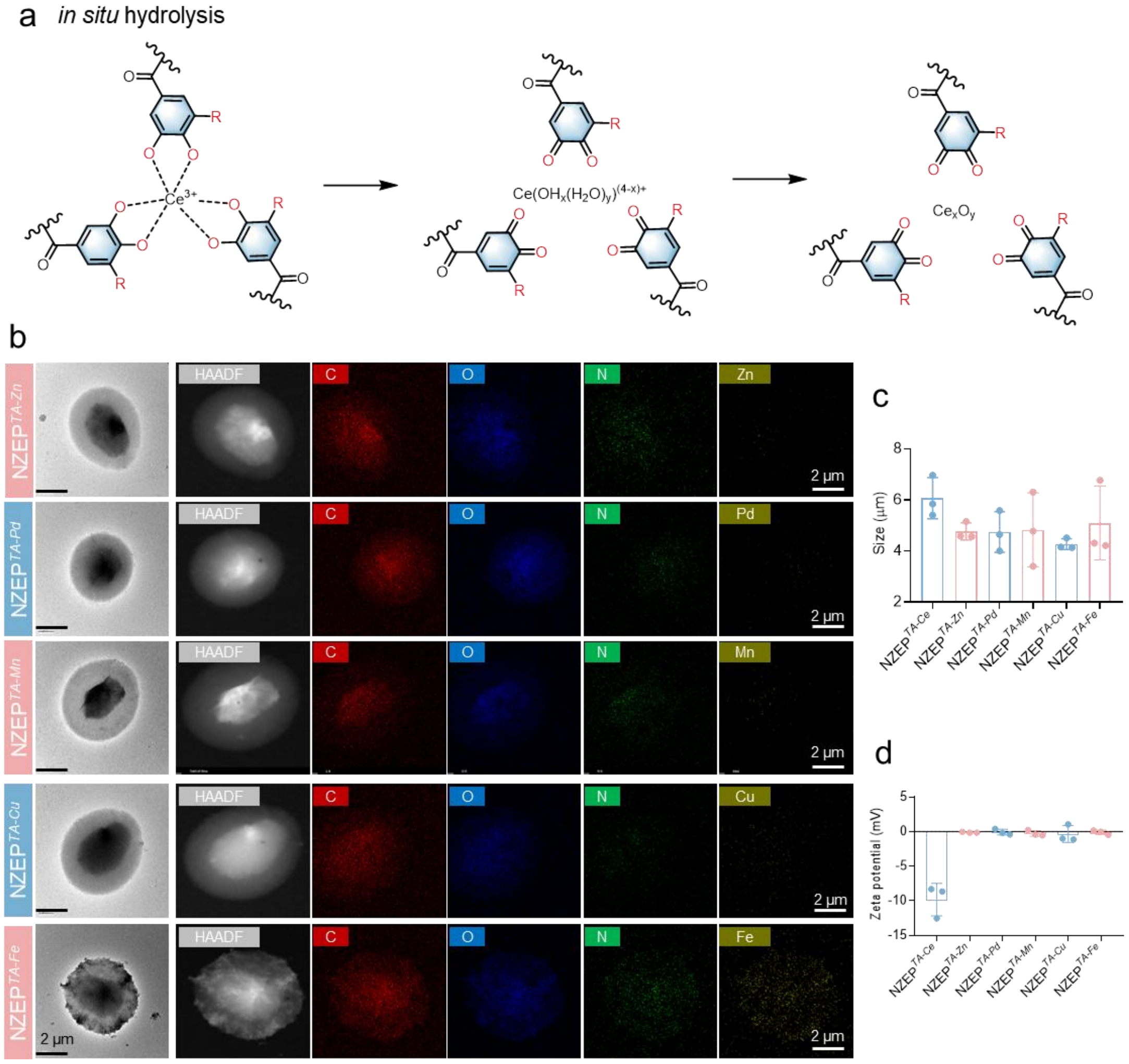

Supplementary Figure 5. Schematic illustration and physicochemical characterization of NZEP$^{TA\text{-}M}$. (**a**) Schematic diagram of the principle of cerium oxide formation by in situ hydrolysis. (**b**) TEM, HAADF and EDS elemental mapping images of NZEP$^{TA\text{-}M}$. (**c**) Size distribution, expressed as hydrodynamic diameter and (**d**) Zeta potential of NZEP$^{TA\text{-}M}$ measured by DLS (n = 3 independent samples). In (b), data are from three independent experiments.

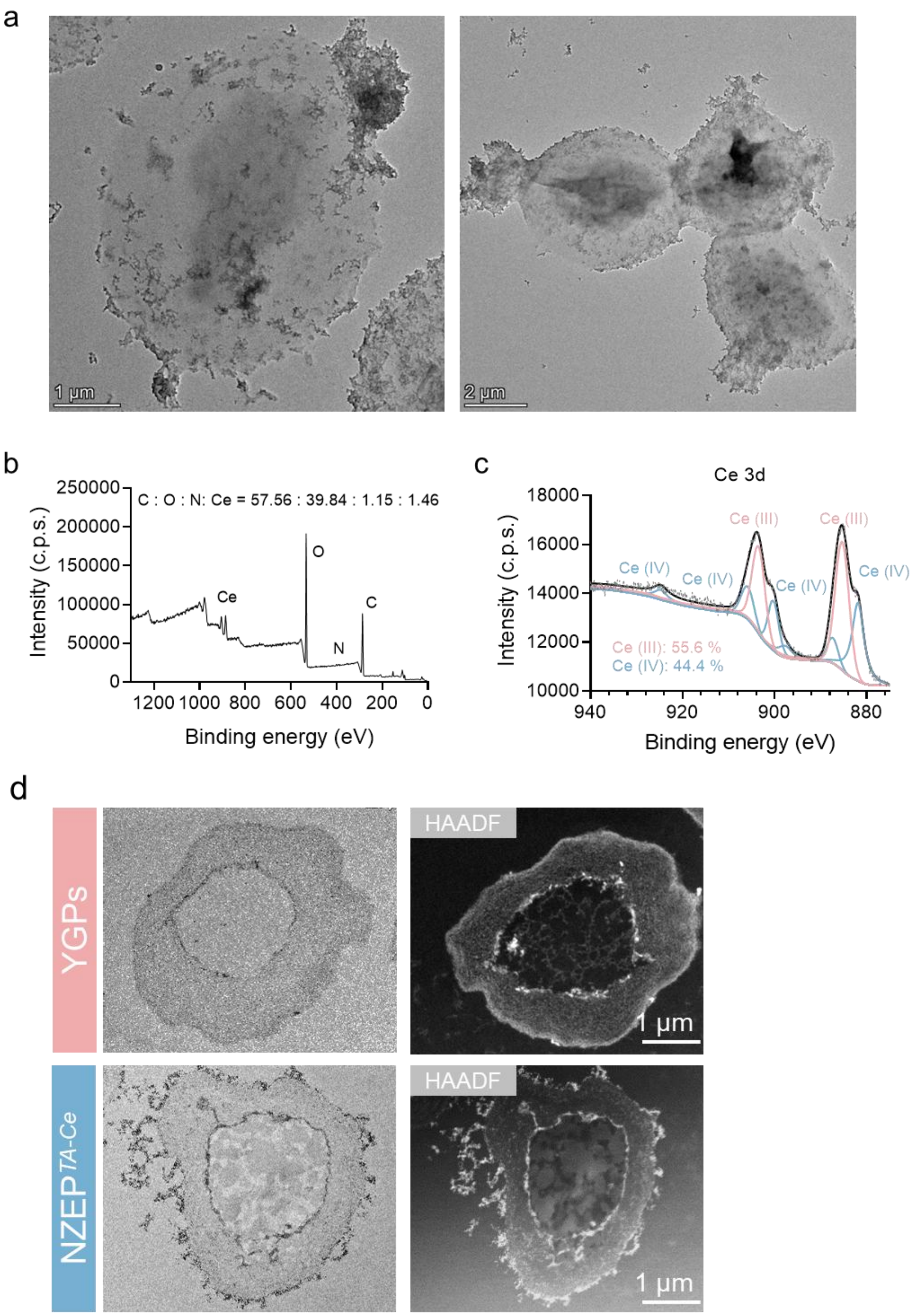

Supplementary Figure 6. Structural and compositional characterization of NZEP$^{TA\text{-}Ce}$. (a) TEM images of the NZEP$^{TA\text{-}Ce}$. (**b**) XPS result of NZEP$^{TA\text{-}Ce}$. (**c**) Ce *3d* XPS spectrum of NZEP$^{TA\text{-}Ce}$. (**d**) TEM and HAADF images of YGPs and NZEP$^{TA\text{-}Ce}$ cross-sections. In (a) and (d), data are from three independent experiments.

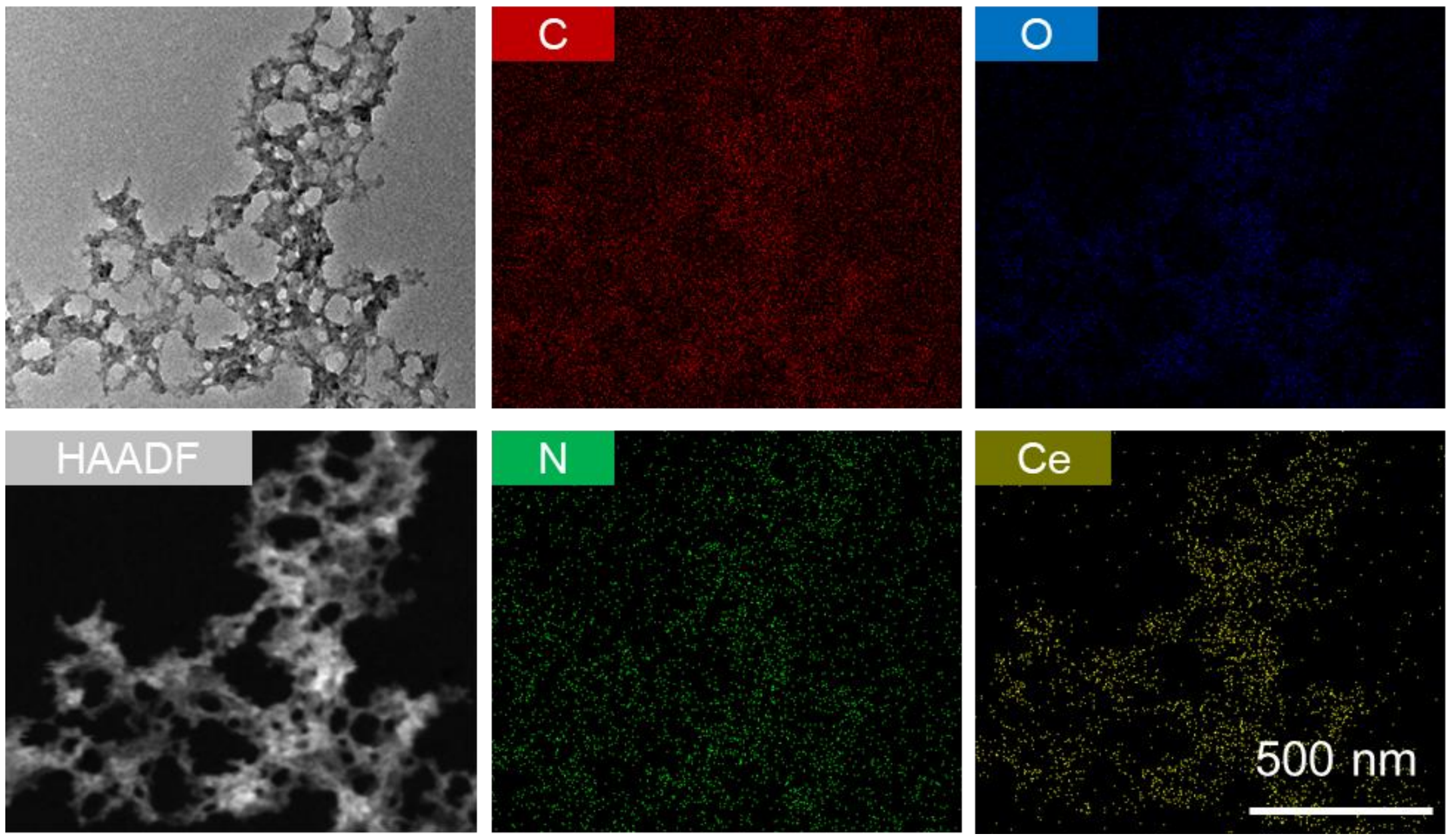

Supplementary Figure 7. TEM and EDS elemental mapping images of TA-Ce. Data are from three independent experiments.

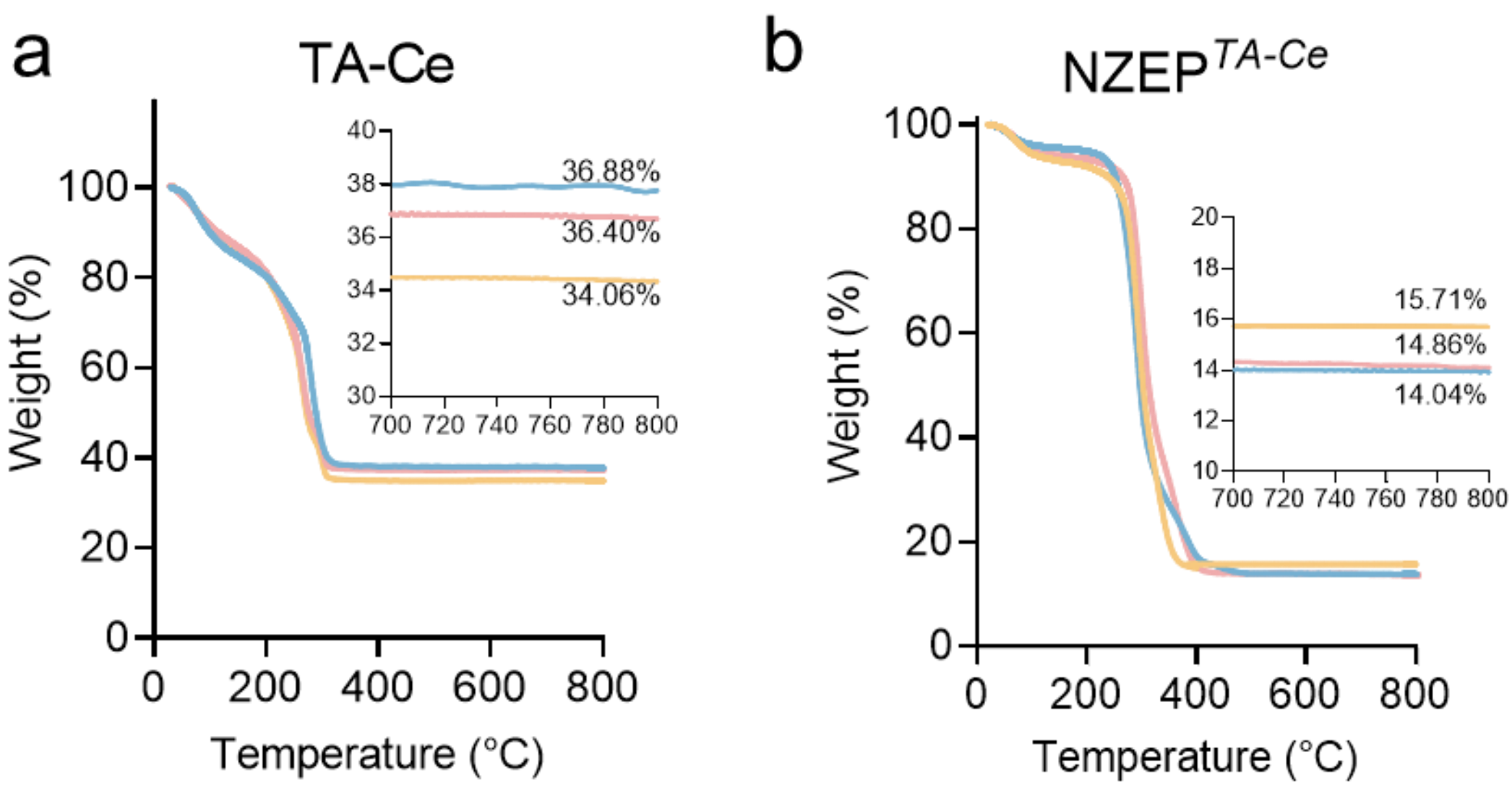

Supplementary Figure 8. Thermogravimetric analysis test of (a) TA-Ce and (b) NZEP$^{TA\text{-}Ce}$.

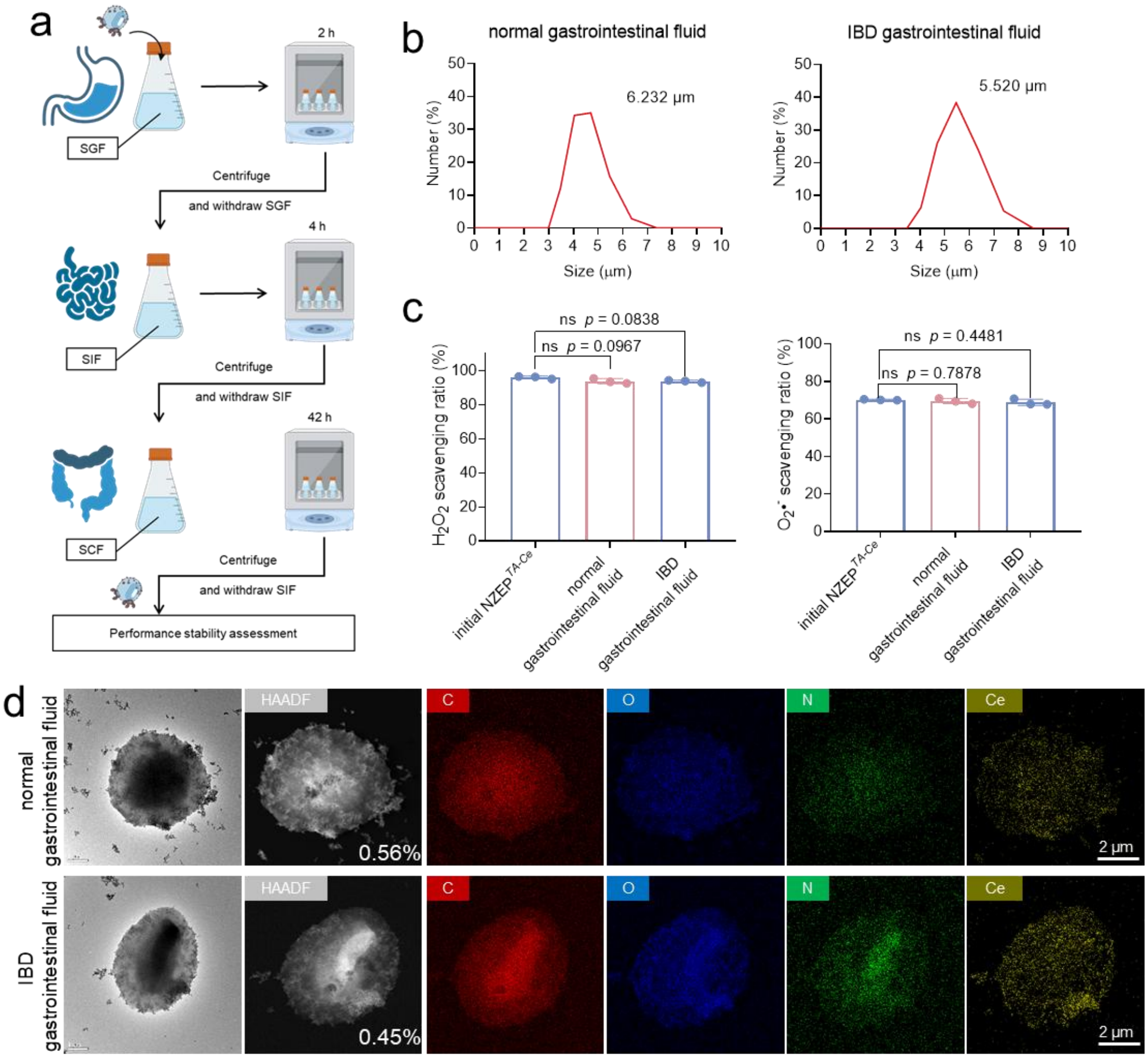

Supplementary Figure 9. Stability of NZEP$^{TA\text{-}Ce}$ in simulated gastrointestinal fluids. (**a**) Schematic diagram of the process (created in BioRender. Zhang, F. (2026) https://BioRender.com/gmhyo8b). (**b**) Size distribution of NZEP$^{TA\text{-}Ce}$ measured by DLS, expressed as hydrodynamic diameter (n = 3 independent samples). (**c**) The ability of the NZEP$^{TA\text{-}Ce}$ to scavenge hydrogen peroxide dnd superoxide anion (n = 3 independent samples). (**d**) TEM and EDS elemental mapping images. Three independent experiments were performed and representative results are shown.

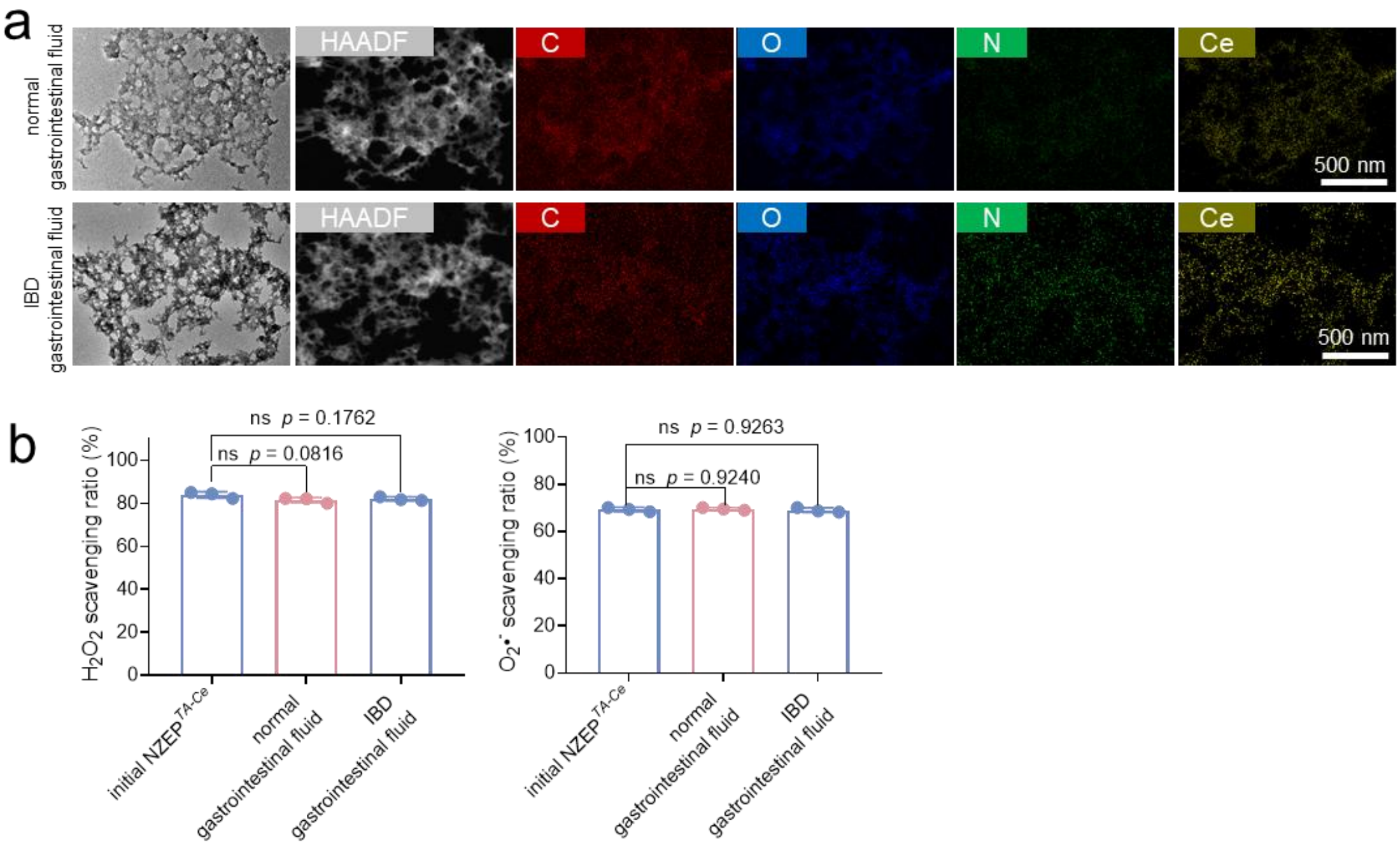

Supplementary Figure 10. Stability of TA-Ce in simulated gastrointestinal fluids. (**a**) TEM and EDS elemental mapping images. Three independent experiments were performed and representative results are shown. (**b**) The ability of TA-Ce to scavenge hydrogen peroxide and superoxide anion (n = 3 independent samples). Data are presented as mean ± SD and statistical significance was analyzed via one-way ANOVA with Tukey's multiple comparison test. *$p < 0.05$, **$p < 0.01$, ***$p < 0.001$, ****$p < 0.0001$.

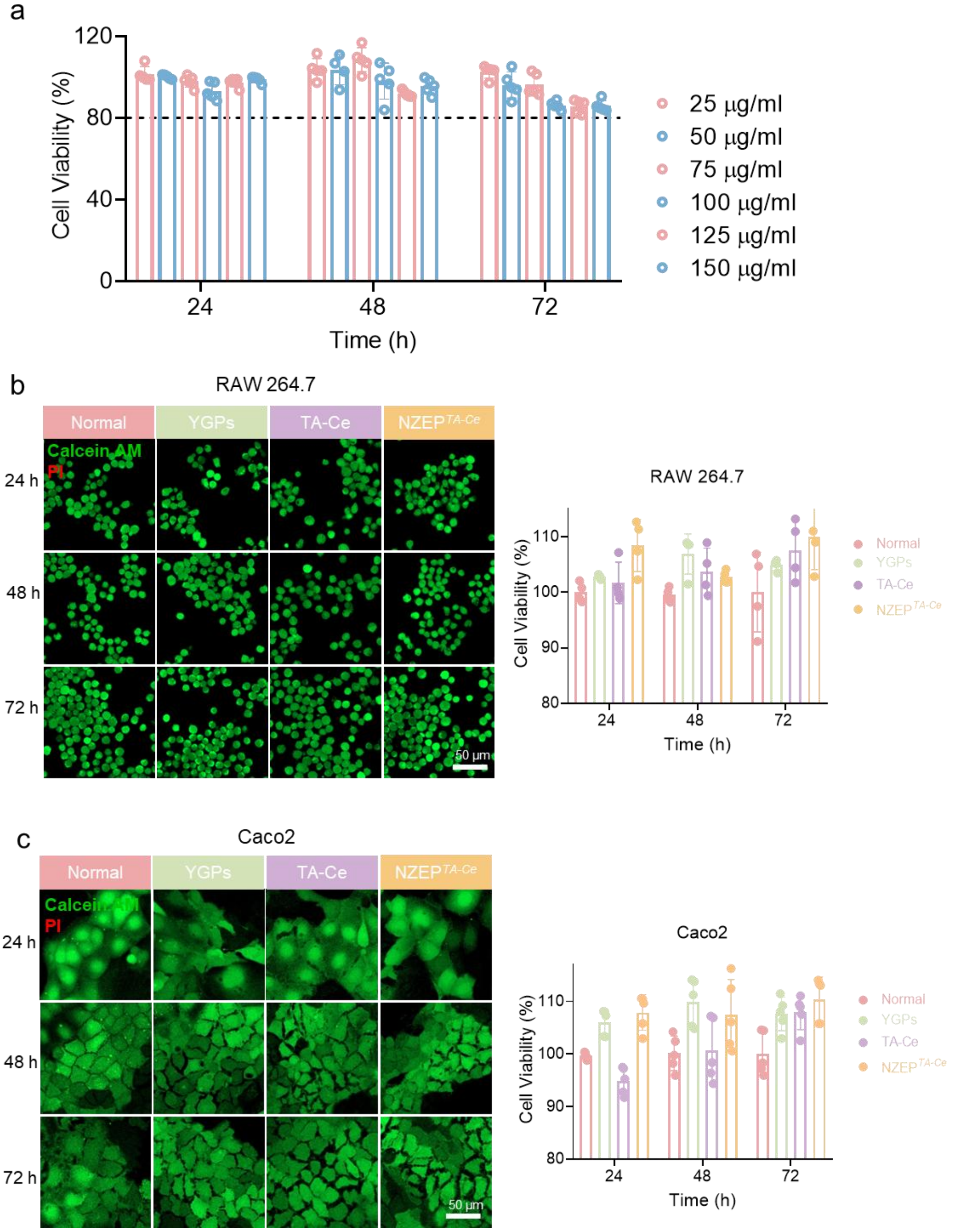

Supplementary Figure 11. ***In vitro*** **cytocompatibility evaluation in RAW264.7 and Caco-2 cells.** (**a**) RAW264.7 cell viability (n = 5 biologically independent samples, data are presented as mean ± SD). (**b**)Live/dead staining images and cell viability of RAW264.7 cells (n = 4 biologically independent samples, data are presented as mean ± SD). (**c**) Live/dead staining images and cell viability of Caco2 cells (n = 5 biologically independent samples, data are presented as mean ± SD). In (b) and (c), data are from three independent experiments.

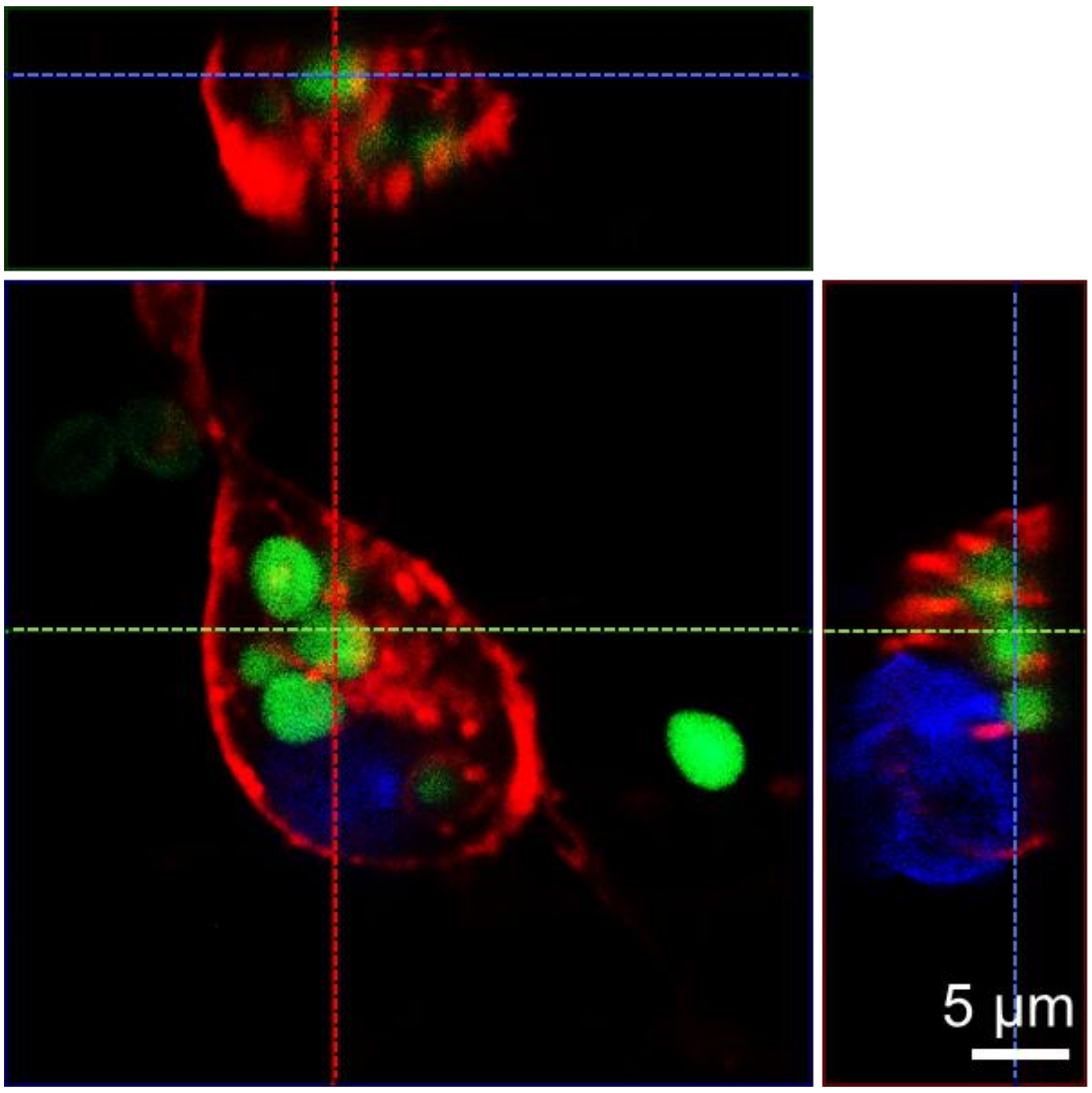

Supplementary Figure 12. Three-Plane sectional imaging of FITC-NZEP$^{TA\text{-}Ce}$ uptake in RAW264.7 cells. Data are from three independent experiments.

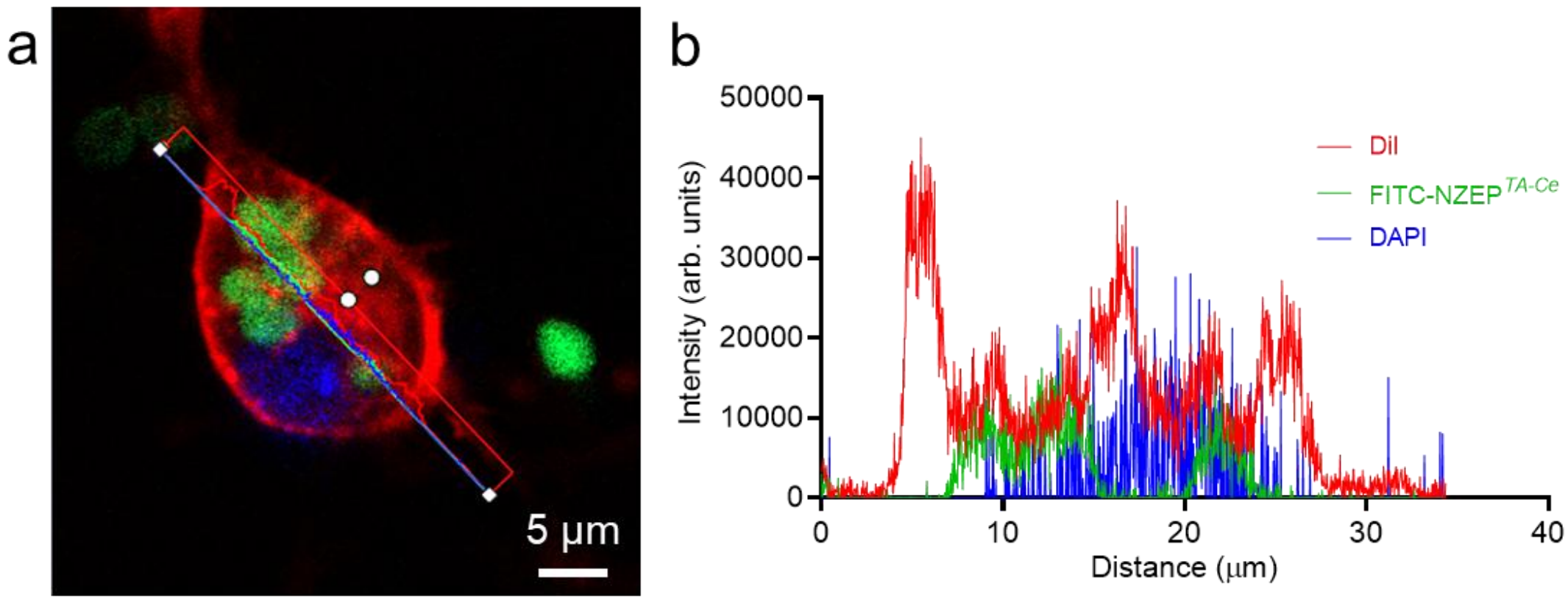

Supplementary Figure 13. Fluorescence colocalization (a) Representative image showing colocalization of Dil, FITC-NZEP$^{TA\text{-}Ce}$ and DAPI in RAW264.7 cells. Data are from three independent experiments. (b) Quantitative analysis of fluorescence colocalization.

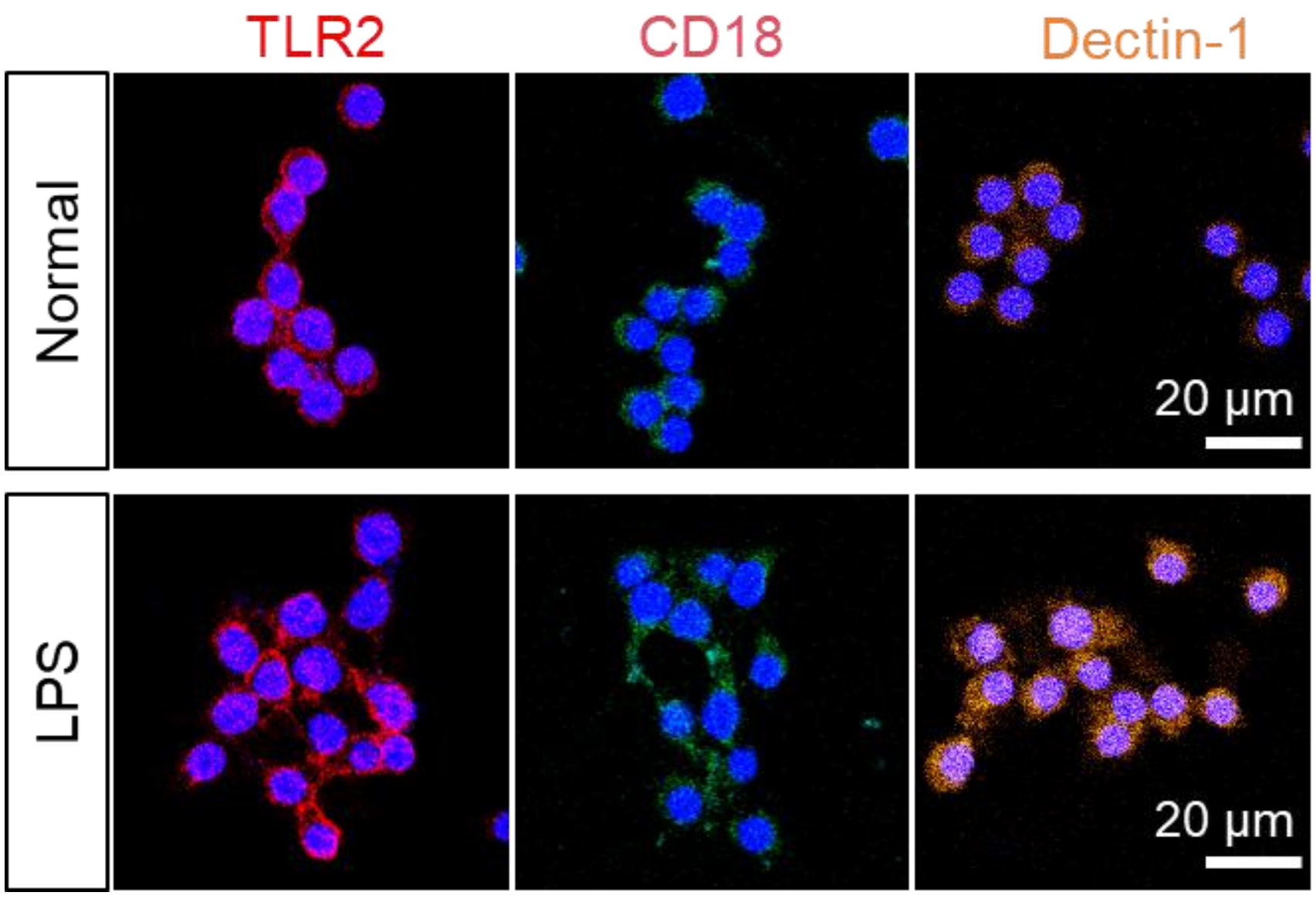

Supplementary Figure 14. Representative immunofluorescence-stained image of glucan-specific receptors on the surface of RAW264.7 cells. Data are from three independent experiments.

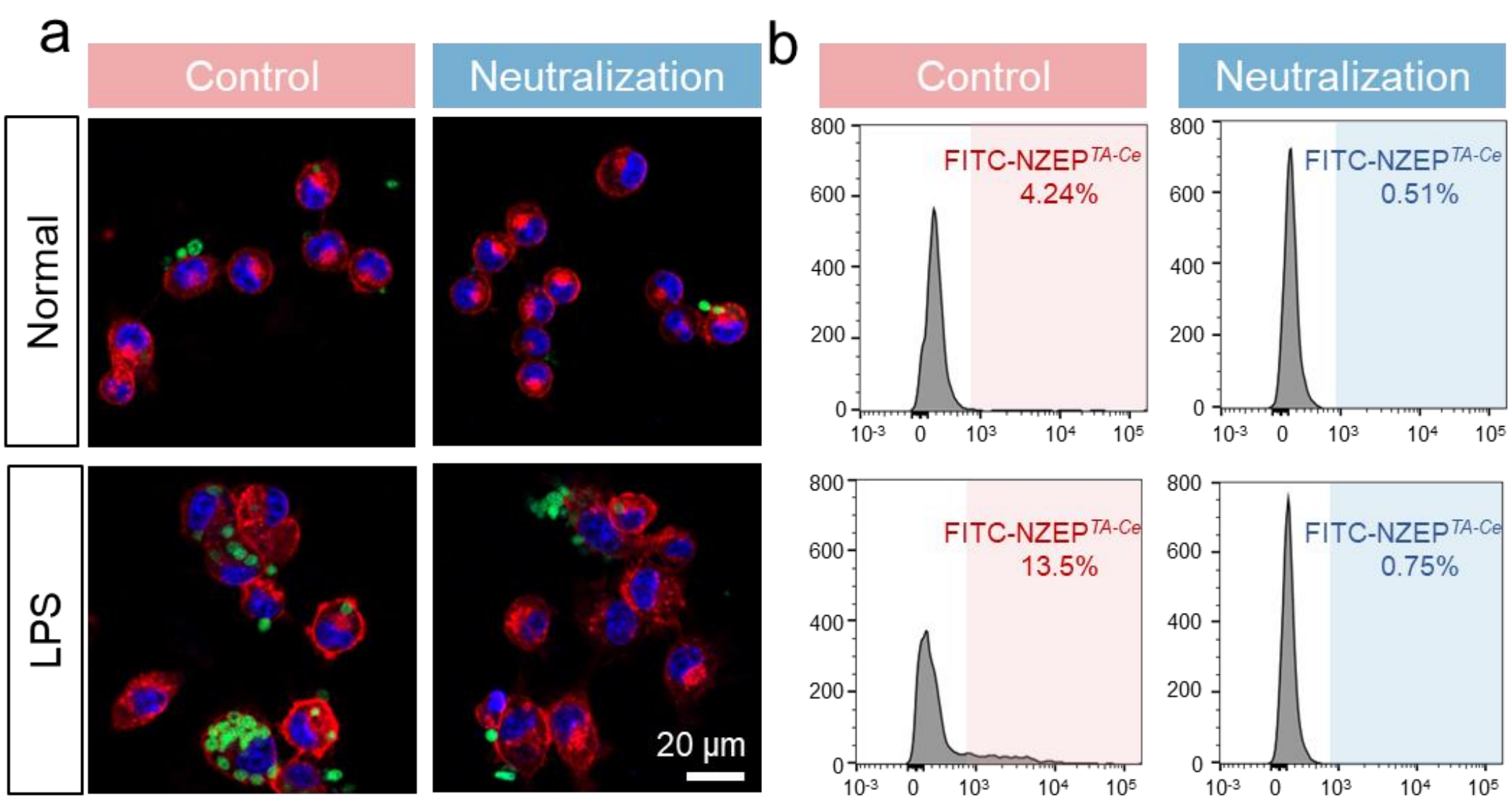

Supplementary Figure 15. Receptor-mediated uptake of NZEP$^{TA\text{-}Ce}$ in RAW264.7 cells. (**a**) Representative image and (**b**) flow cytometry of NZEP$^{TA\text{-}Ce}$ being taken up by RAW264.7 at 12 h with receptor neutralization. Three independent experiments were performed. In (a), data are from three independent experiments.

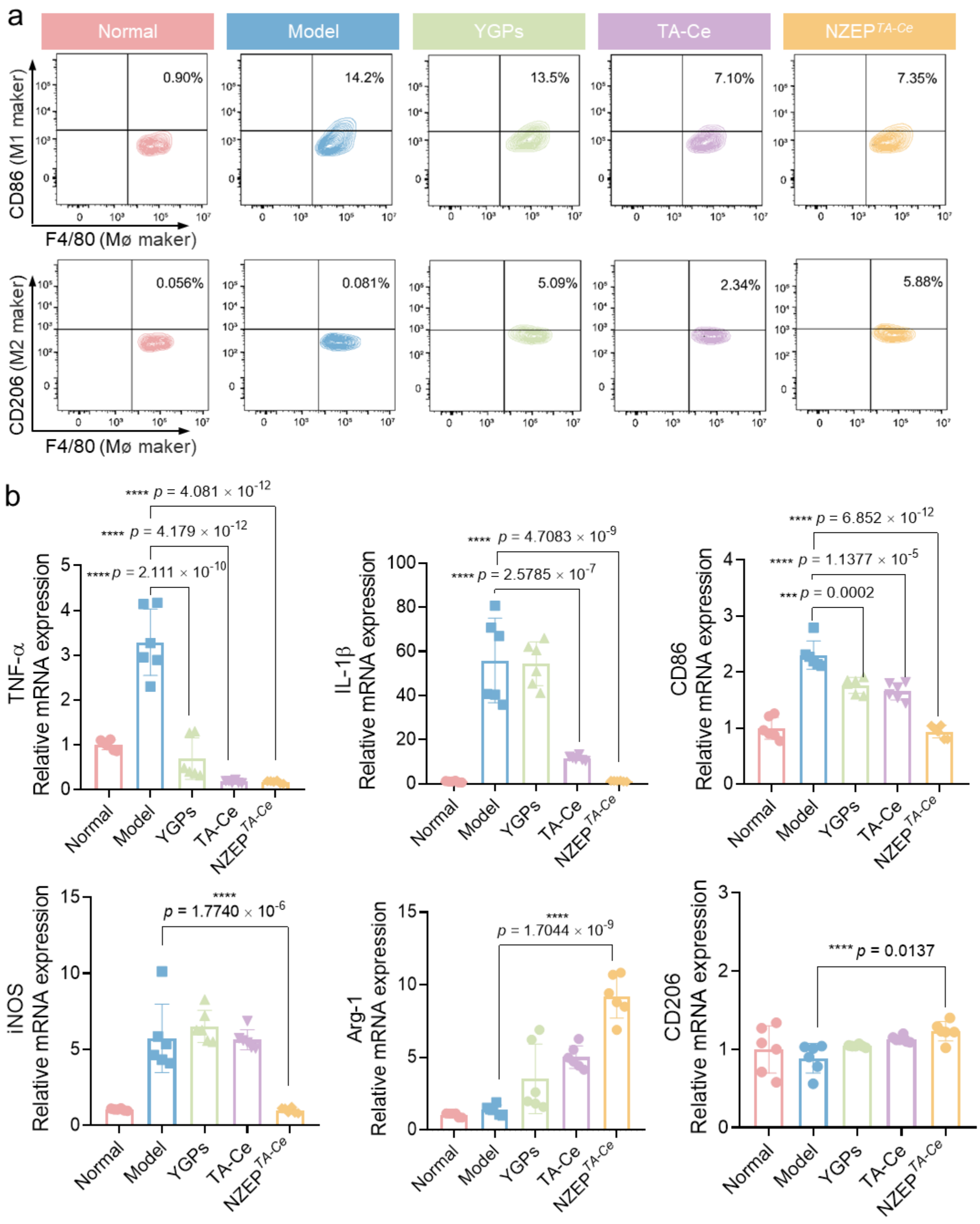

Supplementary Figure 16. Immunomodulatory effects of NZEP*TA-Ce* on macrophage polarization. (**a**) Representative flow cytometry of CD86 and CD206 fluorescence in RAW264.7 cells. (**b**) Relative mRNA expression of pro-inflammatory (*Tnf-α*, *Il-1β*, *iNOS* and *Cd86*) and anti-inflammatory factors (*Arg-1*, *Cd206*) Three independent experiments were performed. Data are presented as mean ± SD and statistical significance was analyzed via one-way ANOVA with Tukey's multiple comparison test. *$p < 0.05$, **$p < 0.01$, ***$p < 0.001$, ****$p < 0.0001$.

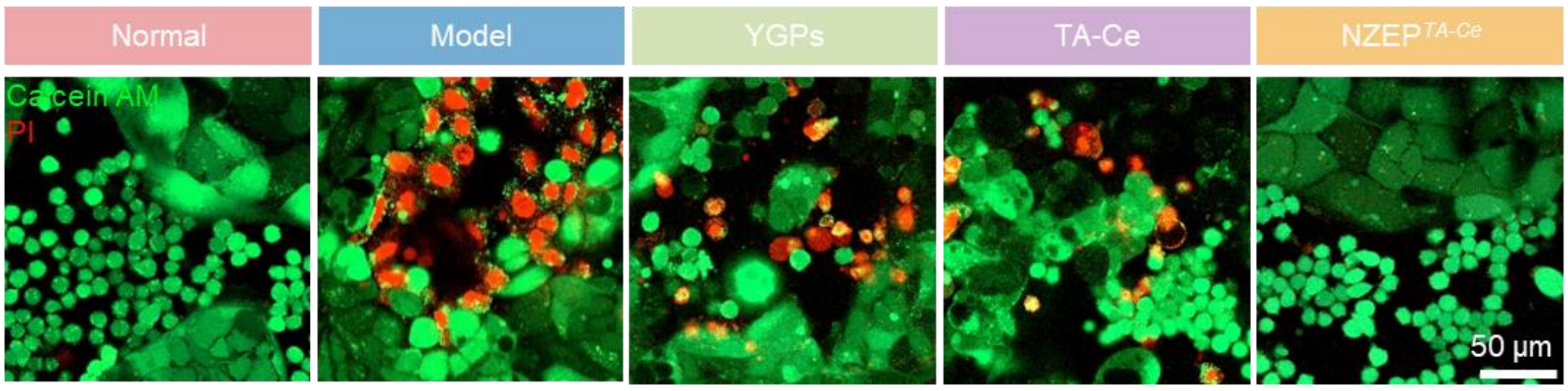

Supplementary Figure 17. Live/dead staining images of co-incubation of RAW264.7 and Caco2 cells. Three independent experiments were performed and representative results are shown.

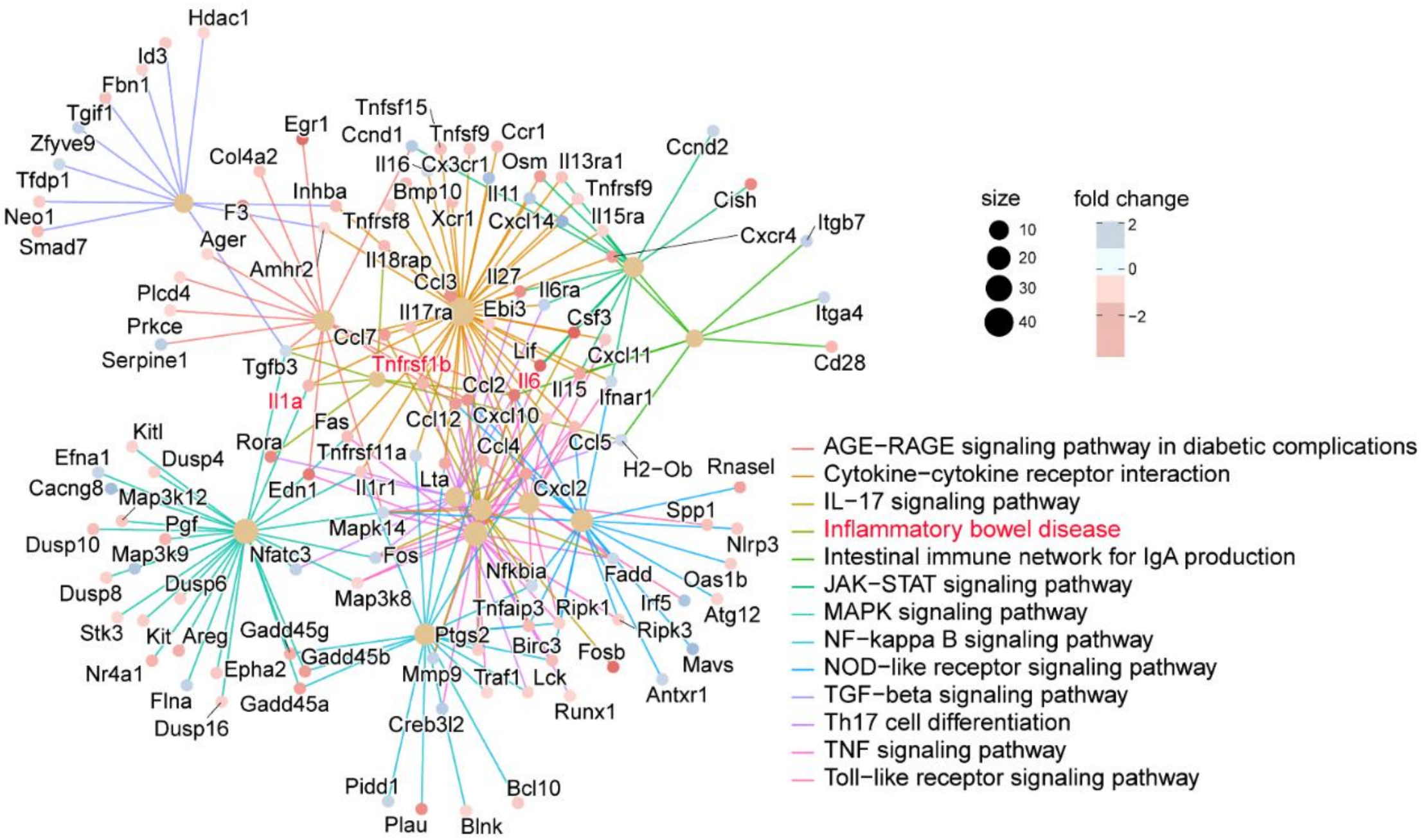

Supplementary Figure 18. KEGG signaling pathway network map.

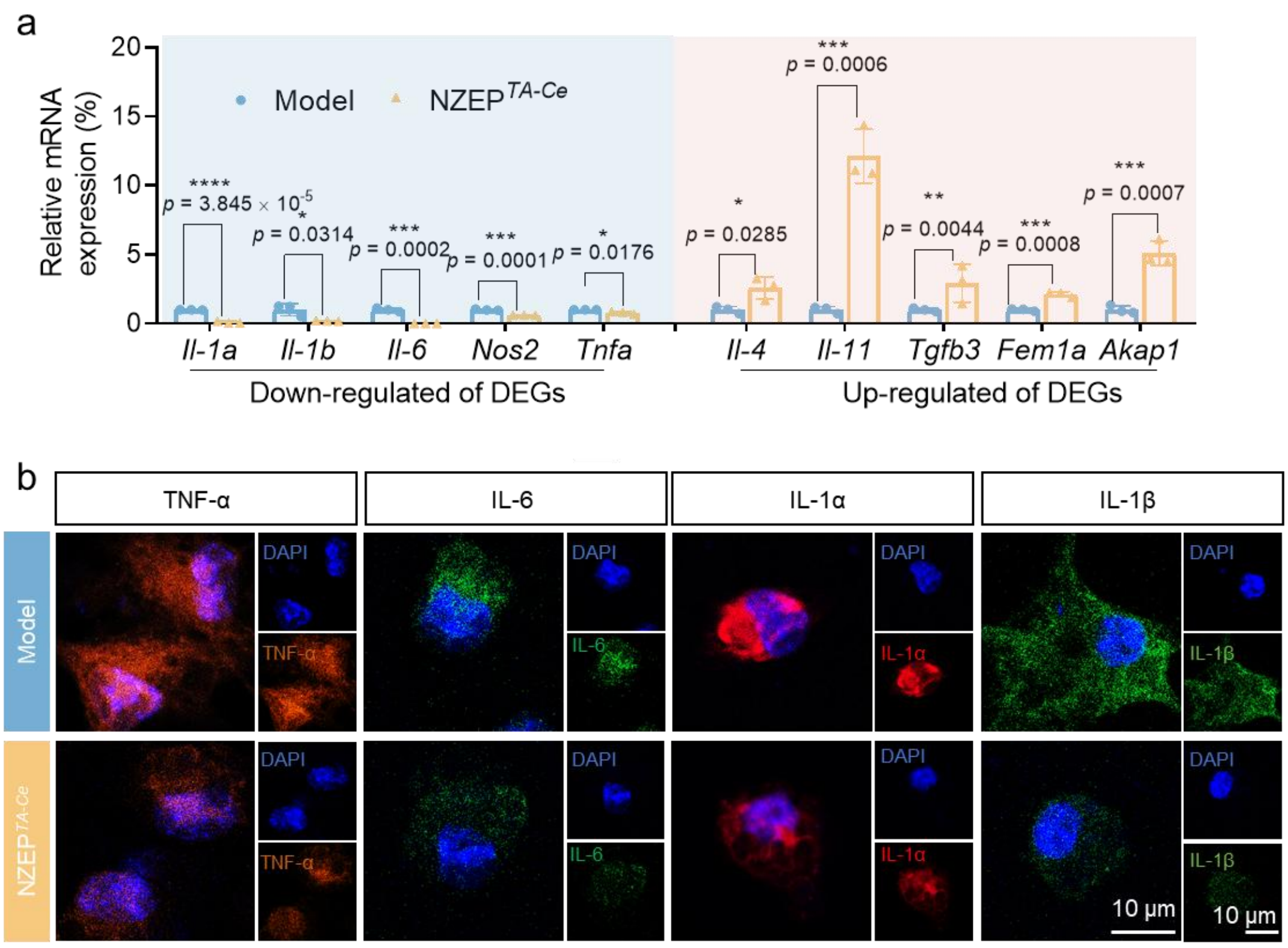

Supplementary Figure 19. Validation of inflammation-related DEGs and cytokine expression in RAW264.7 cells. (**a**) Relative mRNA expression of specific DEGs including *Il-1a*, *Il-1b*, *Il-6*, *Nos2*, *Tnfa*, *Il-4*, *Il-11*, *Tgfb3*, *Fem1a* and *Akap1*. (**b**) Representative immunofluorescence staining for TNF-α, IL-6, IL-1α and IL-1β. In (a), n = 3 independent experiments. Three independent experiments were performed and representative results are shown in (b). Data are presented as mean ± SD and statistical significance was analyzed via one-way ANOVA with Tukey's multiple comparison test. *$p < 0.05$, **$p < 0.01$, ***$p < 0.001$, ****$p < 0.0001$.

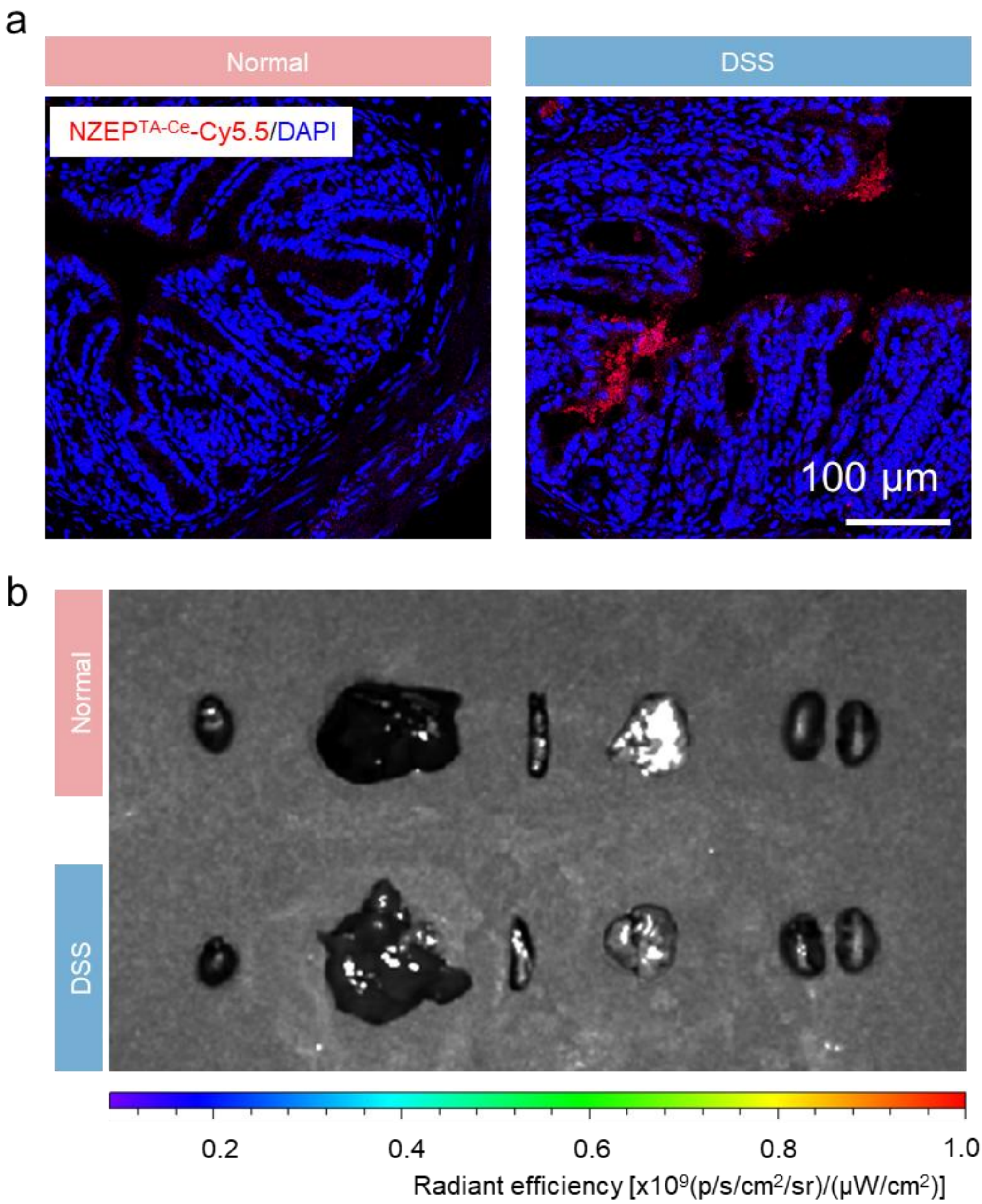

Supplementary Figure 20. **Biodistribution and tissue fluorescence imaging in normal and colitis mice.** (**a**) Fluorescence staining of colon tissue from normal and colitis mice at the endpoint (n = 3 mice per group). (**b**) Fluorescence images of major organs (heart, liver, spleen, lungs and kidneys) from normal and colitis mice at the endpoint (n = 3 mice per group). Three independent experiments were performed and representative results are shown.

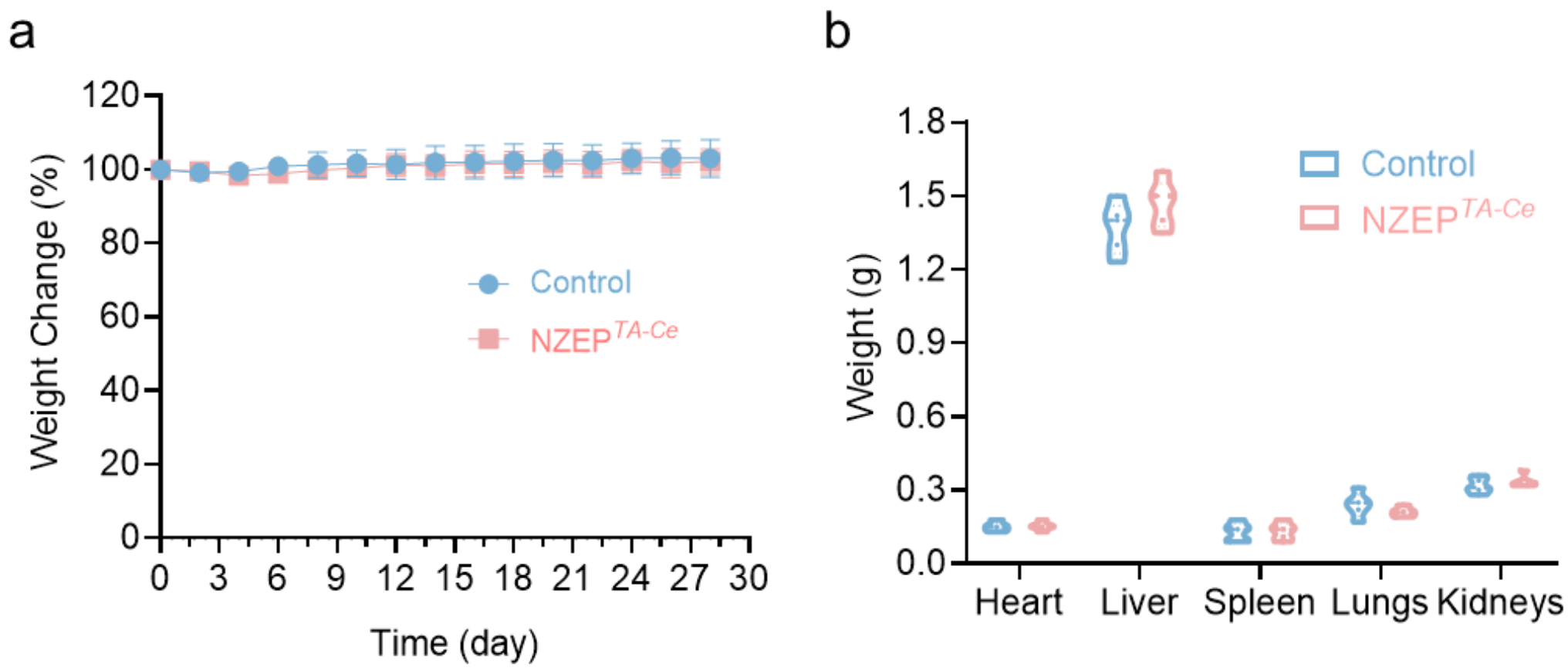

Supplementary Figure 21. *In vivo* biosafety evaluation. (**a**) The weight changes of mice during the experiment (n = 5 mice). (**b**) Weights of major organs after 30 days of continuous gavage (n = 5 mice).

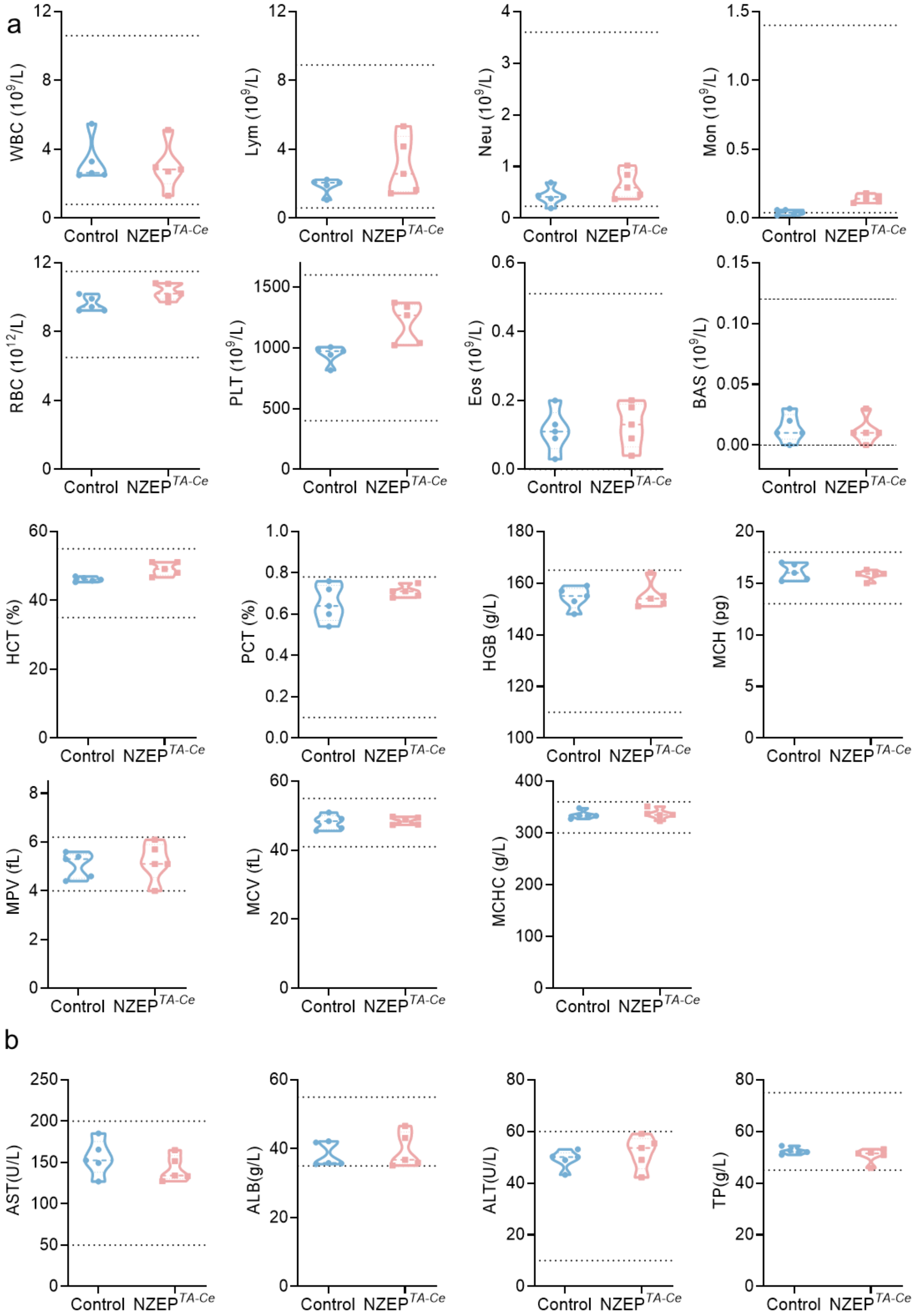

Supplementary Figure 22. Hematological and serum biochemical analysis for *in vivo* biosafety evaluation. (**a**) blood routine index (n = 5 mice) (**b**) blood biochemical index (n = 5 mice).

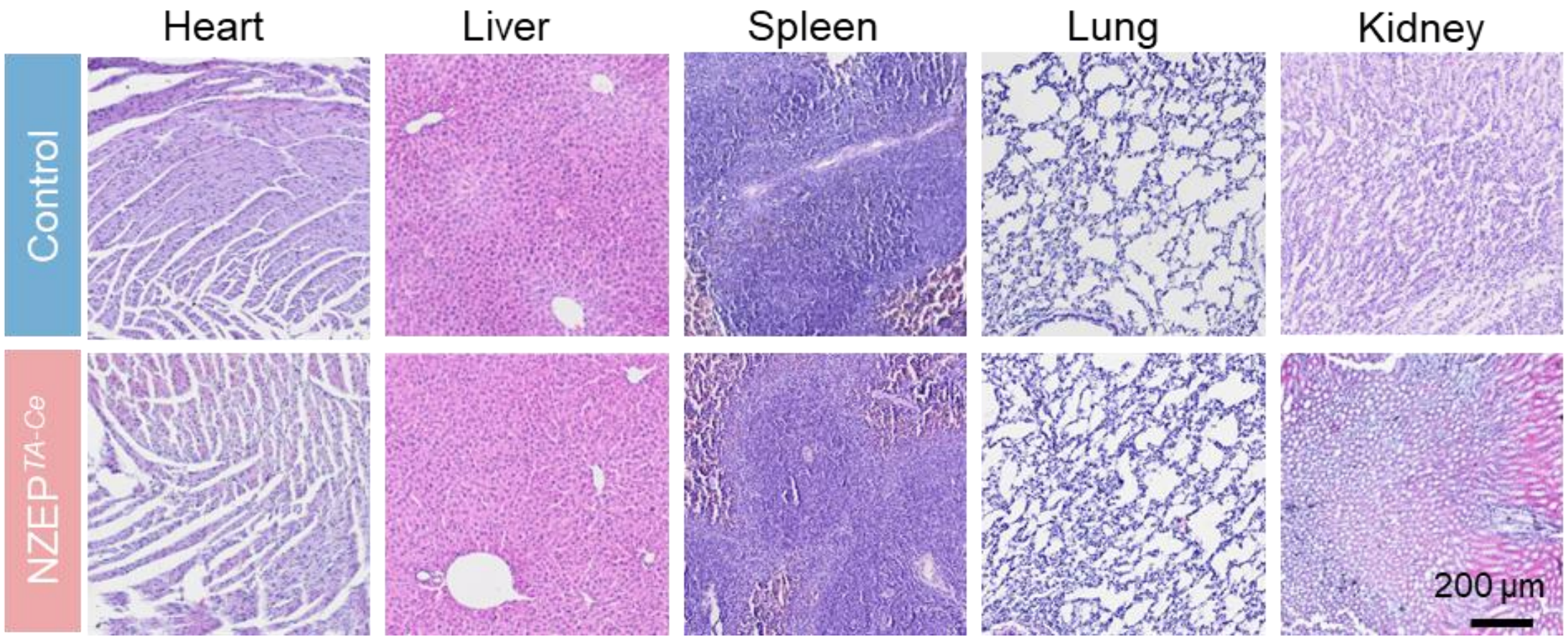

Supplementary Figure 23. Representative image of H&E staining of major organs. Three independent experiments were performed and representative results are shown (n = 5 mice).

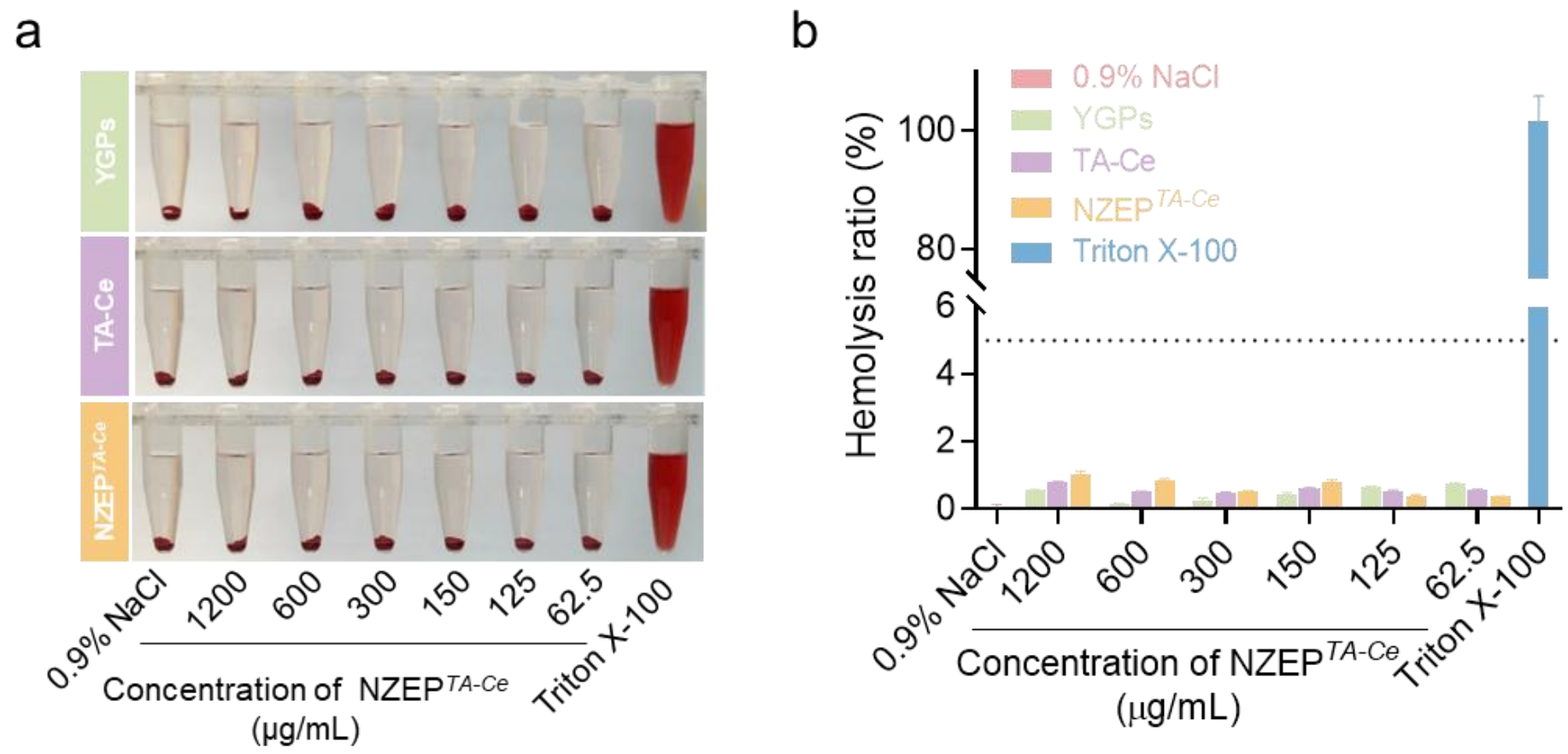

Supplementary Figure 24. Hemocompatibility evaluation of NZEP$^{TA\text{-}Ce}$. (**a**) Representative pictures. Three independent experiments were performed and representative results are shown. (**b**) Hemolysis rate (n = 3 independent blood samples).

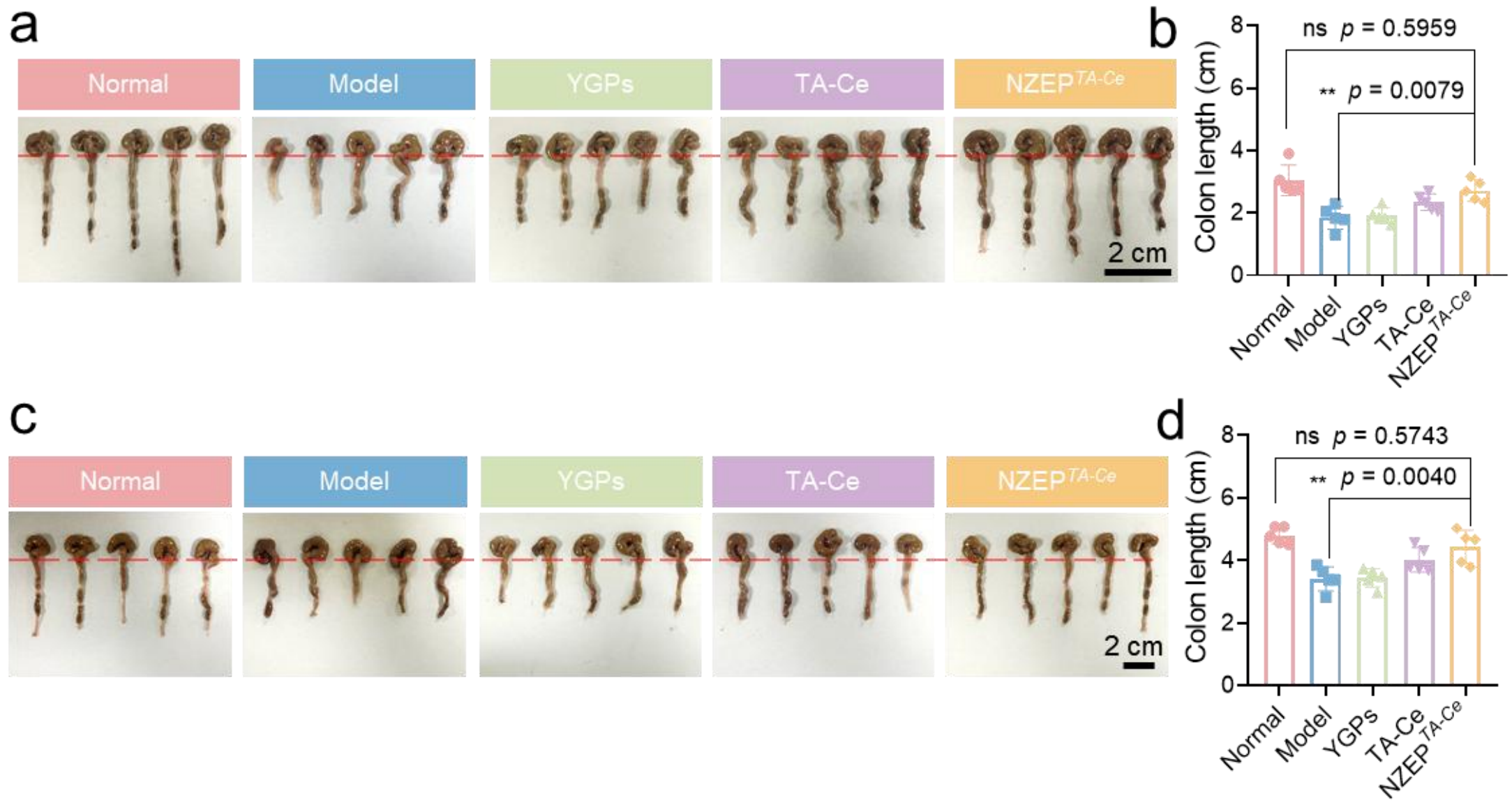

Supplementary Figure 25. Independent experimental validation of therapeutic efficacy in the DSS-induced IBD model. representative colonic images (**a**, **c**) and colon length (**b**, **d**) measurements from two additional independent experiments in the 3 % DSS-induced IBD model (n = 5 mice). Data are presented as mean ± SD and statistical significance was analyzed via one-way ANOVA with Tukey's multiple comparison test. **p* < 0.05, ***p* < 0.01, ****p* < 0.001, *****p* < 0.0001.

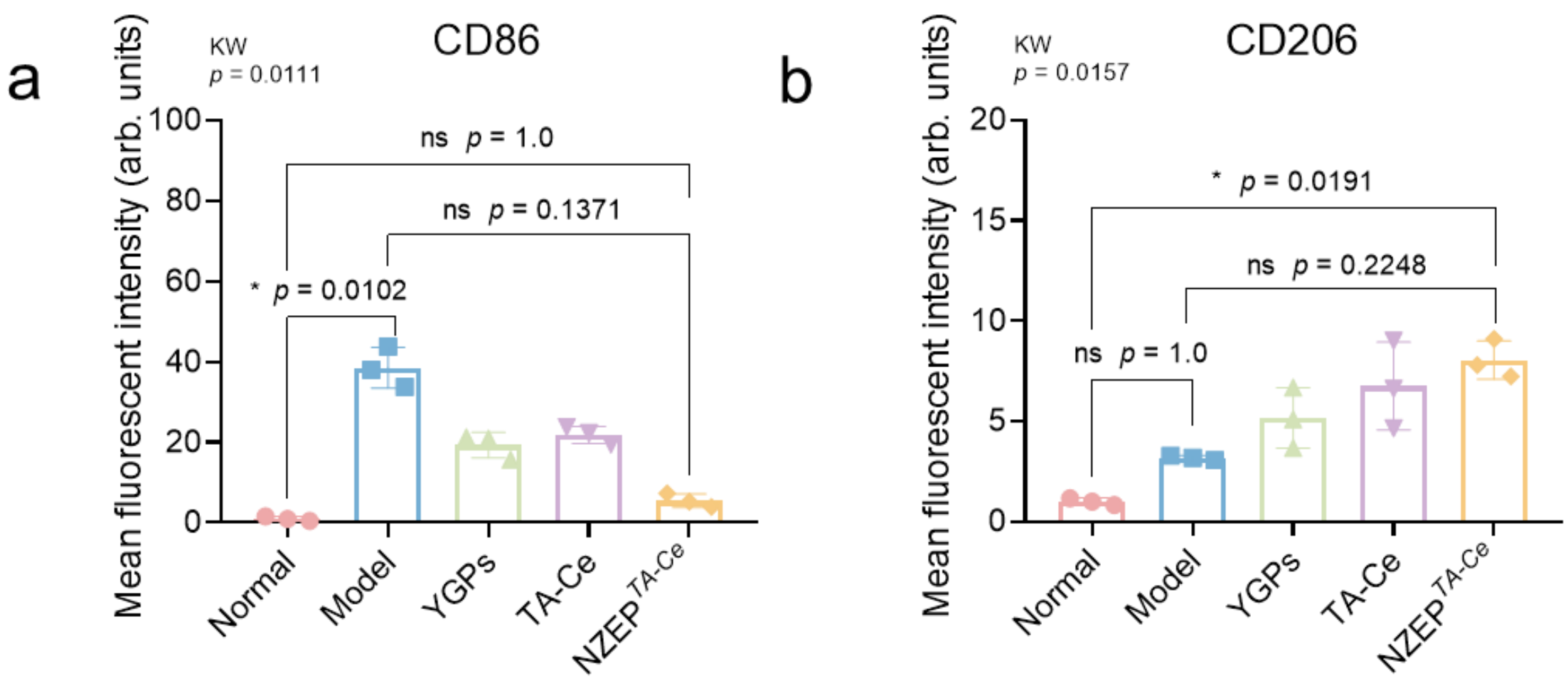

Supplementary Figure 26. Mean fluorescence intensity of CD86 and CD206 in immunofluorescence staining images of colonic tissue. Data are presented as mean ± SD (n = 3 mice per group). Each data point represents one mouse. *p* values were determined using the Kruskal–Wallis test followed by Dunn's multiple comparisons test. Although the individual values of the

Normal and NZEP$^{TA\text{-}Ce}$ groups and those of the Model and NZEP$^{TA\text{-}Ce}$ groups in (a), as well as those of the Normal and Model groups and those of the Model and NZEP$^{TA\text{-}Ce}$ groups in (b), do not overlap, the corresponding pairwise comparisons did not reach statistical significance, possibly due to the limited sample size. KW *p*' represents the overall *p* value obtained from the Kruskal-Wallis test across all experimental groups.

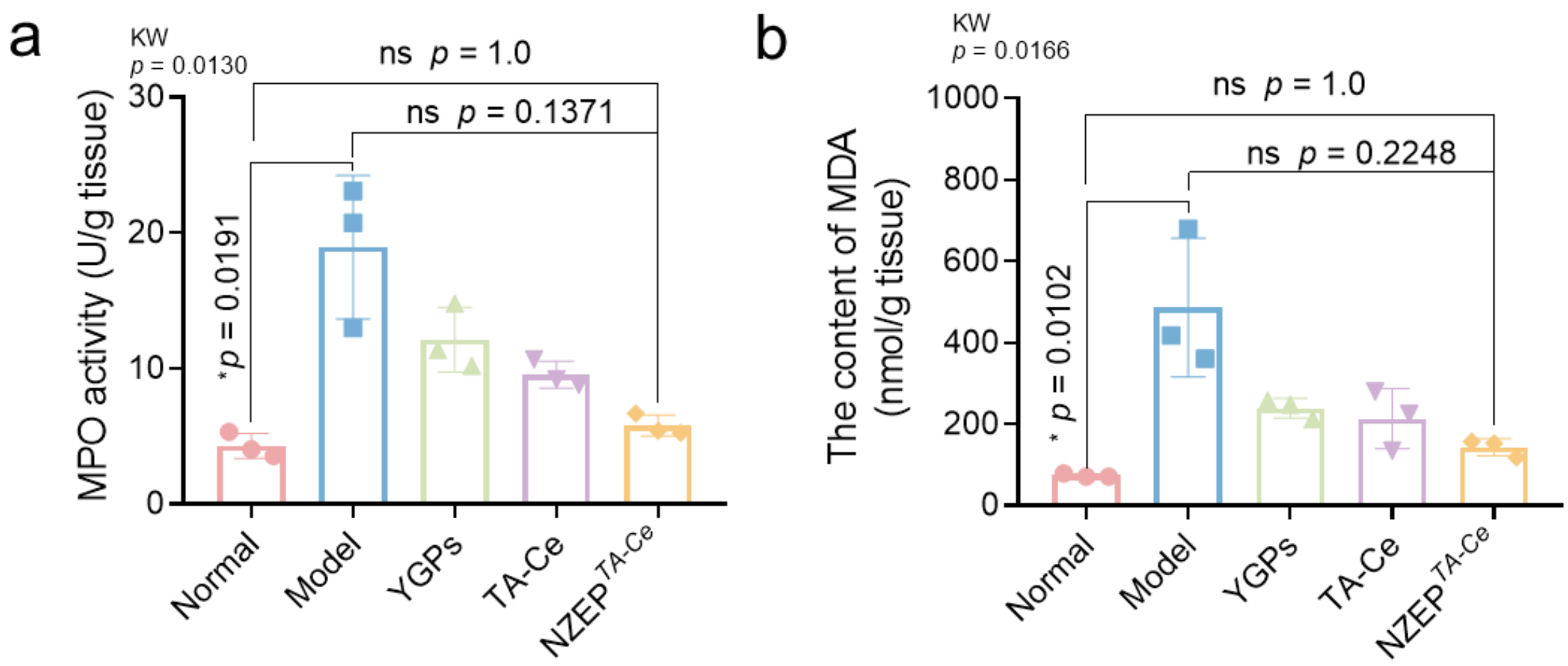

Supplementary Figure 27. Evaluation of oxidative stress and inflammatory activity in colon tissues. (**a**) Relative MPO activity and (**b**) MDA content in colon tissues from different treatment groups. Data are presented as mean ± SD (n = 3 mice per group). Each data point represents one mouse. *p* values were determined using the Kruskal–Wallis test followed by Dunn's multiple comparisons test. Although the individual values of the Model and NZEP$^{TA\text{-}Ce}$ groups in (a), as well as those of the Normal and NZEP$^{TA\text{-}Ce}$ groups and those of the Model and NZEP$^{TA\text{-}Ce}$ groups in (b), do not overlap, the corresponding pairwise comparisons did not reach statistical significance, possibly due to the limited sample size. KW *p*' represents the overall *p* value obtained from the Kruskal-Wallis test across all experimental groups.

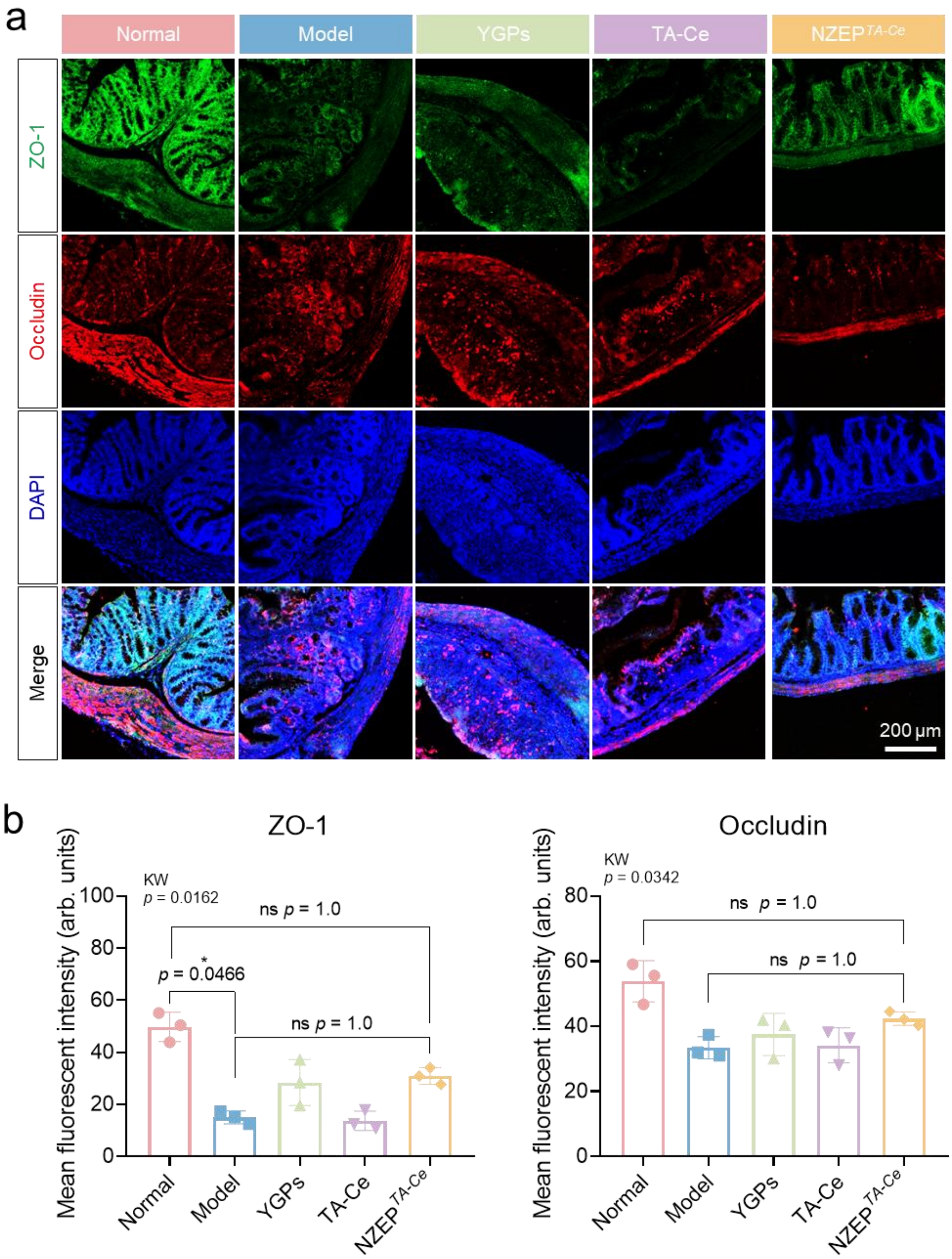

Supplementary Figure 28. Tight junction protein expression in colon tissues. (**a**) Representative immunofluorescence staining images of ZO-1 and occludin. (**b**) Mean fluorescence intensity of ZO-1 and occludin. Data are presented as mean ± SD (n = 3 mice per group). Each data point represents one mouse. *p* values were determined using the Kruskal–Wallis test followed by Dunn's multiple comparisons test. Although the individual values of the Normal and NZEP$^{TA\text{-}Ce}$ groups and those of the Model and NZEP$^{TA\text{-}Ce}$ groups in (b) do not overlap, the corresponding pairwise comparisons did

not reach statistical significance, possibly due to the limited sample size. KW *p*' represents the overall *p* value obtained from the Kruskal-Wallis test across all experimental groups.

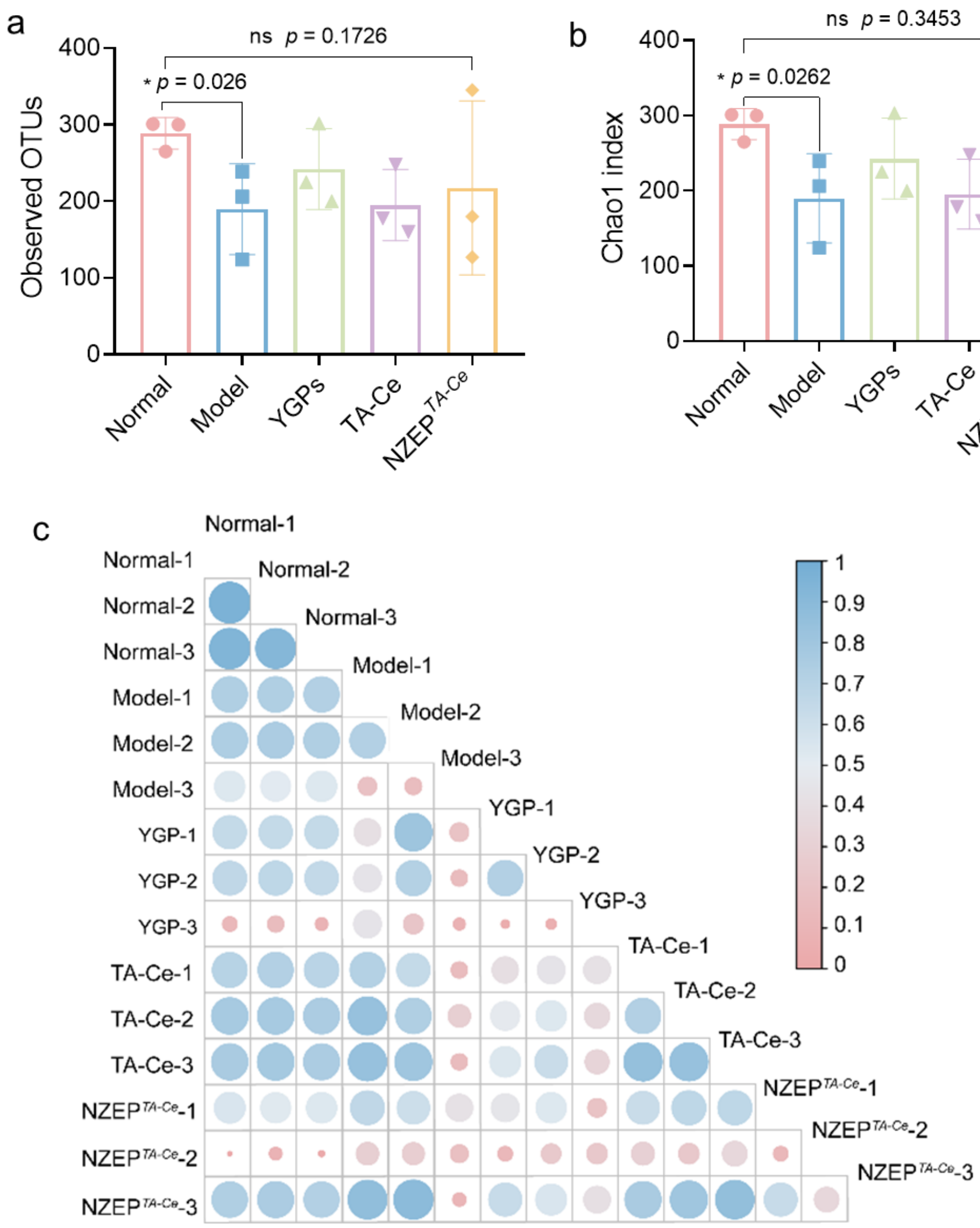

Supplementary Figure 29. Alpha and beta diversity analysis of gut microbiota. (**a**) Observed OTUs (n = 3 mice) (**b**) Chao1 index (n = 3 mice). (**c**) β-Diversity index analysis based on the unweighted unifrac distance matrix heatmap. Data are presented as mean ± SD and statistical significance was analyzed via one-way ANOVA with Tukey's multiple comparison test. *$p < 0.05$, **$p < 0.01$, ***$p < 0.001$, ****$p < 0.0001$.

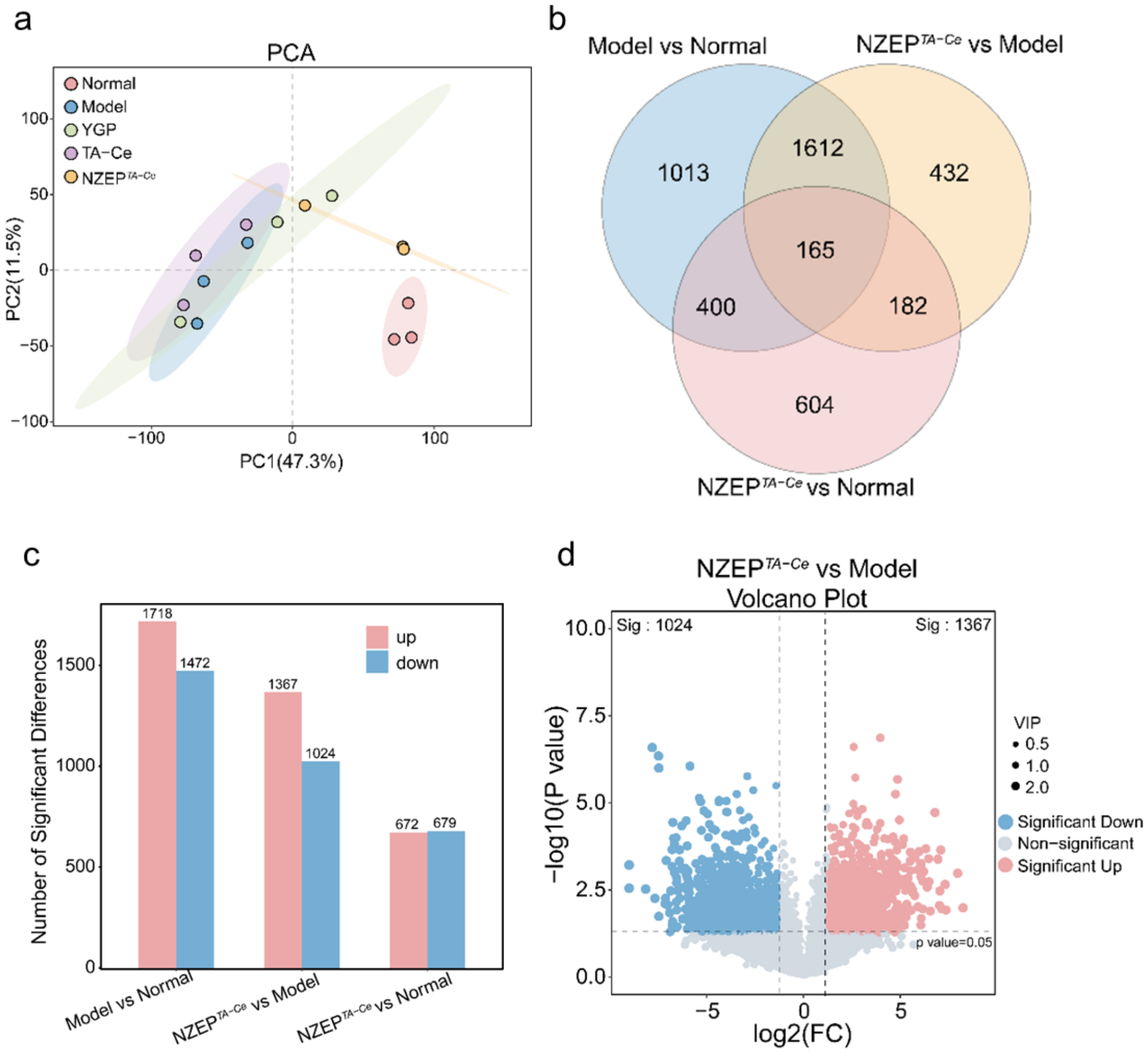

Supplementary Figure 30. Integrated analysis of gut microbiota composition and differential metabolite profiles. (**a**) Principal components analysis (PCA) of the gut microbiome, each point represents one mouse. (**b**) Venn diagram of the number of gut microbiome in Normal, Model and NZEP$^{TA\text{-}Ce}$ group. (**c**) Histogram of the number of gut microbiome in Normal, Model and NZEP$^{TA\text{-}Ce}$ group. (**d**) Volcano plot of the differential metabolites, wherein each point represents a metabolite. Significantly up-regulated metabolites are indicated by red points and significantly down-regulated metabolites are indicated by blue points.

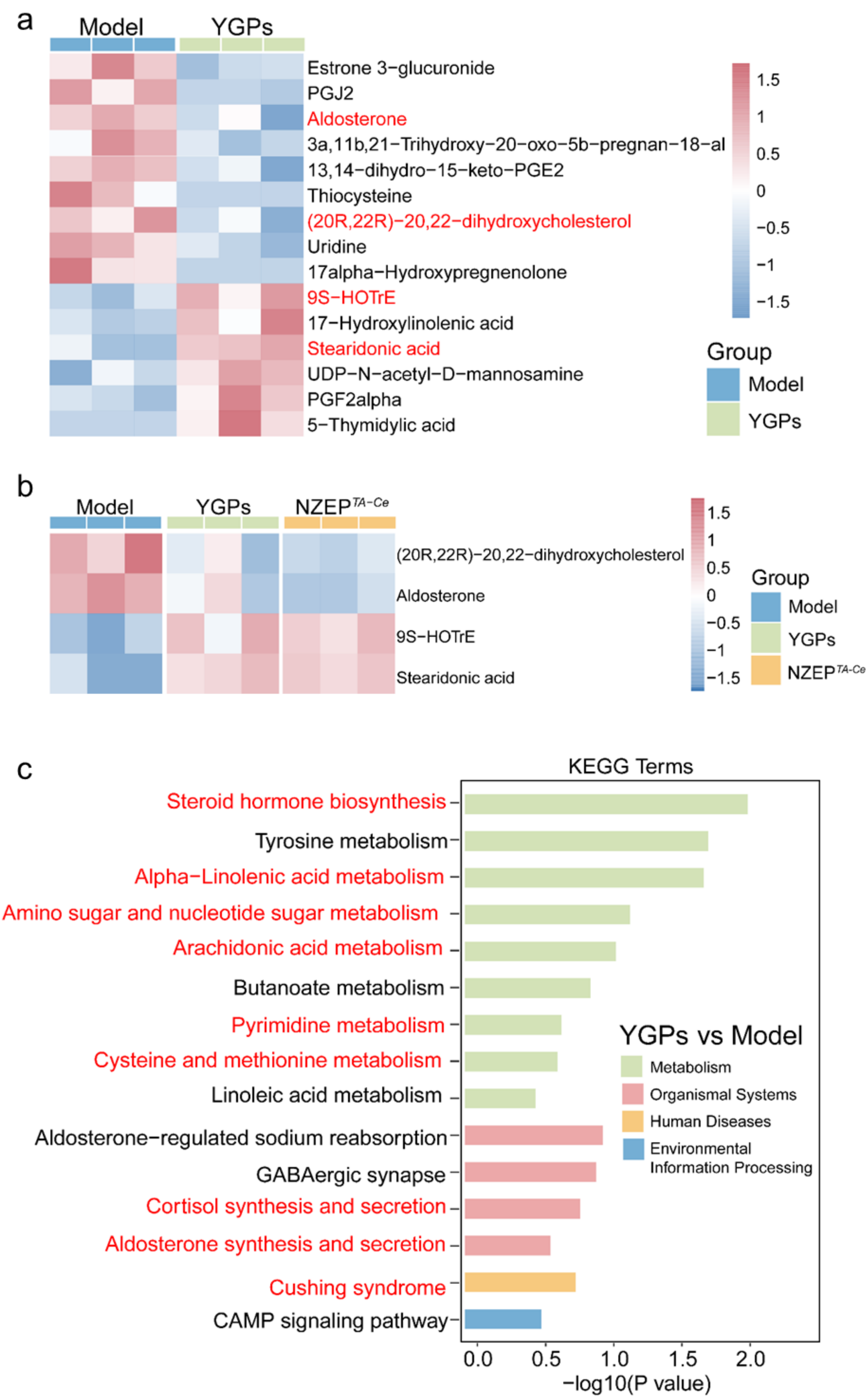
a
Model
YGPs
Estrone 3−glucuronide
PGJ2
Aldosterone
3a,11b,21−Trihydroxy−20−oxo−5b−pregnan−18−al
13,14−dihydro−15−keto−PGE2
Thiocysteine
(20R,22R)−20,22−dihydroxycholesterol
Uridine
17alpha−Hydroxypregnenolone
9S−HOTrE
17−Hydroxylinolenic acid
Stearidonic acid
UDP−N−acetyl−D−mannosamine
PGF2alpha
5−Thymidylic acid
1.5
1
0.5
0
−0.5
−1
−1.5
Group
Model
YGPs
b
Model
YGPs
NZEP TA−Ce
(20R,22R)−20,22−dihydroxycholesterol
Aldosterone
9S−HOTrE
Stearidonic acid
1.5
1
0.5
0
−0.5
−1
−1.5
Group
Model
YGPs
NZEP TA-Ce
c
KEGG Terms
Steroid hormone biosynthesis
Tyrosine metabolism
Alpha−Linolenic acid metabolism
Amino sugar and nucleotide sugar metabolism
Arachidonic acid metabolism
Butanoate metabolism
Pyrimidine metabolism
Cysteine and methionine metabolism
Linoleic acid metabolism
Aldosterone−regulated sodium reabsorption
GABAergic synapse
Cortisol synthesis and secretion
Aldosterone synthesis and secretion
Cushing syndrome
CAMP signaling pathway
YGPs vs Model
Metabolism
Organismal Systems
Human Diseases
Environmental Information Processing
0.0
0.5
1.0
1.5
2.0
−log10(P value)

Supplementary Figure 31. Clustering and KEGG pathway enrichment analysis of differential metabolites. (**a**) Heat map of the total differential metabolite clustering in the Model versus YGPs group. Each column represents one mouse, n = 3 independent samples. (**b**) Heat map of the total differential metabolite clustering between Model, YGPs and NZEP$^{TA\text{-}Ce}$ group. Each column represents one mouse, n = 3 independent samples. (**c**) KEGG enrichment plot of the differential metabolites in the Model and YGPs group.

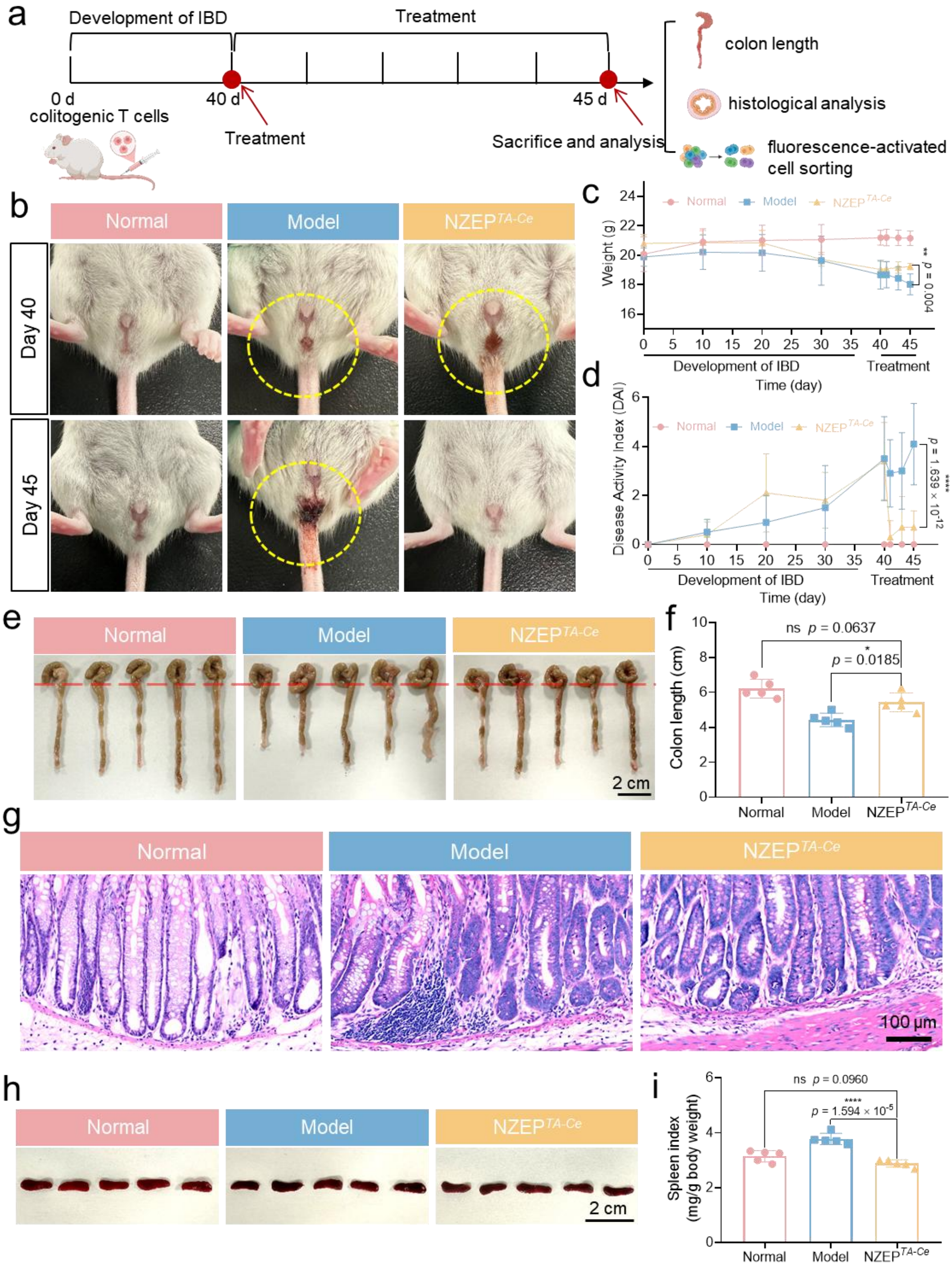

Supplementary Figure 32. Therapeutic effects of NZEP$^{TA\text{-}Ce}$ in the CD4$^+$CD45RB^{hi} T cells induced IBD model. (**a**) Schematic of the treatment plan. (**b**) Representative photographs of the anus during treatment of different groups of IBD mice (created in BioRender. Zhang, F. (2026) https://BioRender.com/6z3zq2c). The yellow dashed circles indicate anal blood residue in mice. (**c**) The weight changes (n = 10 mice). (**d**) DAI score during the experiment (n = 10 mice). (**e**) Colon

images and (**f**) quantified colon lengths in different treatment groups (n = 5 mice). (**g**) Representative H&E staining colon tissue images. (**h**) Spleen images and (**i**) quantified spleen index in different treatment groups (n = 5 mice). In (b), (e) and (g), data are from three independent experiments. In (c) and (d), Two-way ANOVA followed by Tukey's posttest. In (f) and (i), one-way ANOVA followed by Tukey's posttest. Unless otherwise stated, data are presented as means ± SD. *$p < 0.05$, **$p < 0.01$, ***$p < 0.001$, and ****$p < 0.0001$.

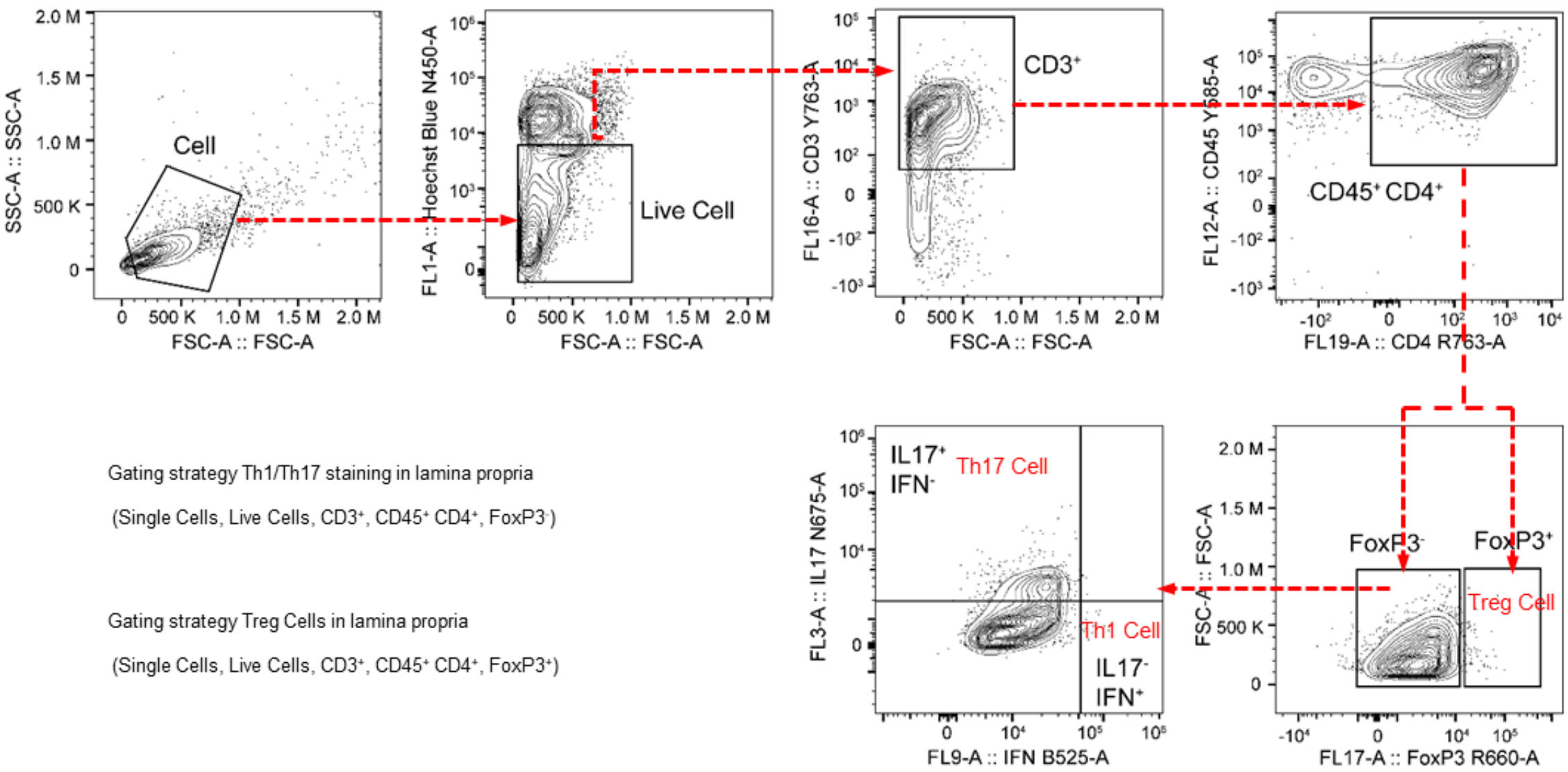

Supplementary Figure 33. Gating strategies for FACS analyses of different cells in lamina propria.

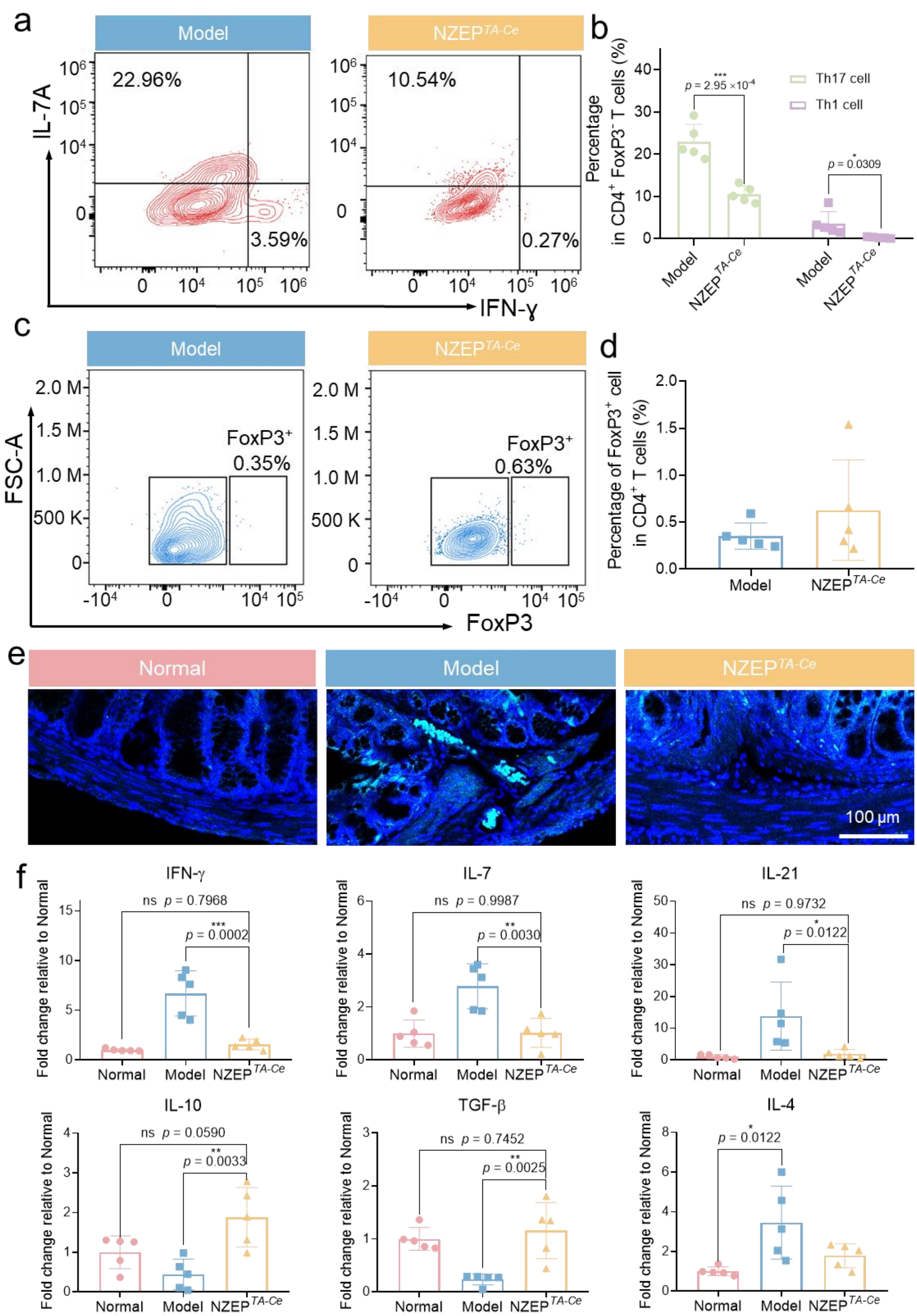

Supplementary Figure 34. Effects of NZEP$^{TA\text{-}Ce}$ on immune cells in the lamina propria in the CD4$^+$CD45RB^{hi} T cells induced IBD model. (**a**) Representative images of Th17 and Th1 cell subsets and (**b**) statistical data (n = 5 mice). (**c**) Representative images of Treg cell subsets and (**d**) statistical data (n = 5 mice). (**e**) Representative immunofluorescence staining for IL-7 of colonic

tissue. (**f**) The relative expression levels of IFN-γ, IL-17, IL-21, IL-10, TGF-β, and IL-4 were determined using ELISA (n = 5 mice). Three independent experiments were performed and representative results are shown in (e). In (b), (d) and (f), one-way ANOVA followed by Tukey's posttest. Unless otherwise stated, data are presented as means ± SD. *$p < 0.05$, **$p < 0.01$, ***$p < 0.001$, and ****$p < 0.0001$.

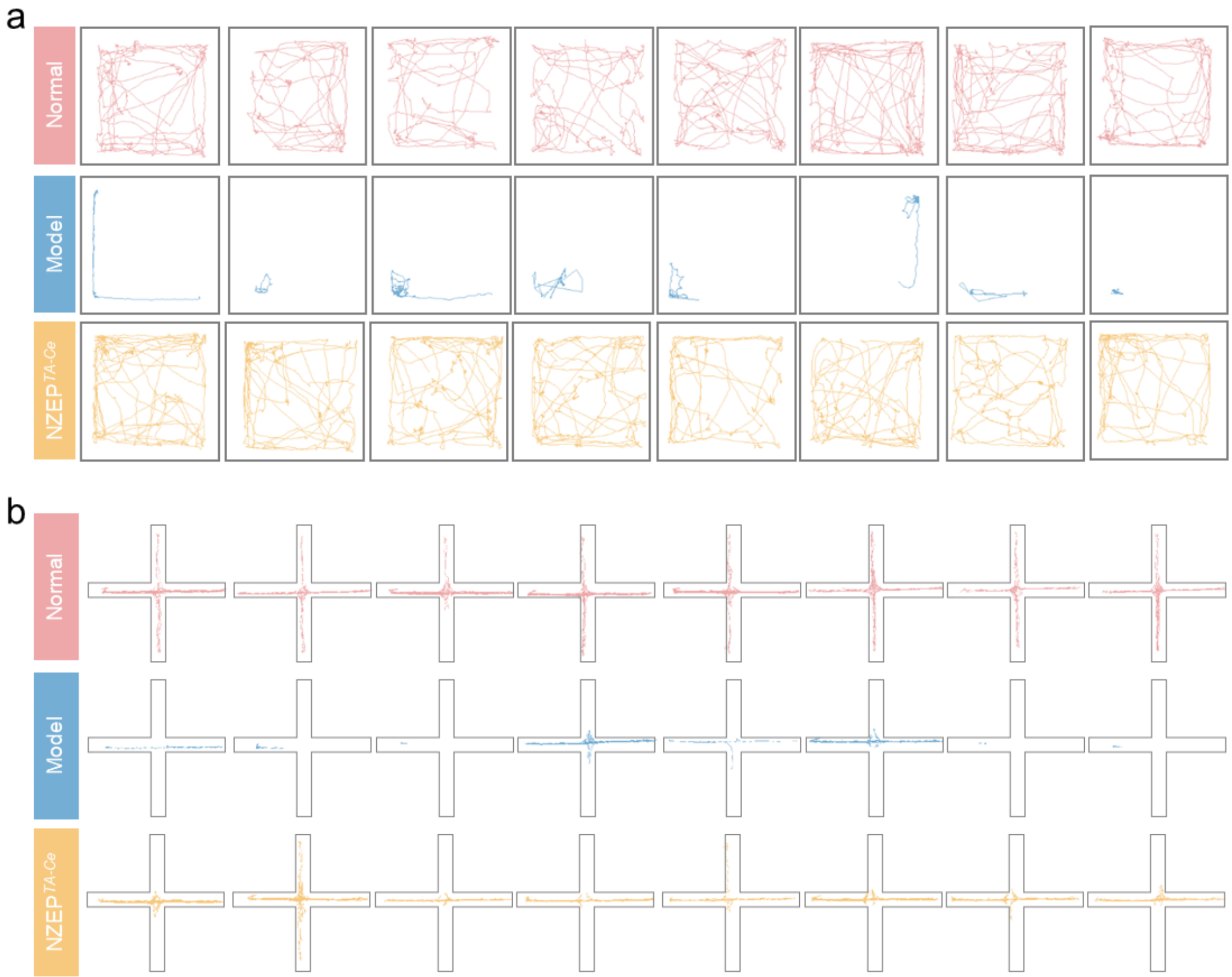

Supplementary Figure 35. Movement trajectories in OFT and EPM behavioral tests. (**a**) Movement tracking images of mice in the OFT (n = 8 mice). (**b**) Movement tracking images of mice in the EPM test (n = 8 mice).

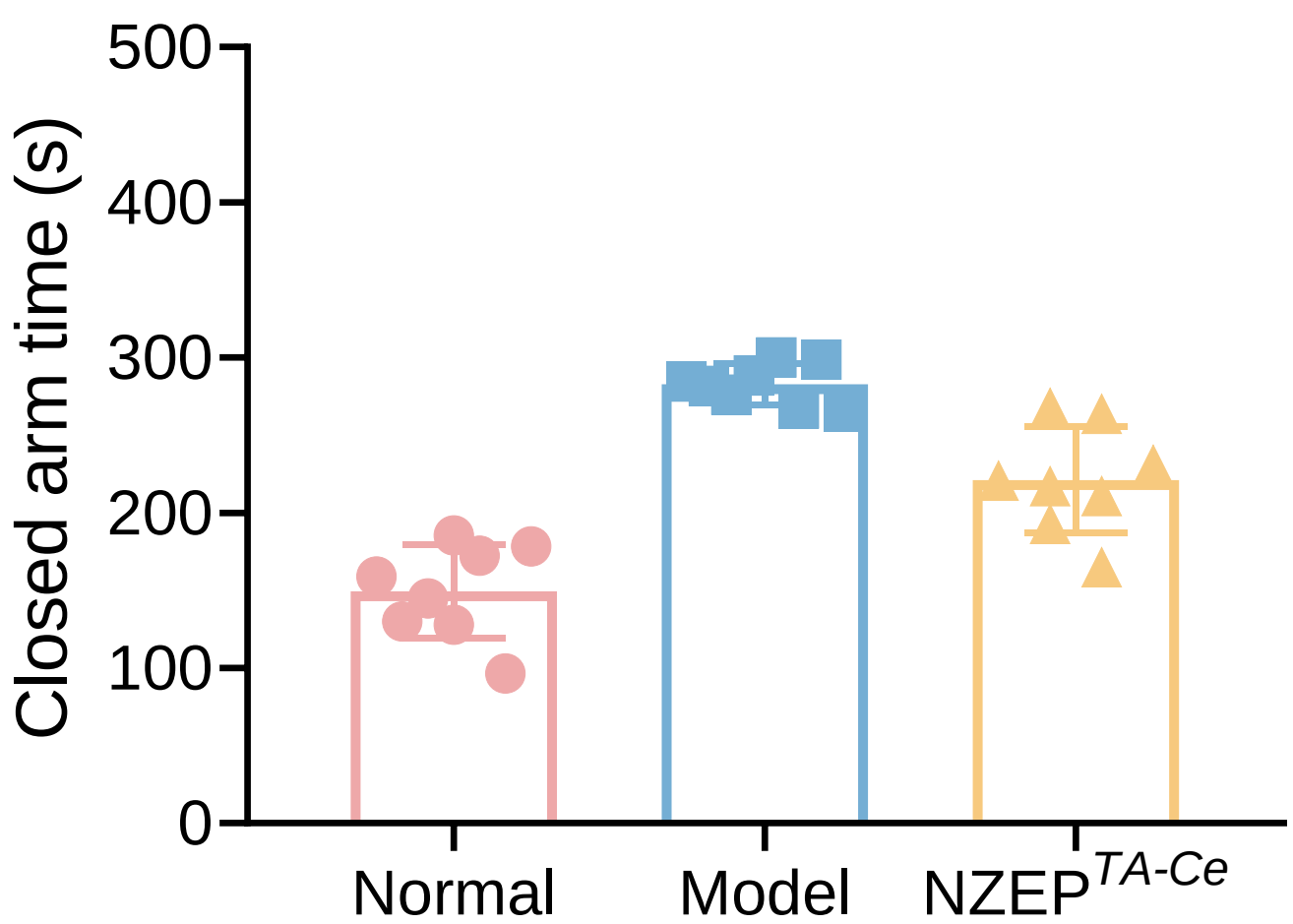

Supplementary Figure 36. Time spent in closed arm in the EPM test (n = 8 mice).

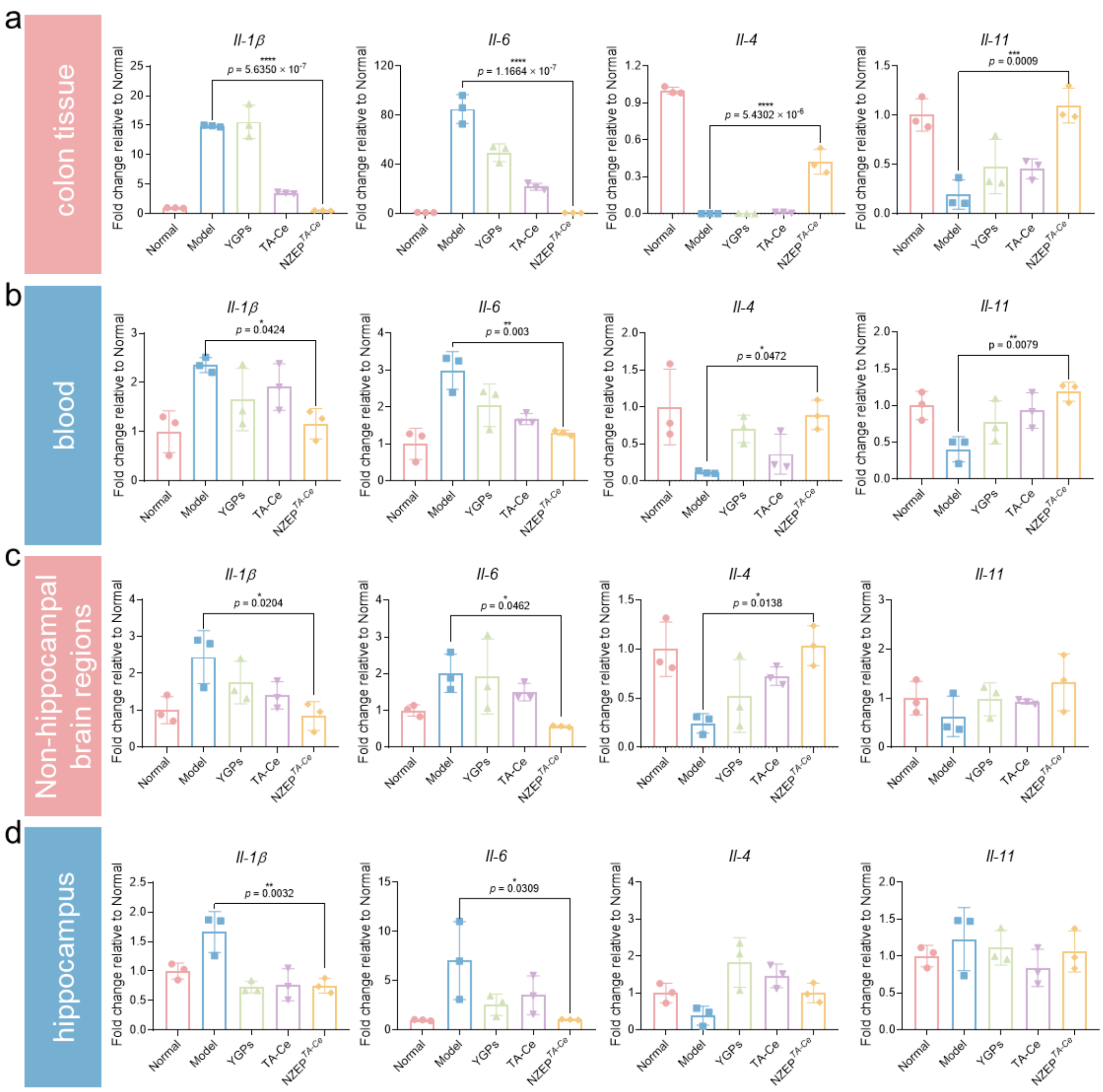

Supplementary Figure 37. Cytokine expression profiles in DSS-induced IBD model. mRNA expression of *Il-1β*, *Il-6*, *Il-4* and *Il-11* in (**a**) colon tissue, (**b**) blood, (**c**) non-hippocampal brain regions and (**d**) hippocampus (n = 3 mice). Data are presented as mean ± SD and statistical significance was analyzed via one-way ANOVA with Tukey's multiple comparison test. *$p < 0.05$, **$p < 0.01$, ***$p < 0.001$, ****$p < 0.0001$.

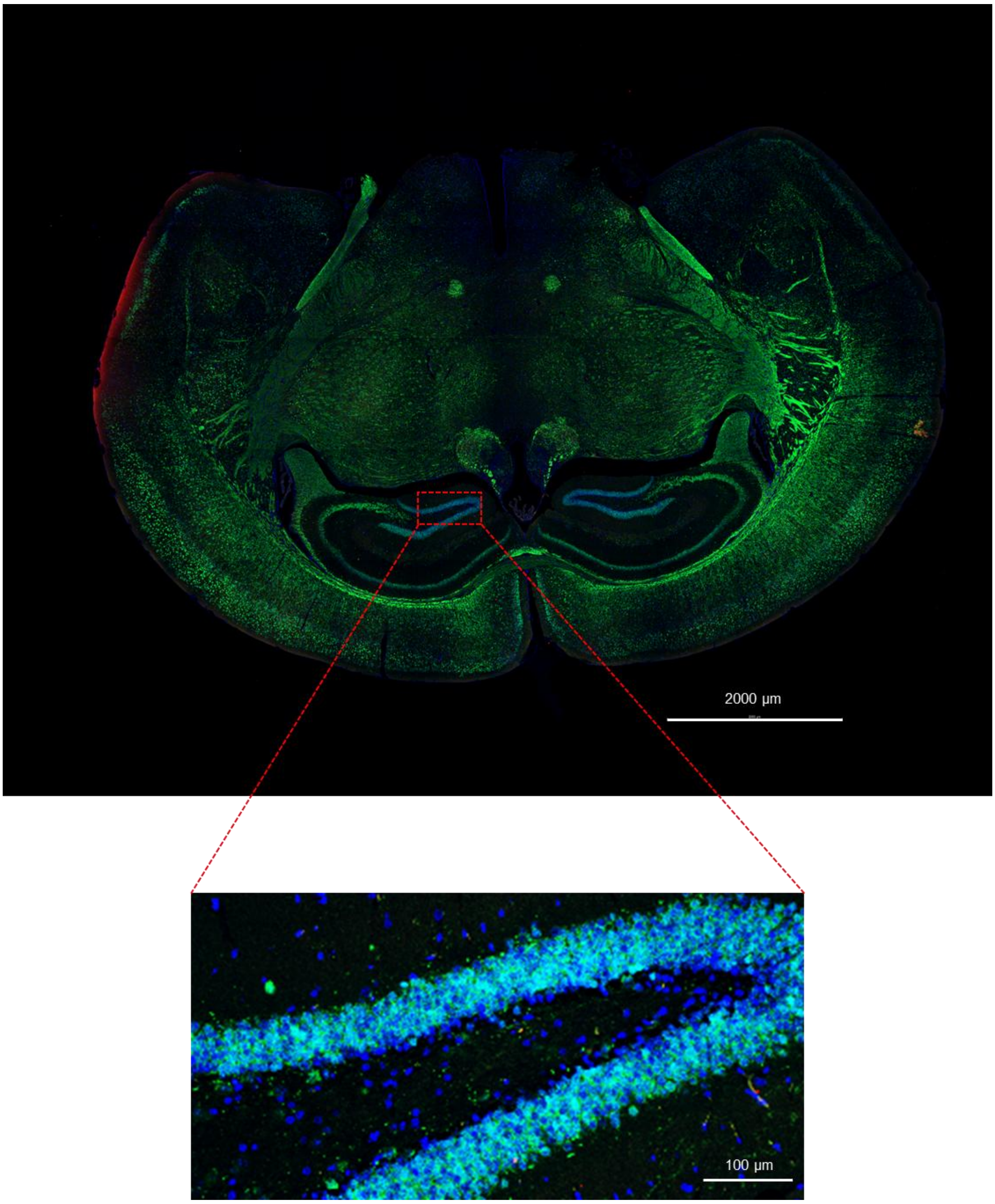

Supplementary Figure 38. Full-field immunofluorescence image of brain, showing BrdU (red), NeuN (FITC), and nuclei stained with DAPI (blue). The red dashed box denotes the region of interest (ROI) corresponding to the magnified view presented in the Figure 10l Normal group.

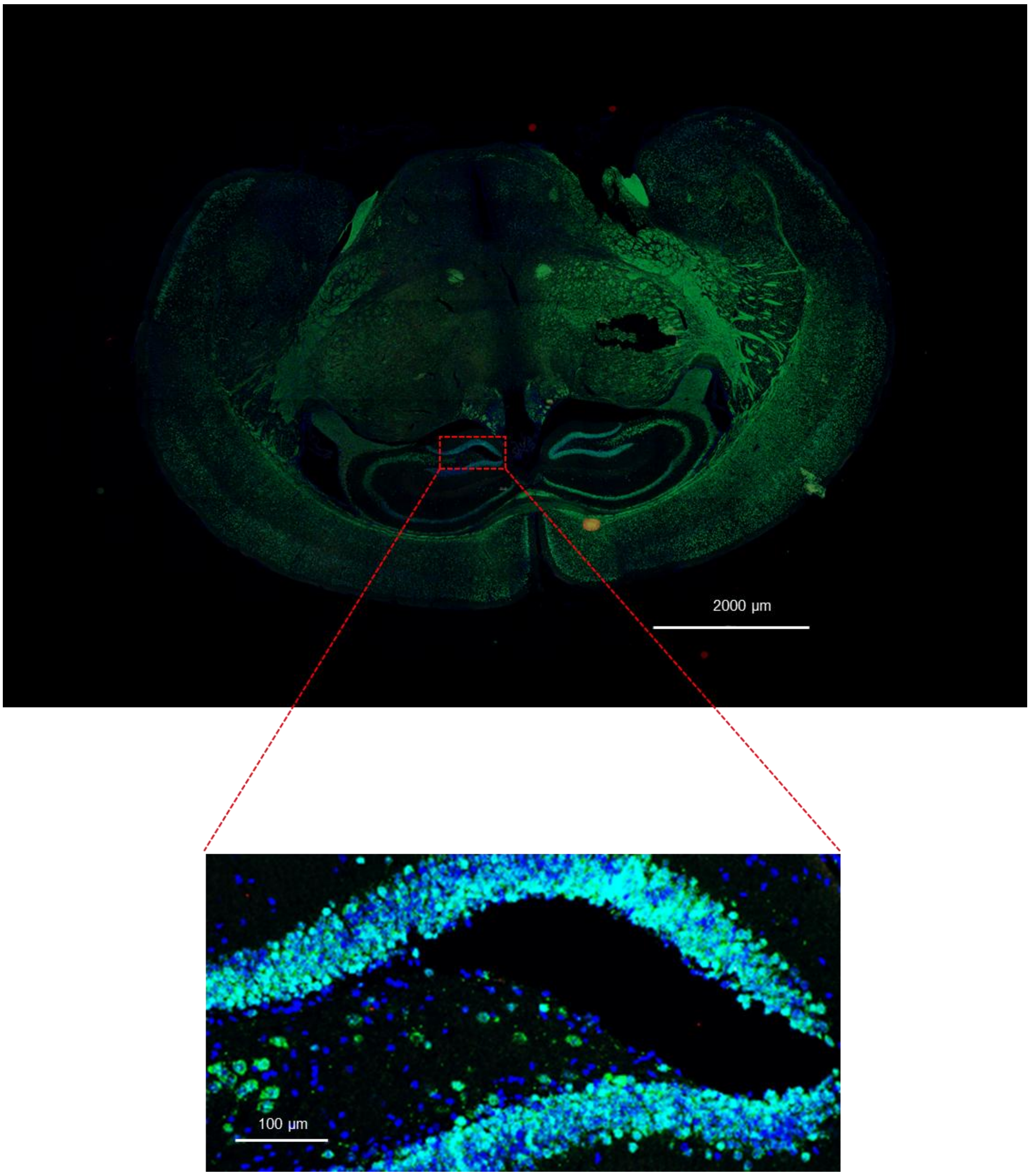

Supplementary Figure 39. Full-field immunofluorescence image of brain, showing BrdU (red), NeuN (FITC), and nuclei stained with DAPI (blue). The red dashed box denotes the ROI corresponding to the magnified view presented in the Figure 10l Model group.

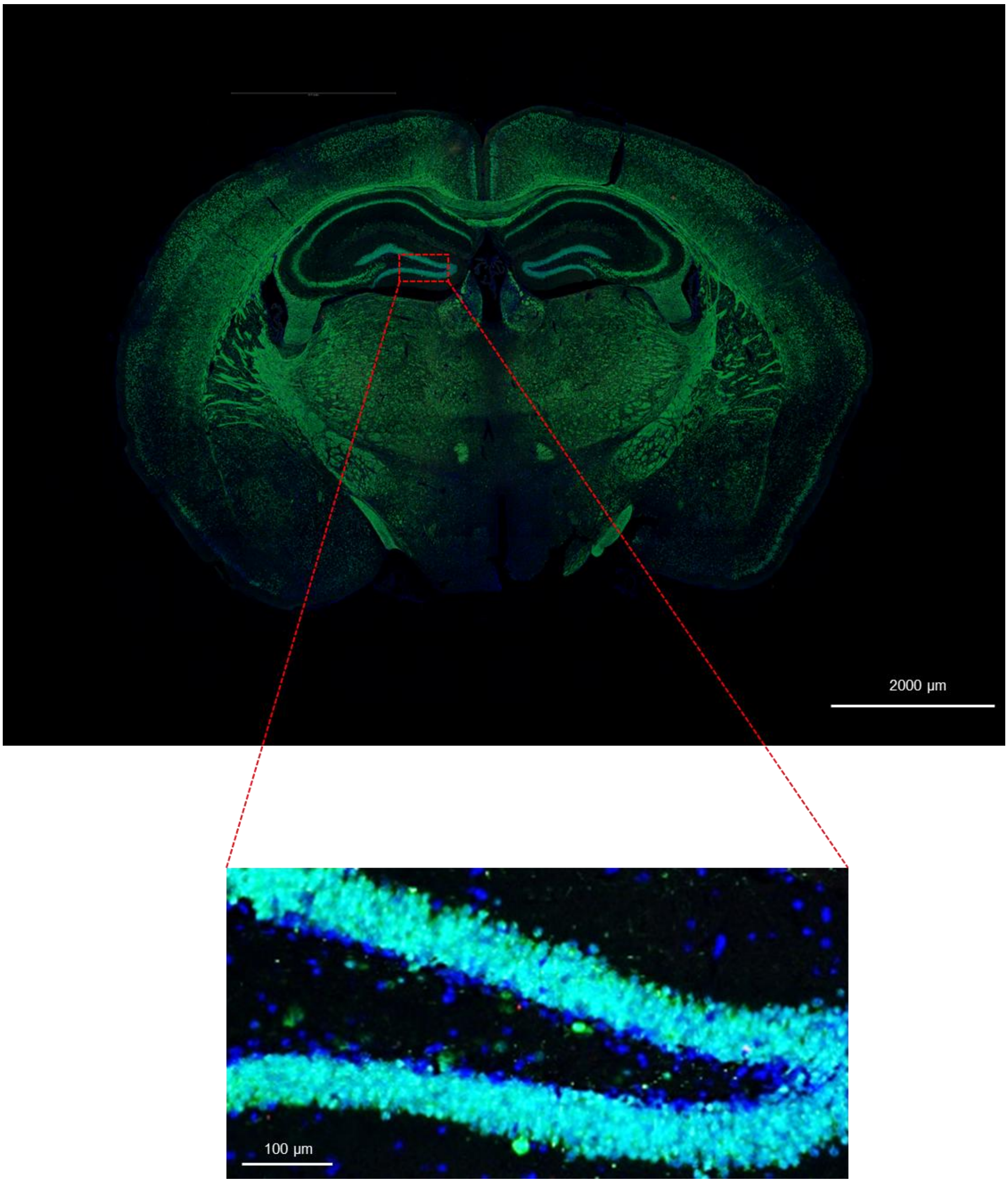

Supplementary Figure 40. Full-field immunofluorescence image of brain, showing BrdU (red), NeuN (FITC), and nuclei stained with DAPI (blue). The red dashed box denotes the ROI corresponding to the magnified view presented in the Figure 10l NZEP$^{TA\text{-}Ce}$ group.

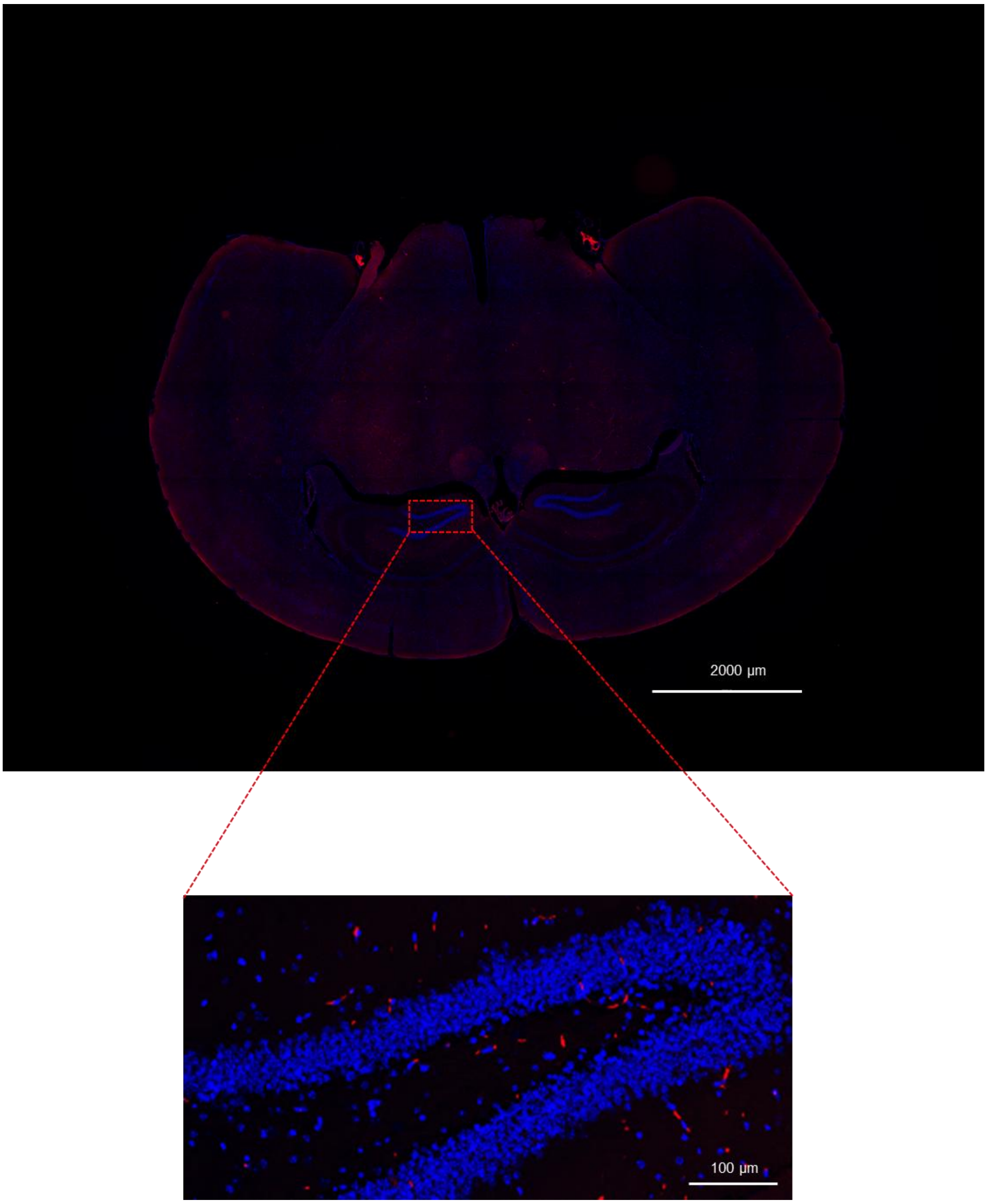

Supplementary Figure 41. Full-field immunofluorescence image of brain, showing DCX (red) and nuclei stained with DAPI (blue). The red dashed box denotes the ROI corresponding to the magnified view presented in the Figure 10m Normal group.

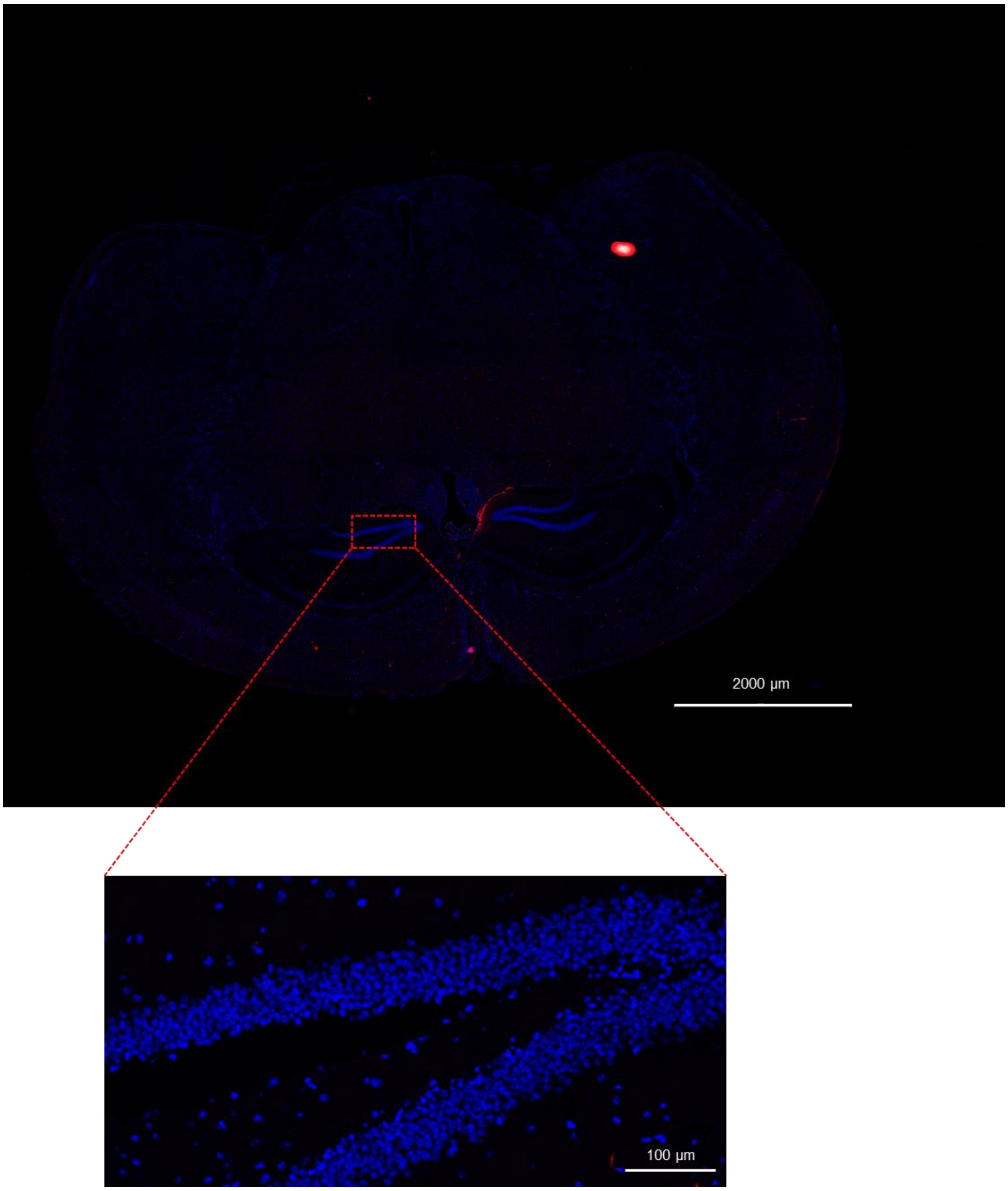

Supplementary Figure 42. Full-field immunofluorescence image of brain, showing DCX (red) and nuclei stained with DAPI (blue). The red dashed box denotes the ROI corresponding to the magnified view presented in the Figure 10m Model group.

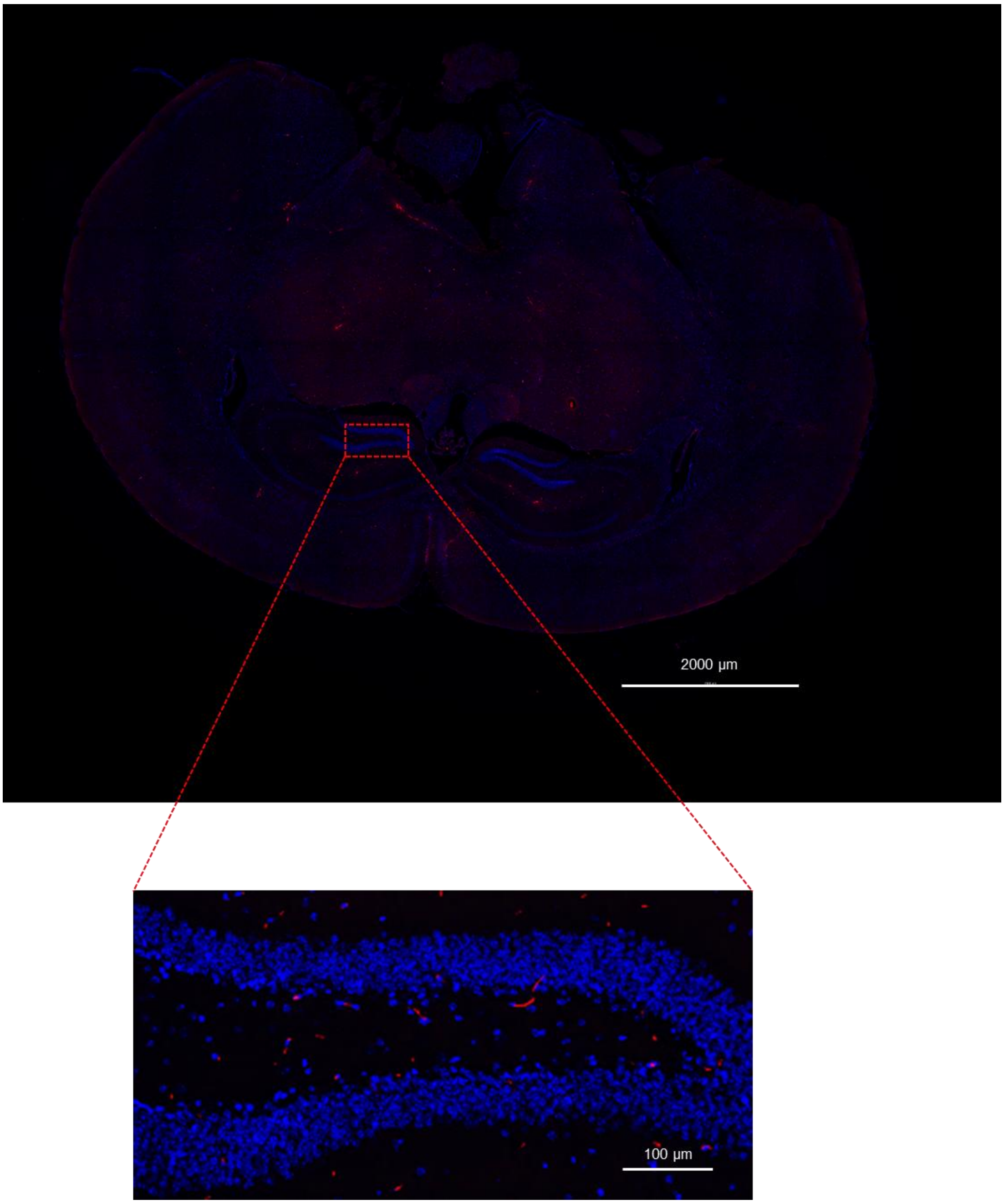

Supplementary Figure 43. Full-field immunofluorescence image of brain, showing DCX (red) and nuclei stained with DAPI (blue). The red dashed box denotes the ROI corresponding to the magnified view presented in the Figure 10m NZEP$^{TA\text{-}Ce}$ group.

Supplementary Table 1. Primer sequences in RT-PCR

Primer name	Forward primer (5' to 3')	Reverse primer (5' to 3')
CD86	TGTTTCCGTGGAGACGCAAG	TTGAGCCTTTGTAAATGGGCA
CD206	CTGGGGTTCCATCACTCCAC	AATCAGTGGTGGCCCTATGC
IL-1α	ACGTCAAGCAACGGGAAGAT	AAGGTGCTGATCTGGGTTGG
IL-1β	AAATGCCACCTTTTGACAGTG	TGGATGCTCTCATCAGGACAG
TNF-α	GAACACGTCGTGGGATAATG	GGCAGACTTTGGATGCTTCTT
iNOS	TTCTCAGCCCAACAATACAAGA	GTGGACGGGTCGATGTCAC
IL-11	GAACTGTGTTTGTCGCCTGG	TGCTCGAGGGTCTGAAGAGA
IL-4	CCATATCCACGGATGCGACA	CGTTGCTGTGAGGACGTTTG
IL-6	TTCCTCTGGTCTTCTGGAGT	TCTGTGACTCCAGCTTATCTCTTG
TGFβ3	GGACTTCGGCCACATCAAGA	ATAGGGGACGTGGGTCATCA
Fema1a	GTGCCCCTCGCGGTAAAAAC	GTGCCCCTCGCGGTAAAAAC
Akap1	ACGAGACCGTGCCTGAAAAT	CACTTGTGCCACAGTCTTGC
GAPDH	GTCAAGGCCGAGAATGGGAA	CTCGTGGTTCACACCCATCA

Supplementary Table 2. Histological scores in the colon tissues of DSS colitis mice.

Inflammation		Depth of injury		Crypt damage		Percentage involved	
0	None	0	None	0	None	×1	0% ~ 25%
1	Slight	1	Mucosa	1	1/3 damaged	×2	26% ~ 50%
2	Moderate	2	Mucosa and submucosa	2	2/3 damaged	×3	51% ~ 75%
3	Severe	3	Transmural	3	Only surface epithelium intact	×4	76% ~ 100%
				4	Entire crypt and epithelium lost		

Supplementary Table 3. Antibody information for FACS experiment

Antibody name	Manufacturer	Catalog
CD3e Monoclonal Antibody (145-2C11), PE-Cyanine7,	ThermoFisher Scientific	25-0031-81
CD4 Monoclonal Antibody (RM4-5), APC-eFluor ™ 780,	ThermoFisher Scientific	47-0042-80
IL-17A Monoclonal Antibody (eBio17B7), Brilliant Ultra Violet™ 615	ThermoFisher Scientific	366-7177-80
IFN gamma Monoclonal Antibody (XMG1.2), FITC,	ThermoFisher Scientific	11-7311-81
CD25 Monoclonal Antibody (PC61.5), PerCP-Cyanine5.5,	ThermoFisher Scientific	45-0251-80
FOXP3 Monoclonal Antibody (FJK-16s), eFluor ™ 660,	ThermoFisher Scientific	50-5773-80
PE Anti-Mouse CD45.1 Antibody	Elabscience	E-AB-F1184D

Supplementary Table 4. Reagent information for FACS experiment

Reagent name	Manufacturer	Catalog
Intracellular Fixation/Permeabilization Buffer Kit	Elabscience	E-CK-A109
collagenase IV	ThermoFisher Scientific	17104019
Hanks Buffered Salt Solution	solarbio	H1025
DNase I	Roche	10104159001
Percoll	Yeasen	40501ES60
Fetal Bovine Serum	Gibco	10099-141
Hoechst 33342	Yeasen	40732ES03

Supplementary Table 5. Concentration of iron and cerium ions in blood.

Group	Number	Fe (μg/mL)	Ce (μg/mL)
Normal	1	62.68	Not Detected
	2	51.21	Not Detected
	3	94.01	Not Detected
Model	1	55.65	Not Detected
	2	76.83	Not Detected
	3	60.63	Not Detected
YGPs	1	74.81	Not Detected
	2	44.59	Not Detected
	3	60.18	Not Detected
TA-Ce	1	79.50	Not Detected
	2	28.90	Not Detected
	3	56.59	Not Detected
NZEP$^{TA\text{-}Ce}$	1	65.50	Not Detected
	2	48.97	Not Detected
	3	62.79	Not Detected

Supplementary Table 6. Comparison of recent major publications on probiotics or prebiotics for IBD with our work

Year	Probiotic or Prebiotic	Construction strategy	Stability	Intestinal retention strategy	Ability to alleviate complications	Ref.
2023	*Escherichia coli Nissle 1917*	Step 1: formation of gelled peritoneal macrophages (GPM)<br>Step 2: coated GPM onto the surface of EcN	Resistant to SGF	Chemical attachment	No	1
2023	*Escherichia coli Nissle 1917*	Step 1: formation of hydrogel<br>Step 2: encapsulate EcN	Resistant to SGF and SIF	Gel adhesion	No	2
2023	*Escherichia coli Nissle 1917*	Step 1: formation of Fe-TA network layer on EcN.<br>Step 2: encapsulated by arboxymethylated β-glucan	Resistant to SGF and bile	Not mentioned	No	3
2023	*Lactobacillus acidophilu*	Step 1: formation of hyaluronic acid grafted with dopamine (HDP)<br>Step 2: coated HDP layer onto the surface of Lac	Not mentioned	Catechol hydroxyl group adhesion	No	4
2024	*Escherichia coli Nissle 1917*	Step 1: formation of polyphenolic nanoparticles (pBDT-TA)<br>Step 2: coated sodium alginate (SA) onto the surface of EcN<br>Step 3: coated pBDT-TA onto the surface of EcN-SA	Resistant to SGF	Coating adhesion	No	5
2024	*Escherichia coli Nissle 1917*	Step 1: formation of Fe^{3+}-containing montmorillonite nanosheets (MMT-Fe)<br>Step 2: encapsulated in the MMT-Fe	Resistant to SGF	Charge effects	No	6
2024	*Escherichia coli Nissle 1917*	Step 1: formation of aldehyde silica microspheres (SAP)<br>Step 2: surface-couple EcN-derived OMVs	Resistant to PBS and SGF	Not mentioned	No	7
2024	*Lactobacillus reuteri DSM 17938*	Step 1: formation of coated with azido (N_3)-modified Prussian blue nanozyme (PB@N_3)<br>Step 2: DBCO group decoration of L. reuteri DSM 17938<br>Step 3: The products of above two steps were mixed	Resistant to HCL	Not mentioned	No	8
2024	*Lactobacillus plantarum*	Step 1: encapsulation by phenolic-metallic networks<br>Step 2: preparation of gels for stable delivery.	Resistant to SGF	Adhesion	No	9

2024	*Bacillus licheniformis*	Step 1: formation of selenium nanoparticles stabilized by yeast glucan<br>Step 2: formation of alginate outer shell	Resistant to SGF and SIF	Not mentioned	No	10
2024	*Bifidobacterium longum*	Step 1: formation of Inulin/TFA/SS NPs<br>Step 2: coated PLL and PGA onto the surface of BL(BL@PP)<br>Step 3: coated Inulin/TFA/SS NPs onto the surface of BL@PP	Resistant to SGF and H_2O_2	Coating adhesion	No	11
2024	Prebiotic protein	Emulsion/solvent evaporation method to develop AST-loaded NPs (AST NPs)	Resistant to SGF	Charge effects	No	12
2024	prebiotic inulin	Hydrogel loaded with polypyrrole (PPy) nanozymes and antifibrotic drug pirfenidone	Resistant to different pH levels	Gel adhesion	No	13
2024	prebiotic inulin	Step 1: formation of mitochondria-targeting liposomes<br>Step 2: encapsulated liposomes in resveratrol and inulin gel	Hierarchical degradation in SGF and SIF	Gel adhesion	No	14
2024	yeast β-glucan	Step 1: self-assembly of yeast β-glucan aceta (YBNs)<br>Step 2: coated with bio-adhesive polydopamine	Resistant to SGF	Coating adhesion	No	15
2025	*Bacillus coagulans*	Step 1: coated rosmarinic acid<br>Step 2: coated silk fibroin	Resistant to SGF and SIF	Coating-mediated recognition	No	16
2025	*Lactobacillus acidophilus*	Step 1: formation of tungsten ion-loaded mesoporous polydopamine (WMPDA)<br>Step 2: encapsulated in WMPDA	Resistant to SGF and SIF	Coating adhesion	No	17
**Our work**	**yeast β-glucan**	**Bioinspired Nanozyme-Exoskeletonized Strategy**	**Resistant to SGF and SIF(IBD)**	**Cell targeted uptake**	**Yes, Anxiety and depressive behaviors**	

Supplementary References

1. Wang J., Hu D., Chen Q., Liu T., Zhou X., Xu Y., et al. Intracellular hydrogelation of macrophage conjugated probiotics for hitchhiking delivery and combined treatment of colitis. *Mater. Today. Bio.* **20**, 100679 (2023).
2. Chen J., Zhang P., Wu C., Yao Q., Cha R., Gao Y. Reductase-Labile Peptidic Supramolecular Hydrogels Aided in Oral Delivery of Probiotics. *ACS. Appl. Mater. Interfaces.* **15**, 31214-31223 (2023).
3. Xie A., Ji H., Liu Z., Wan Y., Zhang X., Xiong H., et al. Modified Prebiotic-Based "Shield" Armed Probiotics with Enhanced Resistance of Gastrointestinal Stresses and Prolonged Intestinal Retention for Synergistic Alleviation of Colitis. *ACS Nano* **17**, 14775-14791 (2023).
4. Huang Q. X., Liang J. L., Yang C. H., Li K., Niu M. T., Fan J. X., et al. Stimulation-responsive mucoadhesive probiotics for inflammatory bowel disease treatment by scavenging reactive oxygen species and regulating gut microbiota. *Biomaterials* **301**, 122274 (2023).
5. Hu Q., Li J., Wang T., Xu X., Duan Y., Jin Y. Polyphenolic Nanoparticle-Modified Probiotics for Microenvironment Remodeling and Targeted Therapy of Inflammatory Bowel Disease. *ACS Nano* **18**, 12917-12932 (2024).
6. Yang J., Ge S., Tan S., Liu H., Yang M., Liu W., et al. Modified montmorillonite armed probiotics with enhanced on-site mucus-depleted intestinal colonization and H(2)S scavenging for colitis treatment. *J. Control. Release.* **374**, 140-153 (2024).
7. Han L., Liu X.-W., Li W.-X., Zang T., Li Z.-P., Deng Y., et al. Innovative probiotic Escherichia coli nissle 1917-derived outer membrane vesicles coupled microspheres demonstrate anti-inflammatory and barrier-enhancing properties in colitis. *Chemical Engineering Journal* **487**, 150711 (2024).
8. Dong F., Hao L., Wang L., Huang Y. Clickable nanozyme enhances precise colonization of probiotics for ameliorating inflammatory bowel disease. *J. Control. Release.* **373**, 749-765 (2024).
9. Wang X. C., Xu Y., Jiang W., Luo F. X., Zhang D., Wu D., et al. Dual-layer probiotic encapsulation using metal phenolic network with gellan gum-tamarind gum coating for colitis treatment. *Int. J. Biol. Macromol.* **280**, 135759 (2024).
10. Yao Y., Xue B., Lessing D. J., Chu W. Probiotics-Loaded Selenium Nanoparticles Effectively Alleviated DSS-Induced Colitis by Scavenging ROS and Remodeling Intestinal Innate Immune and Microbiota. *Advanced Therapeutics* **7**, 2400275 (2024).
11. Zhang Y., Zhang X., Lv L., Gao S., Li X., Wang R., et al. Versatile inulin/trans-ferulic acid/silk sericin nanoparticles-nourished probiotic complex with prolonged intestinal retention for synergistic therapy of inflammatory bowel disease. *Carbohydr. Polym.* **350**, 123063 (2025).
12. Yu Y., Yang D., Lin B., Zhu L., Li C., Li X. Readily Available Oral Prebiotic Protein Reactive Oxygen Species Nanoscavengers for Synergistic Therapy of Inflammation and Fibrosis in Inflammatory Bowel Disease. *ACS Nano* **18**, 13583-13598 (2024).
13. Cao X., Tao S., Wang W., Wu S., Hong Y., Wang X., et al. Ternary inulin hydrogel with long-term intestinal retention for simultaneously reversing IBD and its fibrotic

complication. *Nat. Commun.* **15**, 8428 (2024).

14. Xing L., Liu X., Wu L., Wu J., Deng Y., Li Q., et al. Orally hierarchical targeting delivery systems relieve colitis by protecting host mitochondria and modulating gut microbiota. *Nano Today* **55**, 102155 (2024).
15. Yang F., Su Y., Yan C., Chen T., Cheung P. C. K. Attenuation of inflammatory bowel disease by oral administration of mucoadhesive polydopamine-coated yeast beta-glucan via ROS scavenging and gut microbiota regulation. *J. Nanobiotechnology.* **22**, 166 (2024).
16. Huang Y., Cai H., Liu H., Wang L., Feng G., Ding Z., et al. Probiotic nanocomposite materials with excellent resistance, inflammatory targeting, and multiple efficacies for enhanced treatment of colitis in mice. *J. Nanobiotechnology.* **23**, 188 (2025).
17. Deng J., Hu Y., Zhu P., Yu Y., Chen Q., Wu H., et al. Probiotic Delivery for Editing of the Gut Microbiota to Mitigate Colitis and Maintain Hepatic Homeostasis Via Gut-Liver Axis. *ACS Nano* **19**, 10500-10514 (2025).
