# Supplementary material for: Bioinspired nanozyme-exoskeletonized yeast-derived polysaccharides for treating comorbid anxiety and depression in inflammatory bowel disease
Source: Nat Commun. 2026 Oct 14;17:11594. doi: 10.1038/s41467-026-78633-3 (PMC13752268; doi:10.1038/s41467-026-78633-3)
Supplement: Supplementary file 2 [file 41467_2026_78633_MOESM2_ESM.pdf]

nature portfolio

Corresponding author(s): Chen Lei, Liu Yong, Xiao Jian

Last updated by author(s): May 1, 2026

Reporting Summary

Nature Portfolio wishes to improve the reproducibility of the work that we publish. This form provides structure for consistency and transparency in reporting. For further information on Nature Portfolio policies, see our Editorial Policies and the Editorial Policy Checklist.

Statistics

For all statistical analyses, confirm that the following items are present in the figure legend, table legend, main text, or Methods section.

n/a	Confirmed	
☐	☒	The exact sample size (n) for each experimental group/condition, given as a discrete number and unit of measurement
☐	☒	A statement on whether measurements were taken from distinct samples or whether the same sample was measured repeatedly
☐	☒	The statistical test(s) used AND whether they are one- or two-sided<br>*Only common tests should be described solely by name; describe more complex techniques in the Methods section.*
☐	☒	A description of all covariates tested
☐	☒	A description of any assumptions or corrections, such as tests of normality and adjustment for multiple comparisons
☐	☒	A full description of the statistical parameters including central tendency (e.g. means) or other basic estimates (e.g. regression coefficient) AND variation (e.g. standard deviation) or associated estimates of uncertainty (e.g. confidence intervals)
☐	☒	For null hypothesis testing, the test statistic (e.g. F, t, r) with confidence intervals, effect sizes, degrees of freedom and P value noted<br>*Give P values as exact values whenever suitable.*
☒	☐	For Bayesian analysis, information on the choice of priors and Markov chain Monte Carlo settings
☒	☐	For hierarchical and complex designs, identification of the appropriate level for tests and full reporting of outcomes
☒	☐	Estimates of effect sizes (e.g. Cohen's d, Pearson's r), indicating how they were calculated

Our web collection on statistics for biologists contains articles on many of the points above.

Software and code

Policy information about availability of computer code

Data collection	Beckman Coulter CytoFlex LX for flow cytometry analysis, Olympus VS200 for histological slices, Zeiss Zen Blue Edition (version 3.6.1) for confocol images.
Data analysis	GraphPad Prism (version 8.2.1) and Image J (version 2.1.0), FlowJo (version 10).

For manuscripts utilizing custom algorithms or software that are central to the research but not yet described in published literature, software must be made available to editors and reviewers. We strongly encourage code deposition in a community repository (e.g. GitHub). See the Nature Portfolio guidelines for submitting code & software for further information.

Data

Policy information about availability of data

All manuscripts must include a data availability statement. This statement should provide the following information, where applicable:

- Accession codes, unique identifiers, or web links for publicly available datasets
- A description of any restrictions on data availability
- For clinical datasets or third party data, please ensure that the statement adheres to our policy

All data generated or analysed during this study are included in this published article and its Supplementary Information files. Source data underlying Figs. 1-10 and Supplementary Figs. 1-43 are available in Figshare (https://doi.org/10.6084/m9.figshare.28674176). RNA-seq data from RAW264.7 cells and mouse fecal 16S rRNA gene sequencing data have been deposited in the NCBI BioProject database under accession codes PRJNA1442071 and PRJNA1442011, respectively, with raw sequencing data available in the Sequence Read Archive (SRA). Metabolomics data have been deposited in the MetaboLights repository under accession code

MTBLS14127 (https://www.ebi.ac.uk/metabolights/MTBLS14127). All data underlying this study are available from the corresponding author upon request.

Research involving human participants, their data, or biological material

Policy information about studies with human participants or human data. See also policy information about sex, gender (identity/presentation), and sexual orientation and race, ethnicity and racism.

Reporting on sex and gender	n/a
Reporting on race, ethnicity, or other socially relevant groupings	n/a
Population characteristics	n/a
Recruitment	n/a
Ethics oversight	n/a

Note that full information on the approval of the study protocol must also be provided in the manuscript.

Field-specific reporting

Please select the one below that is the best fit for your research. If you are not sure, read the appropriate sections before making your selection.

[x] Life sciences [ ] Behavioural & social sciences [ ] Ecological, evolutionary & environmental sciences

For a reference copy of the document with all sections, see nature.com/documents/nr-reporting-summary-flat.pdf

Life sciences study design

All studies must disclose on these points even when the disclosure is negative.

Sample size	Sample size was determined based on mean and standard deviation data from our preliminary tests.
Data exclusions	No data was excluded from the analyses
Replication	All attempts at replication were successful. TEM, SEM, imaging and immunological staining experiments have been replicated at least three times independently to ensure reproducibility of results.
Randomization	All experimental units were randomly allocated across the treatment groups.
Blinding	The investigators were blinded to all group allocation during data collection and/or analysis.

Reporting for specific materials, systems and methods

We require information from authors about some types of materials, experimental systems and methods used in many studies. Here, indicate whether each material, system or method listed is relevant to your study. If you are not sure if a list item applies to your research, read the appropriate section before selecting a response.

Materials & experimental systems

n/a	Involved in the study	
[ ]	[x]	Antibodies
[ ]	[x]	Eukaryotic cell lines
[x]	[ ]	Palaeontology and archaeology
[ ]	[x]	Animals and other organisms
[x]	[ ]	Clinical data
[x]	[ ]	Dual use research of concern
[x]	[ ]	Plants

Methods

n/a	Involved in the study	
[x]	[ ]	ChIP-seq
[ ]	[x]	Flow cytometry
[x]	[ ]	MRI-based neuroimaging

Antibodies

Antibodies used	Antibodies used for Immunofluorescence staining:<br>CD86 Rabbit pAb (Catalog # A16805) was purchased from ABclonal(China).<br>TLR2 Monoclonal antibody (Catalog # 66645-1-Ig), Anti-Mouse CD11b (M1/70) (Catalog # 65055-1-Ig), CD206 antibody (Catalog # 18704-1-AP), CD68 antibody (Catalog # 66231-2-Ig), zonula occludens-1 (Catalog # 21773-1-AP) and Occludin (Catalog # 66378-1-Ig) and IL-17A antibody (Catalog # 13082-1-AP) were purchased from Proteintech(China).

	Anti-Dectin-1 antibody (Catalog # ab287961) was purchased from abcam.<br>Antibodies used for flow cytometry:<br>PE Anti-Mouse CD45.1 Antibody (Catalog # E-AB-F1184D) was purchased from Elabscience,<br>CD3e Monoclonal Antibody, PE-Cyanine7 (Catalog # 25-0031-81), CD4 Monoclonal Antibody, APC-eFluor™ 780 (Catalog # 47-0042-80), IL-17A Monoclonal Antibody, Brilliant Ultra Violet™ 615 (Catalog # 366-7177-80), IFN gamma Monoclonal Antibody (XMG1.2), FITC (Catalog # 11-7311-81), CD25 Monoclonal Antibody (PC61.5), PerCP-Cyanine5.5 (Catalog # 45-0251-80), FOXP3 Monoclonal Antibody (FJK-16s), eFluor™ 660 (Catalog # 50-5773-80) were purchased from Thermo Fisher.<br>Antibodies used for another:<br>Anti-mDectin-1-IgG (InvivoGen, Catalog # mabg-mdect-2), MAb-mTLR2 (InvivoGen, Catalog # mab2-mtlr2),Ultra-LEAF™ Purified anti-mouse CD18 Antibody (Biolegend, Catalog # 101417),Ultra-LEAF™ Purified anti-mouse/human CD11b Antibody (Biolegend, Catalog # 101247)
Validation	These antibodies have been verified by the supplier and reported for the use in flow cytometry and Immunofluorescence. All the antibodies used are from commercial sources and validation data are available on the manufacturer's website based on their catalogue numbers. The manufacturers include, Abcam, ABclonal, Proteintech, ,ThermoFisher Scientific,BioLegend.

Eukaryotic cell lines

Policy information about cell lines and Sex and Gender in Research

Cell line source(s)	Mouse monocyte macrophage leukemia (RAW264.7) cells (Cat. No. CL-0190) and human colorectal adenocarcinoma (Caco2) cells (Cat. No. CL-0050) at were purchased from Procell Life Science & Technology Co., Ltd.
Authentication	All cell lines were authenticated by the supplier using Short Tandem Repeat test.
Mycoplasma contamination	All the cells were tested and not contaminated with mycoplasma.
Commonly misidentified lines<br>(See ICLAC register)	No commonly misidentified cell lines were used in this study.

Animals and other research organisms

Policy information about studies involving animals; ARRIVE guidelines recommended for reporting animal research, and Sex and Gender in Research

Laboratory animals	C57BL/6J mice (male, 6–8 weeks, 20–22 g) were sourced from Zhejiang Vital River Laboratory Animal Technology Co., Ltd. The CD4+CD45RBhi T cell transfer-induced IBD model was established by Biocytogen Pharmaceuticals (Beijing) Co., Ltd. All animals were housed under specific pathogen-free (SPF) conditions with a 12 h light/12 h dark cycle, controlled ambient temperature (22 ± 2 °C), and relative humidity of 50 ± 10%, with free access to food and water.
Wild animals	The study did not involve wild animals.
Reporting on sex	Sex was reported for each animal model; however, the study was not designed to compare sex-dependent differences, as different models employed sex-specific cohorts according to predefined experimental conditions.
Field-collected samples	No field collected samples were used in the study.
Ethics oversight	All animal experiments were conducted in compliance with the Chinese National Standard and were approved by the Laboratory Animal Ethics Committee of Oujiang Laboratory (No. OJLAB24031201 and OJLAB24082701).

Note that full information on the approval of the study protocol must also be provided in the manuscript.

Plants

Seed stocks	n/a
Novel plant genotypes	n/a
Authentication	n/a

Flow Cytometry

Plots

Confirm that:

- [x] The axis labels state the marker and fluorochrome used (e.g. CD4-FITC).
- [x] The axis scales are clearly visible. Include numbers along axes only for bottom left plot of group (a 'group' is an analysis of identical markers).
- [x] All plots are contour plots with outliers or pseudocolor plots.
- [x] A numerical value for number of cells or percentage (with statistics) is provided.

Methodology

Sample preparation	Fresh mice colon lamina propria samples were rinsed with PBS, then the samples were clipped with sterile curved scissors. Treated cells were washed and stained with fluorescent probe oror antibodies following the manufacturer's instructions. For intracellular staining, the cells were treated by fixation and permeation buffers before antibody staining.
Instrument	Beckman Coulter CytoFlex LX
Software	FlowJo (version 10)
Cell population abundance	10,000 cells were counted and analyzed in all experiments.
Gating strategy	FSC-SSC and singlets were pre-gated. All debris was excluded.

- [x] Tick this box to confirm that a figure exemplifying the gating strategy is provided in the Supplementary Information.
