# Supplementary material for: Bioinspired nanozyme-exoskeletonized yeast-derived polysaccharides for treating comorbid anxiety and depression in inflammatory bowel disease
Source: Nat Commun. 2026 Oct 14;17:11594. doi: 10.1038/s41467-026-78633-3 (PMC13752268; doi:10.1038/s41467-026-78633-3)
Supplement: Supplementary file 3 [file 41467_2026_78633_MOESM3_ESM.pdf]

nature portfolio

Peer Review File

Bioinspired Nanozyme-Exoskeletonized Yeast-derived Polysaccharides for Treating Comorbid Anxiety and Depression in Inflammatory Bowel Disease

Corresponding Author: Professor Jian Xiao

This file contains all reviewer reports in order by version, followed by all author rebuttals in order by version.

Version 0:

Reviewer comments:

Reviewer #1

(Remarks to the Author)
The manuscript entitled Bioinspired Nanozyme-Exoskeletonized Yeast-derived Polysaccharides for Treating Comorbid Anxiety and Depression in Inflammatory Bowel Disease by Zhang and coworkers reports a nanozyme-exoskeletonized yeast-derived polysaccharides (NZEP-TA-Ce) by integrating TA-Ce nanozymes with yeast-derived polysaccharides. The NZEP-TA-Ce could be retained in inflamed regions in colon, reducing intestinal inflammation, promoting intestinal barrier repair, reversing gut microbiota dysbiosis and alleviating anxiety and depressive symptoms. Although some work has been done, the novelty and integrity are not enough for the publication in Nature Communications.

For the novelty, the concept of this work has been reported recently. For example, "Oral Polyphenol-Armored Nanomedicine for Targeted Modulation of Gut Microbiota-Brain Interactions in Colitis, Science Advances, 2023, 9, adf3887". What are the advantages of your design compared to these reported work?, This paper does not compare with the current work, and lacks the necessary experiments to prove the advantages of the author's design. For example, NZEP-TA-CE is taken orally. Oral medications need to overcome harsh environments such as gastric juices and intestinal fluids, but the authors have not demonstrated this.

Additionally, Yeast-derived nanoparticles (Nano Lett. 2017, 17, 2, 1056–1064; Nature Communications,2022, 13, 110; Bioengineered, 2021, 8325-8335; Int J Pharm.,2021,601,120583) and metal-polyphenol network nanozymes (Nano today, 2023, 53, 102044; Rodox Biology, 2024, 73, 103217; Rare Metals, 2023, 42, 2928) have been widely reported and utilized in inflammatory diseases. The combination of the two offers little innovation. Moreover, the metal-polyphenol network nanozyme in this paper only demonstrates free radical scavenging effects. In other words, replacing it with another nanozyme or antioxidant could achieve the same results described in this paper.

For integrity, the discussion in the article, including the sequencing, mostly focuses on the overall role of NZEP-TA-Ce or the efficacy of YGP, without delving into the individual contributions of each component and their synergistic effects. Such holistic discussion fails to provide readers with adequate guidance and assistance. When addressing the gut-brain axis, the role of NZEP-TA-Ce is only discussed behaviorally, lacking mechanistic studies, thus failing to adequately substantiate the author's hypotheses and conclusions.

Comments:

(1) How does NZEP-TA-Ce reach the colon? How much do they degrade? What is the targeting mechanism? Why do other groups not achieve colonic targeting results?
(2) What is the TA-CE morphology? Is it stable? How to ensure the usage quantity is the same as NZEP-TA-Ce?
(3) In endocytosis experiments, the majority of NZEP-TA-Ce is localized around the cell membrane rather than being endocytosed into the nucleus.
(4) NZEP-TA-Ce endocytic targeting is inadequately demonstrated. Endocytic targeting should also be performed for other intestinal cells. Additionally, the TA-CE group should be included to exclude the effect of TA.
(5) Although sequencing was performed and the potential anti-inflammatory mechanism of NZEP-TA-Ce was identified, the specific component responsible for this effect—whether it is NZEP, TA-CE, TA, or CE—was not clearly discussed by the

authors.
(6) Why can NZEP-TA-Ce target inflammatory areas? How does it resist gastric and intestinal fluids? What is its form after passing through gastric and intestinal fluids? Is it due to a larger dose that it can reach the colon?
(7) TA is also considered to have probiotic effects, thus, the authors seem to overlook this issue during bacterial analysis. What are the respective roles of TA and NZEP? What is their synergistic effect?
(8) Bacteria, inflammatory factors, and neurotransmitters can all cause inflammation in the brain's nervous system. However, merely using BrdU and NeuN double staining and DCX staining does not elucidate the mechanism by which IBD treatment affects anxiety and depression.

Reviewer #2

(Remarks to the Author)
In this study, Zhang et al. devised an elegant Nanozyme exoskeletonized yeast-derived polysaccharide combined with ROS scavenging strategy and tested its efficacy in mouse model of IBD. The resulting NZEP-TA-Ce displayed potent anti-inflammatory effect in DSS induced mouse model of IBD. Furthermore, the treatment reduced DSS induced Intestinal dysbiosis, as well as rectified IBD associated symptoms of anxiety and depression in mice. Overall this is an elegant, well executed study with long standing clinical implications, and deserves publication in Nature Communications. Here are some points that in my opinion can improve the study.
1. Since the primary goal of the study is to find the efficacy of NZEPTA-Ce in IBD, evaluating its potential beneficial effect in another model of colitis may be important. I would suggest that the authors test its efficacy in CD4+CD45RBhi T cells induced colitis model. At the end point of the experiment, Immune cell profiling for Regulatory T cells, Conventional T cells and Th1, Th17 Cytokine production in colonic lamina propria will provide additional insights on the effect of NZEPTA-Ce on Intestinal immune cell population.

2. Fig. 7 is the main experiment evaluating the efficacy of NZEPTA-Ce on DSS induced Colitis model in mice. The figure caption says there are 5 mice per group, but had no information as to how many experiments were done as biological replicates. It is important to have 2-3 biological replicates to have robust conclusion of the beneficial effect of the drug.

Minor points:
1. There are too many Supplementary figures, many of which can be clubbed together to single figures.
2. Line 176, I guess there should be a stop after “strategy”, and “chitin” is a miss-insertion.
3. Fig. 6g, “Colnon” should be “Colon”.

Reviewer #3

(Remarks to the Author)
This is a nice paper in which the authors synthesized a novel type of yeast cell-derived drug for treating IBD and mental disorders. The authors have very carefully designed and performed experiments and the results are convincing and encouraging. Overall, I would like to recommend the publication of the work in this journal after minor revision.

Some specific comments:

The authors need to evaluate the stability of NZEPTA-Ce in simulated gastric fluid (SGF) and simulated intestinal fluid (SIF).

The authors need to explain why such a unique structure of NZEPTA-Ce (as shown in Figure 2h) is beneficial for IBD treatment.

The authors need to investigate the distribution of Ce in NZEPTA-Ce. That is, they need to check whether the interior of NZEPTA-Ce contains Ce elements or not.

The authors need to explain why Baker’s yeast was chosen for the construction of NZEPTA-Ce, but not other types of yeast.

Version 1:

Reviewer comments:

Reviewer #1

(Remarks to the Author)
Tha authors have answered my concerns. I think it can be published.

Reviewer #2

(Remarks to the Author)
The authors have conducted T cell transfer based colitis model and observed a positive effect of NZEPTA-Ce . While the results are promising, there are few aspects of this experiment that needs to be clarified.
First, as opposed to the DSS colitis experiment that was done on C57BL/6 background, it appears that this experiment was

done on BALB/c mice (Sup. Fig 32b, the mice appear to be white).
Second, the Flow cytometry data shown in Sup. Fig. 33 and Sup. Fig. 34 a-d appears to have some problems. For example the Treg gating shows all cells to be Foxp3+, and the small population among Foxp3+ cells that were CD25+, were taken as Tregs. This should not be the case. Out of the CD45+CD4+ gate, there should be a discrete population that should be Foxp3+, and should be considered as Tregs. Also the IFNg+ and IL17+ gating is not right. The authors should gate on Foxp3- and effector CD4 T cells and show IFNg+ and IL17+ cells among them. In this regard it also needs to be mentioned that in Sup. Fig. 34a, there appears to a large population that are IFNg, IL17 double positive. Such double positive population typically are associated with enhanced disease progression, and does not seem to be correlative with disease score upon NZEPTA-Ce treatment. Overall it seems that the flow cytometry analysis has some technical issues.
Lastly, the methods section of the manuscript appears to be too short, and the authors are requested to elaborate on it.

Reviewer #3

(Remarks to the Author)
The authors have fully addressed my concerns.

Version 2:

Reviewer comments:

Reviewer #2

(Remarks to the Author)
I thank the authors for the clarifications. However, I realized there are some technical issues still in analyzing the Flow data. The population that the authors are analyzing as “Live” is Hoechst Blue N450 (Please also rectify the spelling in the representative plot), are actually the dead cells, since this die binds to DNA in dead cells with compromised cell membrane. So the authors should gate on the Hoechst negative population and re do the analysis. The Foxp3+ population should be around 10-20%, and there will be many more CD4+Foxp3- population on which the gating should be done to assess IL17 and IFNg expressing cells.

Version 3:

Reviewer comments:

Reviewer #2

(Remarks to the Author)
I am sorry to keep rectifying the authors, but the analysis is still not right. The authors used CD4+CD45RBhi cells to transfer in recipient SKID mice to induce colitis. So in the analysis part, there should not be so much Foxp3+ cell frequency. The reason in my opinion is the background in the flow staining for Foxp3 is very high. To make myself clear I have recommended my gating recommendation for the authors to try out. Hope this will help. A supplementary PDF file is uploaded in the "Review Attached" section.

Another minor point to point out, in the methods section the authors mentioned “CD4+ T cells were first enriched using the CD4+ T Cell Isolation Kit (Miltenyi Biotec, Cat# 130-104-454), followed by positive selection of CD45+RBhi T cells using the CD25 MicroBead Kit (Miltenyi Biotec, Cat# 130-091-072), resulting in purified CD4+CD45RBhi T cells.” I don’t understand how CD45RBhi cells were positively select ed from CD25 isolation kit. Was that a typo? The catalog number is for CD25 isolation kit though.

Version 4:

Reviewer comments:

Reviewer #2

(Remarks to the Author)
The authors have addressed all my concerns.

Open Access This Peer Review File is licensed under a Creative Commons Attribution 4.0 International License, which permits use, sharing, adaptation, distribution and reproduction in any medium or format, as long as you give appropriate credit to the original author(s) and the source, provide a link to the Creative Commons license, and indicate if changes were made.
In cases where reviewers are anonymous, credit should be given to 'Anonymous Referee' and the source.
The images or other third party material in this Peer Review File are included in the article's Creative Commons license, unless indicated otherwise in a credit line to the material. If material is not included in the article's Creative Commons license and your intended use is not permitted by statutory regulation or exceeds the permitted use, you will need to obtain permission directly from the copyright holder.
To view a copy of this license, visit https://creativecommons.org/licenses/by/4.0/
